# Teachers’ assessment literacy improves teaching efficacy: A view from conservation of resources theory

**DOI:** 10.3389/fpsyg.2022.1007830

**Published:** 2022-10-28

**Authors:** Hongxi Wang, Wenwen Sun, Yue Zhou, Tingting Li, Peiling Zhou

**Affiliations:** ^1^Faculty of Education, Henan University, Kaifeng, China; ^2^Institute of Educational Examination and Evaluation, Henan University, Kaifeng, China

**Keywords:** teachers’ assessment literacy, teaching efficacy, psychological capital, professional identity, conservation of resources theory, gain spirals, resource caravans

## Abstract

Recent revisions to the Conservation of Resources theory have not only reclassified categories of resources, but have also acknowledged the conceptual importance of “gain spirals” and “resource caravans” in enriching the theoretical understanding of resources. Given that teachers’ assessment literacy is a prominent yet underexplored personal constructive resource in teaching, this paper examines its role in teaching efficacy. In addition, personal energy resources (e.g., psychological capital and professional identity) are studied as antecedents to teaching efficacy. To this end, a survey based on the Chinese versions of the Teacher Assessment Literacy Scale, the Teaching Efficacy Scale, the Psychological Capital Scale, and the Teacher Professional Identity Scale was administered to secondary school teachers in Henan Province, China, and 351 completed, valid surveys were returned. The findings indicated that the teachers’ assessment literacy and teaching efficacy were positively correlated, verifying that assessment literacy can influence teaching efficacy through the separate and chain mediation effects of psychological capital and professional identity. The identification of such mediating pathways has confirmed that resources owned by teachers can lead to gain spirals and full resource caravans, thus expanding the Conservation of Resources theory by positing that resources can be nested within one another. This study has theoretical implications for teaching efficacy research and the Conservation of Resources theory as well as practical implications regarding how to boost teachers’ constructive and energy resources and professional development.

## Introduction

Occupational stress among teachers has long aroused widespread concern (Kyriacou and Sutcliffe, 1977; Johnson et al., 2005; Newberry and Allsop, 2017; Fynes-Clinton et al., 2022), and the considerable job pressure experienced by teachers is closely related to low teaching efficacy (Betoret, 2006; Klassen and Chiu, 2010). The concept of teaching efficacy refers to teachers’ personal beliefs about their capability that they could influence students’ performance (Xie et al., 2022). Researchers have found that teaching efficacy affects teachers’ job satisfaction (Collie et al., 2012), classroom management (Tsouloupas et al., 2010), teaching-learning conceptions (Erbas, 2021), and task-centered anxiety levels (Erbas and Ünlü, 2020); additionally, scholars have showcased its important role in helping students to realize their development potential (Pendergast et al., 2011). At present, questions pertaining to which factors can influence teachers’ teaching efficacy have become the focus of global attention. Numerous studies have found that objective antecedents, such as peer support, teaching resources, university types (Chang et al., 2010), and teaching subjects (Riggs and Enochs, 2010) influence teaching efficacy. However, it seems insufficient to consider only the impact of the external factors on teaching efficacy. Enochs and Riggs (1990) have noted that even with given external variables, teachers who lack internal strengths may not believe that they have the capability to teach. Accordingly, studies that explore the effect of the teachers’ personal resources on their teaching efficacy can contribute to theoretical and practical understandings of teaching efficacy.

The Conservation of Resources (COR) theory proposed by Hobfoll (1989) emphasizes resources as the central mechanism to explain the generation and coping of professional stress. The original COR theory defines “resources” as something that are valuable to an individual’s survival and development. Notably, the COR theory roughly classifies resources into four categories: object resources (e.g., shelter), conditional resources (e.g., work), personal characteristic resources (e.g., self-esteem), and energy resources (e.g., time) (Hobfoll, 1989). Hobfoll (1989) argues that humans have always been driven by evolution to acquire, protect, and build on these resources, and to perceive the loss of resources as threats. When pressure creeps in, if individuals are unable to effectively stop the loss of resources and have no opportunity to be compensated in a timely manner, the loss of resources will proceed at an accelerated rate, creating “loss spirals” (Hobfoll, 1989). By contrast, individuals who are able to successfully access beneficial resources when stress signals arise may not only effectively offset resource depletion but may also generate “gain spirals” (Hobfoll, 2001). In Hobfoll (2011) follow-up research, he argued that the COR theory was incomplete because it ignored associations between the resources; in response, he proposed the concept of “resource caravans,” which refer to the accumulation of and linkages between various resources. That is, rather than exist in isolation, individual resources are interrelated and form an array of symbiotic relationships.

Although the COR theory has become an important theory for understanding the driving mechanisms of employee attitudes and behaviors (Westman et al., 2004), it has been challenged by scholars because of its lack of clarity in classifying resources. Brummelhuis and Bakker (2012) reclassified the resources into macro resources (e.g., culture), object resources (e.g., marriage and work), social support (e.g., advice and respect), key resources (e.g., self-efficacy), constructive resources (e.g., knowledge and skills), and energy resources (e.g., emotional and cognitive ability) based on their source and stability. This reclassification has heavily influenced work on the value of resources. Within these resource categories, key resources are placed at a higher level because they are the most stable personality traits (Brummelhuis and Bakker, 2012). However, most of the research on teaching efficacy as a key resource has been conducted without integrating other proposed predictors; accordingly, the mechanisms of the interaction between teachers’ personal resources and teaching efficacy remains elusive. For example, research on the relationship between energy resources and teaching efficacy has been conducted in isolation of other constructive resources (Poulou, 2007), which ignores the interconnectedness of the resources. Moreover, a large number of studies have focused on the loss spirals between resources (Demerouti et al., 2004; McTernan et al., 2016; Deng et al., 2018; Zhou et al., 2019), while ignoring the impact of gain spirals. With the rise of positive psychology, researchers are gradually showing more interest in the advantages of positive resources. For instance, Duyar et al. (2013) found that principals’ leadership and teachers’ collaboration could facilitate teachers’ gain spirals. However, few studies have directly examined how teachers’ personal resources generate such gain spirals from the perspective of teacher assessment literacy professionalism. In response to the gaps in the research identified above, the main aim of our study was to examine the internal mechanisms among teachers’ assessment literacy, psychological capital, professional identity, and teaching efficacy. In doing so, this current study contributes a solid foundation and a new perspective for developing better strategies to improve teachers’ teaching efficacy. It also presents a more comprehensive understanding of the COR theory by demonstrating the positive gaining spirals that exist among teachers’ personal resources and the fact that these resources are not appropriated piecemeal, but rather combined.

### Teachers’ assessment literacy and teaching efficacy

Assessment literacy has become an important component of teacher professionalism and educational practice. Specifically, assessment literacy represents a teacher’s view of education and the utilization of his or her relevant skills and knowledge of assessment to measure students’ achievements in various fields to inform instruction (Stiggins, 1991; Abell and Siegel, 2011; Xu and Brown, 2016; Lam, 2019). More recently, Pastore and Andrade (2019) expanded the concept of teachers’ assessment literacy and revealed that teachers’ assessment literacy should include assessment knowledge, assessment behaviors, and social-emotional competence to implement assessment. According to the COR theory, teachers’ assessment literacy, as a personal resource at the level of knowledge and skills, can help them make accurate inferences about student learning and provide guidance for instruction (Abell and Siegel, 2011), which in turn can increase their teaching efficacy. Conversely, when teachers lack adequate assessment literacy, the reliability and validity of their teaching may be reduced (Carter, 1984; King, 2010), thereby leading them to make incorrect and unwise educational decisions (Xu and Brown, 2017), which has a debilitating effect on their teaching efficacy. Robust and consistent associations have been found between teachers’ assessment literacy and teaching efficacy (Zhang and Burry-Stock, 2003; Eufemia, 2012; Looney et al., 2017). For instance, Zhang and Burry-Stock (2003) in-depth examination suggested that teachers with assessment training tend to have a higher level of efficacy for assessment skills. Using data from 79 teachers in a public school district, Eufemia (2012) found that teacher’s engagement in the formative assessment of their own mathematics teaching was positively related to their self-efficacy in assessment. Looney et al. (2017) similarly indicated that teachers’ confidence in their teaching ability is an important aspect that affects their practices of assessing students in the classroom. On the basis of the above theoretical perspectives and literature findings, the following is hypothesized:

*H1: Secondary school teachers’ level of assessment literacy can positively predict their teaching efficacy.*

### Teachers’ assessment literacy, psychological capital, and teaching efficacy

Psychological capital refers to a state of an individual’s positive development that is representative of their motivational tendencies, which are accumulated through positive psychological constructs, including the following four factors: self-efficacy, hope, optimism, and resilience (Luthans et al., 2007, 2008). Considering the cultural differences between China and the West, Wu et al. (2012) revised the psychological capital measurement scale for localization. In the Chinese cultural context, psychological capital emphasizes the mutual coordination among individuals and the need for people to behave in accordance with social expectations, including two dimensions: transactional psychological capital and interpersonal psychological capital. Transactional psychological capital focuses on individual affairs (e.g., hope and optimism), while interpersonal psychological capital pays attention to the influence of traditional culture and the demands of social life (e.g., gratitude and altruism). According to the COR theory (Brummelhuis and Bakker, 2012), assessment literacy belongs to one of the valuable constructive resources for teachers. Therefore, teachers with better assessment literacy could experience enhanced confidence and satisfaction when they accomplish their teaching tasks and a higher probability of obtaining the psychological resources that they need. Popham (2011) and Luthans et al. (2004) argued that teachers with sufficient assessment literacy are often better able to adapt their instructional plans and reap the rewards of education, and their positive emotions (e.g., confidence) are more likely to be elicited. Accordingly, a higher level of assessment literacy means that a teacher could motivate students to engage in assessment activities and build friendly teacher-student relationships (Xu and Brown, 2017), which contributes to the accumulation of both transactional and interpersonal psychological capital.

Furthermore, the broaden-and-build theory of positive emotions (Fredrickson, 2001) suggests that the accumulation and compounding of teachers’ psychological capital is an important way to construct personal resources. Specifically, teachers with higher levels of psychological capital tend to experience more positive emotions and are able to effectively cope with trials and tribulations at work, thereby expanding their intrinsic motivation and confidence to teach (Isgett and Fredrickson, 2015). Although positive emotions are temporary for teachers, such emotions are also unique in that they can increase resources for teaching efficacy by generating instructional performance (Fredrickson, 2013). Clarence et al. (2021) revealed that teachers with positive emotions effectively engage in creative teaching. Through this process, their contentment and efficacy are more likely to be stimulated. In addition, Heng et al. (2020) found that psychological capital has a greater impact on self-evaluation than certain social resources and is directly related to an individual’s behavioral expectations and self-confidence. On the basis of the abovementioned theoretical perspectives and research findings, the following is hypothesized:

*H2: Secondary school teachers’ level of assessment literacy can indirectly predict their teaching efficacy through the intermediary role of psychological capital.*

### Teachers’ assessment literacy, professional identity, and teaching efficacy

Numerous studies have proposed that professional identity can play a mediating role in many relationships (Andrianto et al., 2018; Chen et al., 2020; Hao et al., 2020). Richter et al. (2021) defined teachers’ professional identity as a multidimensional concept that includes perceptions of teaching tasks, feelings of personal competencies, job satisfaction, and personal belief systems about teaching. Beijaard et al. (2004) highlighted the important role that teachers’ construction of teaching-related practical knowledge plays during the formation of their professional identities. Teachers’ assessment literacy, as practical knowledge essential to the educational process (Fulcher, 2012), is distinctively valuable to their professional identity formation. Pastore and Andrade (2019) found out that the ability to integrate assessment knowledge and practices into pedagogy can help teachers scrutinize their professional identity. In terms of the COR theory, teachers with heightened levels of assessment literacy tend to experience gain spirals through the continued acquisition of resources, which prevents a loss of confidence in their teaching practice and enhances their professional identities.

In addition, Rozati (2017) found that when teachers failed to consider their professional identities in a foreign language setting, it led to their inability to improve their teaching efficacy. Similarly, a study conducted by Esmaili and Dastgoshadeh (2016) with English lecturers at an Iranian university indicated that educators’ knowledge and awareness of their professional identity is critical in enhancing their teaching efficacy. Chen et al. (2020) found that when teachers were loyal to their profession, they were able to ignore unpleasant work situations and become more confident and engaged, thereby increasing their academic self-efficacy. A study on the relationship between teachers’ perceptions of teaching tasks and teaching practices similarly showed that teachers who identified more highly with their profession tended to exhibit higher levels of efficacy (Richter et al., 2021). Combining the aforementioned theories and research results, the following is hypothesized:

*H3: Secondary school teachers’ level of assessment literacy can indirectly predict their teaching efficacy through the intermediary role of professional identity.*

### Psychological capital and professional identity

Previous studies have pointed out that psychological capital can positively impact professional identity. For instance, utilizing 1,009 Chinese nurses, Ren et al. (2021) examined the correlation between psychological capital and professional identity and found that those with high levels of psychological capital have high-level professional identities. Furthermore, Luthans et al. (2008) concluded that psychological capital could positively influence employees’ performance, satisfaction, and commitment. Qiu et al. (2019) found that Chinese doctors’ professional identities could be improved by enhancing the positive resource of psychological capital. Meanwhile, Clarence et al. (2021) found that groups of teachers with positive emotions are able to free themselves from barriers and actively engage in school activities. In such cases, the teachers’ tendency to leave their positions is comparatively weak (Schaufeli and Bakker, 2004), and their professional identity is high. In accordance with these studies, the following is hypothesized:

*H4: Secondary school teachers’ level of assessment literacy can indirectly predict their teaching efficacy through the chain mediating effects of psychological capital and professional identity.*

**FIGURE 1:**
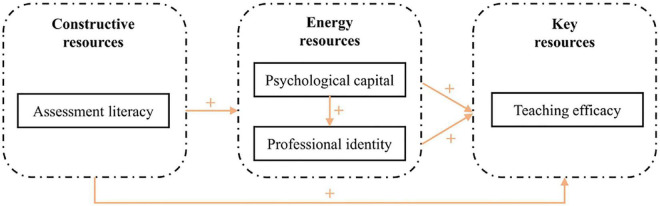
A conservation of resources (COR) conceptual model of the “gain spiral” and “resource caravan” generated by the teacher’s personal resources.

A conceptual model was constructed based on the above four hypotheses to explore the effects of the teachers’ personal resources excluding external object and conditional resources on their teaching efficacy (see Figure 1). This model illustrates the hypothesized mechanism of the link between the teachers’ assessment literacy and their teaching efficacy. The framework includes four variables, which are categorized as constructive resources, energy resources, and key resources according to the resource classification introduced by Brummelhuis and Bakker (2012). Assessment literacy constitutes a constructive resource for teachers. Energy resources include both psychological capital and professional identity, and teaching efficacy is embodied in key resources.

## Materials and methods

### Participants

A combination of cluster and convenience sampling was used to collect the data from teachers in three prefecture-level cities in Henan Province, China using online or paper questionnaires. Between January and March 2022, 433 questionnaires were distributed; after invalid questionnaires were discarded, 351 valid questionnaires were collected (81.6% completion rate). The study was reviewed and approved by the Institutional Review Board of Henan Provincial Key Laboratory of Psychology and Behavior. A consent form was signed by all the participants before they completed the survey. The participants comprised 79 male teachers (22.5%) and 272 female teachers (77.5%); 137 were 20 to 30 years old (39.1%), 145 were 31 to 40 years old (41.3%), 51 were 41 to 50 years old (14.5%), and 18 were over 50 years old (5.1%); 77 had been teaching for less than four years (21.9%), 129 had been teaching for four to 10 years (36.8%), 99 had been teaching for 11 to 20 years (28.2%), 46 had been teaching for more than 20 years (13.1%).

### Materials

#### Teacher assessment literacy scale

We adapted the scale developed by Hudson (2017) to measure the teachers’ levels of assessment literacy. The adapted scale included 33 items along two subscales: teachers’ assessment practices and teachers’ understanding of the assessment. Each subscale comprised five dimensions: assessment design, assessment criteria, use of student participation, setting clear goals, and use of assessment results. An example is as follows: “I design different assessment methods depending on the purpose of the assessment.” The measure was assessed on a 5-point scale (1 = *never*, 5 = *always*) on the assessment practices subscale and a 5-point scale (1 = *completely disagree*, 5 = *completely agree*) on the understanding of the assessment subscale. The confirmatory factor analysis indicated that the fit indices for RMSEA = 0.064, NFI = 0.827, CFI = 0.889, IFI = 0.89. The internal consistency coefficient for this scale was 0.944, and the internal consistency coefficients of the dimensions ranged from 0.738 to 0.844.

#### Teaching efficacy scale

We adopted the Teachers’ Teaching Efficacy Scale originated by Yu et al. (1995) and then revised by Yang (2010) to measure participants’ teaching efficacy. This scale comprises 15 items such as teaching strategies, classroom management, and motivating students. An example is as follows: “I am able to keep the students disciplined in class.” A 5-point scale (1 = *completely inconsistent*, 5 = *completely consistent*) was used. The internal consistency coefficient for this scale was 0.923, and the internal consistency coefficients of the dimensions ranged from 0.801 to 0.839.

#### Psychological capital scale

The Psychological Capital Scale designed by Luthans et al. (2007) and then designed and verified by Wu et al. (2012) was applied. This scale contained 32 items along two dimensions: transactional psychological capital and interpersonal psychological capital. Transactional psychological capital included three factors: hope, optimism, and perseverance. Interpersonal psychological capital included five factors: modesty, gratitude, altruism, emotional intelligence, and self-confidence. An example is as follows: “At the moment, I consider myself quite successful in my career.” Participants rated all items on a 5-point scale (1 = *strongly disagree*, 5 = *strongly agree*). The internal consistency coefficient for the scale was 0.923, and the internal consistency coefficients of the dimensions ranged from 0.886 to 0.942.

#### Teacher professional identity scale

The 18-item Teacher Professional Identity Scale conducted by Wei (2008) was adopted, including four dimensions: role values, professional values, professional affiliation, and professional behavior tendencies. An example is as follows: “I am proud to be a teacher.” A 5-point scale (1 = *completely inconsistent*, 5 = *completely consistent*) was used. The internal consistency coefficient for this scale was 0.956, and the internal consistency coefficients of the dimensions ranged from 0.729 to 0.854.

### Data analysis

IBM SPSS25.0 was used to perform the preliminary data processing, generate descriptive statistics, and conduct reliability, correlation, and regression analyses. The chain mediating role of the teachers’ psychological capital and professional identity in the relationship between assessment literacy and teaching efficacy was checked using Model 6 of the PROCESS macro.^1^ The bias-corrected percentile bootstrap method was used to test the significance of the mediating role. A 95% confidence interval was considered statistically significant if it did not contain a value of zero (Erceg-Hurn and Mirosevich, 2008). Furthermore, prior to analyzing the data, we applied Harman’s one-factor test to verify the common method variance of the variables (Podsakoff et al., 2003).

## Results

### Common method bias test

This study used Harman’s one-factor method to put the teachers’ assessment literacy, psychological capital, professional identity, and teaching efficacy items into the exploratory factor analysis. The maximum factor can only explain a variance of 30.84%, which was less than the 40% threshold, thereby indicating that no significant common method variance existed and that the relationship between the variables is credible.

### Descriptive analyses

The means, SD, and correlation coefficients for the participating teachers’ assessment literacy, teaching efficacy, psychological capital, and professional identity are shown in Table 1. Teachers’ assessment literacy is positively correlated with teaching efficacy (*r* = 0.581, *p* < 0.01), psychological capital (*r* = 0.603, *p* < 0.01), and professional identity (*r* = 0.591, *p* < 0.01). Teaching efficacy is positively correlated with psychological capital (*r* = 0.652, *p* < 0.01) and professional identity (*r* = 0.675, *p* < 0.01). There is a significant positive correlation between psychological capital and professional identity (*r* = 0.682, *p* < 0.01). The results indicate that all variables are significantly positively correlated.

**TABLE 1:** Descriptive statistics and correlation matrix variables.

Variables	*M*	*SD*	1	2	3	4
1. Teachers’ assessment literacy	3.917	0.475	1			
2. Teaching efficacy	4.102	0.475	0.581**	1		
3. Psychological capital	4.103	0.469	0.603**	0.652**	1	
4. Professional identity	4.228	0.473	0.591**	0.675**	0.682**	1

**Significant correlation at the 0.01 level (two-tailed test), *p* < 0.01.

### Chain mediation model analysis

Teachers’ assessment literacy, psychological capital, professional identity, and teaching efficacy are significantly correlated, which meets the statistical requirements for further analysis of the mediating effect of the teachers’ assessment literacy and teaching efficacy (Wen and Ye, 2014). We used Model 6 in the SPSS macro program (Hayes, 2012) to analyze the mediating effects of psychological capital and professional identity between the teachers’ assessment literacy and teaching efficacy, while controlling for variables of gender, age, and teaching experience.

The regression analysis results are shown in Table 2. The results indicate that the teachers’ assessment literacy has a significant positive predictive effect on the teaching efficacy (β = 0.590, *p* < 0.001). When psychological capital and professional identity are introduced into the regression analysis, the teachers’ assessment literacy is a significant positive predictor of psychological capital (β = 0.609, *p* < 0.001) and professional identity (β = 0.280, *p* < 0.001). Moreover, psychological capital significantly predicts professional identity (β = 0.515, *p* < 0.001) and teaching efficacy (β = 0.284, *p* < 0.001). Professional identity significantly predicts teaching efficacy (β = 0.365, *p* < 0.001). Meanwhile, the direct effect of the teachers’ assessment literacy on teaching efficacy is decreased (β = 0.201, *p* < 0.001). These results suggest that the independent intermediary effects of psychological capital and professional identity, together with the chain intermediary effect of psychological capital → professional identity, are significant in the influence of the teachers’ assessment literacy on teaching efficacy. Thus, Hypotheses 1–4 are all confirmed.

**TABLE 2:** Regression analysis of the relationship between teacher assessment literacy and teaching efficacy.

Regression equation result variable	Predictor variable	Index of fit			Significance	
		*R*	*R* ^2^	*F*	β	*t*
Teaching efficacy		0.588	0.339	45.807***		
	Gender				–0.095	−2.098*
	Age				–0.008	–0.103
	Teaching experience				–0.007	–0.098
	Teacher assessment literacy				0.590	13.377***
Psychological capital		0.612	0.368	51.914***		
	Gender				–0.070	–1.581
	Age				0.116	1.575
	Teaching experience				–0.073	–0.991
	Teacher assessment literacy				0.609	14.101***
Professional identity		0.719	0.510	73.837***		
	Gender				–0.003	–0.086
	Age				–0.030	–0.461
	Teaching experience				0.003	0.046
	Psychological capital				0.515	10.877***
	Teacher assessment literacy				0.280	5.864***
Teaching efficacy		0.741	0.542	70.001***		
	Gender				–0.061	–1.606
	Age				–0.052	–0.819
	Teaching experience				0.026	0.414
	Professional identity				0.365	7.006***
	Psychological capital				0.284	5.362***
	Teacher assessment literacy				0.201	4.162***

**FIGURE 2:**
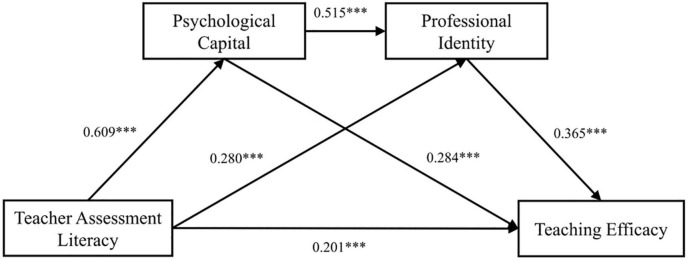
Chain mediation model. ***p < 0.001.

Table 3 shows the mediating effect values of psychological capital and professional identity on the relationship between teachers’ assessment literacy and teaching efficacy; the chain mediating model is shown in Figure 2. The results indicate that the total indirect effect accounts for 67.01%, and the 95% confidence interval does not contain zero (0.303, 0.489). Teacher Assessment Literacy → Psychological Capital → Teaching Efficacy mediating effect is significant (β = 0.173), accounting for 29.73%. Teacher Assessment Literacy → Professional Identity → Teaching Efficacy mediating effect is significant (β = 0.103), accounting for 17.70%. The chain multiple mediation effect of Teacher Assessment Literacy → Psychological Capital → Professional Identity → Teaching Efficacy is significant (β = 0.113), accounting for 19.42%. Hypotheses 2–4 are thus confirmed once again.

**TABLE 3:** Multiple mediated analysis between the teacher variables.

	Effect	Boot SE	Bootstrap 95% CI		Effect ratio
			Low	High	
Total effect	0.582	0.044	0.496	0.667	100%
Direct effect	0.192	0.048	0.098	0.286	32.99%
Total indirect effect	0.390	0.048	0.303	0.489	67.01%
Path 1: Teacher assessment literacy → Psychological capital → Teaching efficacy	0.173	0.043	0.095	0.265	29.73%
Path 2: Teacher assessment literacy → Professional identity →Teaching efficacy	0.103	0.032	0.048	0.174	17.70%
Path 3: Teacher assessment literacy→ Psychological capital → Professional identity → Teaching efficacy	0.113	0.023	0.071	0.162	19.42%

## Discussion

This study constructed a complete theoretical framework around the classification and gain spiral of resources and explores the influence of teachers’ constructive and energy resources on teaching efficacy. The findings offer novel evidence that teachers’ assessment literacy, as a constructive resource, affects their teaching efficacy directly and indirectly through the intermediary of their psychological capital and professional identity. The current study extends the existing research in three important ways.

First, we found that teachers’ assessment literacy significantly and positively predicted their teaching efficacy, which validated Hypothesis 1. This result is consistent with a study by Zhang and Burry-Stock (2003), which reported that teachers with higher levels of assessment literacy have a greater sense of efficacy in their teaching. This finding also supports the research of Eufemia (2012) and Looney et al. (2017), which states that professional competence strengthens teachers’ perceptions of the value of their work, thus increasing their motivation and sense of efficacy in accomplishing teaching tasks. It can also be demonstrated that a teacher’s level of professional development is an important motivating factor in enhancing their teaching confidence in the Chinese context. Furthermore, exploring the gain spirals that result from the impact of teacher assessment literacy on teaching efficacy is particularly important, as it highlights the idea that teachers with solid resource reserves have a greater probability of experiencing the effects of resource enrichment (Hobfoll, 2002). That is, the acquisition and accumulation of assessment literacy resources can be considered a pivotal driver that initiates and maintains teachers’ teaching efficacy (Carter, 1984; King, 2010; Abell and Siegel, 2011). This finding enriches the current research on gain spirals. In this regard, teachers with higher assessment literacy are more likely to positively influence students’ learning than teachers who lack assessment knowledge and skills training. Because of the additional confidence they have in their teaching capabilities and the competence to infer the validity and reliability of their students’ learning (Deluca et al., 2013; Xu and Brown, 2017), which in turn may promote the development of teaching efficacy resources. Since resource conservation is a primary concern, the internal process that triggers the spiral of resource gain within teachers is an important part of building and maintaining the “resource caravans.” Teachers create the primary motivation that necessarily supports this caravan when they strive to acquire, retrain, and protect personal resources for themselves (Hobfoll, 2014). Through a review of previous research on the COR theory (Hobfoll and Lilly, 1993), we devised a more coherent picture of how teachers’ assessment knowledge and skills resources produce teaching efficacy gain spirals. This study complements the theoretical gap in the field of teacher professional development regarding the resource caravans generated by teachers’ constructive resources.

Second, the present study showed that psychological capital and professional identity individually and continuously mediated the relationship between teachers’ assessment literacy and teaching efficacy by testing for mediating effects. This verified Hypotheses 2–4. Notably, this finding is supported by similar studies (Beijaard et al., 2004; Fulcher, 2012; Qiu et al., 2019; Chen et al., 2020; Clarence et al., 2021). The total indirect effect accounted for 67.01% of the total effect, which was greater than the direct effect in the total effect (32.99%). This indicates that teachers with a positive psychological state or a higher level of identification with their profession are able to adopt constructive coping strategies, leading to increased optimism, self-affirmation and professional belonging, which ultimately enhance their teaching efficacy (Esmaili and Dastgoshadeh, 2016; Rozati, 2017; Heng et al., 2020). More importantly, the examination of the chain mediation effects of teachers’ psychological capital and professional identity yielded solid evidence that is consistent with the notion that an individual’s resources do not exist in isolation but are clustered together (Hobfoll, 2002). The self-perpetuating, complex, and dynamic motivational processes that take place among resources in the teachers’ resource caravans have also been validated (Salanova et al., 2010). This finding further verifies and explains the broaden-and-build theory of positive emotions (Fredrickson, 2001; Isgett and Fredrickson, 2015). The most influential explanation for the mechanism behind these relationships posits that teachers’ assessment literacy boosts teaching efficacy by increasing the energy resources critical to teaching success. More specifically, once teachers’ assessment literacy is well-developed, they will feel more confident in their assessment abilities and respond to the challenges in their teaching practice with positive emotions (Luthans et al., 2004; Popham, 2011). Simultaneously, teachers who have positive psychological capital will be more satisfied with their work, and show more emotional commitment, continuity and normativeness (Larson and Luthans, 2006), which will allow them to maximize their key resource (i.e., teaching efficacy). Overall, this study provides empirical support for the potential mechanisms of resource caravans and contributes new insights into the dynamic interactions between resources highlighted by the COR theory in the professional field of teaching.

Finally, the present study’s results have several important practical implications. On the one hand, given the role of teachers’ assessment literacy in predicting teaching efficacy, teachers may invest more time and energy in training on assessment knowledge and skills to help them stay up-to-date with the knowledge on educational assessment. At the same time, it is beneficial to guide teachers to link the assessment principles and strategies with the relevant assessment practices to increase their assessment experiences in teaching (Abell and Siegel, 2011), thereby enhancing their assessment literacy and teaching confidence, which increase their personal resource storage. It is important to note that assessment trainers could examine teachers’ current assessment experiences, actively seek out a variety of available resources for assessment training, and provide guidance to teachers in all phases of assessment development and use during teacher assessment training (Yan et al., 2018). On the other hand, from the perspective of the role of teachers’ psychological capital and professional identity, educational administrators could enhance their support and care for teachers to strengthen the protection of positive resources, such as optimism, resilience, and a sense of belonging. Teachers could increase their energy resources by maintaining positive psychological states and professional identities, thereby preventing negative effects on their teaching efficacy when faced with job challenges. It is worth noting that, compared with unstable external social supports, a combination of factors such as assessment literacy, psychological capital, and professional identity have helped teachers survive. For instance, Bellibas and Liu (2017) found that teachers felt a higher sense of efficacy when they received additional professional development through a training program combined with the principal’s instructional leadership. In light of this, to enhance teaching efficacy, it is important to consider not only external factors, such as help from others and performance feedback, but also, and more importantly, factors such as teachers’ cultivation and development of their constructive resources and energy resources. All in all, schools should provide teachers with a shared marketplace of resources and facilitate the flow of positive resources through management mechanisms, thereby creating opportunities for teachers to acquire and grow resources (Chen et al., 2015; Hobfoll et al., 2018). In this way, teachers can increase their ability to resist stress through gain spirals among resources, ultimately facilitating the accumulation of key resources.

## Conclusion

To conclude, based on the COR theory, this study explored the influence mechanism of teachers’ assessment literacy on teaching efficacy and tested the mediation roles of psychological capital and professional identity. Identification of the chain-based multi-mediating role reveals new pathways to consider the impact of the teachers’ assessment literacy on teaching efficacy. The findings suggest that teachers’ assessment literacy is an important antecedent for predicting psychological capital, professional identity, and teaching efficacy. Teachers’ assessment literacy can influence their teaching efficacy directly or indirectly through psychological capital and professional identity. The present study simultaneously underscores that rich individual constructive resources and energy resources facilitate positive gaining spirals in key resources; these findings validate and enrich existing research on the COR theory. In addition, the relationships among the multiple personal resources teachers possess are not isolated, separated, and fragmented from one another, but rather interconnected and interactive, ultimately forming a web-like array of resources; notably, this finding extends the COR theory on resource caravans. Taken together, the findings of this study provide information on how to improve teachers’ assessment literacy to enhance psychological capital and professional identity, thereby snowballing their sense of teaching efficacy.

### Limitations and future directions

This study shows some shortcomings. (1) Due to the pandemic conditions, the sample of this study is relatively small and focused on the Henan Province, which cannot reflect the overall situation of Chinese teachers. Follow-up research should be conducted in a wider area of China to increase the representativeness and diversity of the sample. (2) This study features only cross-sectional data; thus, solid conclusions cannot be drawn. To improve the reliability and validity of the present results, follow-up longitudinal study could be considered. (3) The influence of the external resources on teaching efficacy and the uncertainty of whether external resources can affect teaching efficacy through their internal resources need to be resolved. (4) Given that teaching efficacy is a key resource for teachers’ professional development, it is unclear what role it plays in promoting teachers’ assessment literacy, psychological capital, and professional identity. The contribution of teaching efficacy to teachers’ constructive resources and energy resources could be considered in future research. In short, our results advance the evidence illustrating the core role of the teachers’ assessment literacy, psychological capital, and professional identity in teaching efficacy; however, follow-up studies are required to resolve the abovementioned issues.

## Data availability statement

The original contributions presented in this study are included in the article/Supplementary material, further inquiries can be directed to the corresponding author.

## Ethics statement

The studies involving human participants were reviewed and approved by Institutional Review Board of Henan Provincial Key Laboratory of Psychology and Behavior. The patients/participants provided their written informed consent to participate in this study.

## Author contributions

PZ and HW designed the study and reviewed and revised the manuscript. WS drafted the manuscript and coordinated the data collection. YZ and TL analyzed the data and revised the manuscript. All authors contributed to the article and approved the submitted version.

## Footnotes

1
http://www.afhayes.com
