# Supplementary material for: Teachers’ assessment literacy improves teaching efficacy: A view from conservation of resources theory
Source: Front Psychol. 2022 Oct 28;13:1007830. doi: 10.3389/fpsyg.2022.1007830 (PMC9649984; doi:10.3389/fpsyg.2022.1007830)
Supplement: Supplementary file 1 [file Data_Sheet_1.PDF]

serial number	gender	age	teaching experience	Aa1	Aa2	Aa3	Aa4	Aa5
1	2	2	3	3	4	4	3	3
2	2	2	3	2	4	1	3	2
3	1	4	4	1	2	4	5	4
4	2	3	3	1	2	4	5	4
5	2	2	3	1	2	4	5	4
6	2	3	3	1	2	4	3	3
7	2	2	3	1	2	4	5	3
8	1	4	4	3	3	3	3	3
9	1	2	3	4	3	4	4	5
10	2	2	2	2	3	2	3	3
11	2	1	1	4	4	4	5	3
12	1	3	3	4	5	4	3	2
13	1	2	3	4	4	3	3	3
14	2	2	3	4	4	4	4	4
15	2	2	3	4	4	4	4	4
16	2	3	4	3	4	4	4	3
17	1	2	3	4	4	4	2	3
18	2	2	3	4	5	3	4	5
19	1	2	3	4	4	3	5	3
20	2	2	3	4	5	4	3	3
21	2	2	3	4	4	4	4	3
22	2	3	4	4	3	5	3	5
23	2	2	2	3	3	4	4	3
24	2	1	1	2	4	4	4	4
25	2	1	1	2	4	4	4	4
26	2	2	3	5	5	3	5	4
27	2	2	3	5	5	3	5	4
28	2	2	3	4	4	4	3	4
29	2	2	3	4	3	4	4	4
30	2	2	3	2	3	4	4	3
31	2	1	2	4	2	3	2	2
32	1	3	4	3	3	4	3	3
33	2	3	4	3	5	5	5	5
34	1	2	3	4	5	3	3	3
35	2	2	3	4	4	3	4	3
36	2	2	3	3	3	4	3	3
37	1	2	3	3	3	2	3	3
38	2	2	3	3	3	4	3	4
39	2	2	3	4	4	4	3	2
40	2	2	3	5	5	3	3	3
41	2	1	1	4	4	3	3	4
42	2	2	3	2	3	2	4	2
43	1	3	3	4	4	3	3	2
44	1	2	3	4	3	4	4	3
45	2	2	3	2	3	3	2	1
46	1	2	3	5	4	5	5	4
47	1	3	3	4	4	3	4	4
48	1	3	4	3	4	4	3	4
49	1	3	4	4	4	3	4	4
50	1	2	3	4	4	5	4	4
51	2	2	2	3	4	3	3	3

52	2	2	4	4	4	4	4	4
53	2	2	2	4	3	4	4	5
54	1	1	2	4	3	4	4	4
55	2	2	2	4	4	5	5	4
56	2	2	3	4	2	3	2	4
57	2	1	3	4	4	4	3	3
58	1	2	4	3	4	3	4	4
59	2	3	4	3	4	4	4	4
60	2	3	4	3	3	3	3	2
61	2	2	2	4	4	4	2	2
62	2	2	2	4	4	3	3	4
63	2	1	1	2	2	3	2	2
64	1	3	3	2	2	2	2	4
65	1	1	2	3	4	2	2	4
66	2	3	4	4	5	3	5	5
67	2	1	1	4	4	4	4	4
68	2	1	1	4	5	5	5	5
69	2	1	2	5	4	4	4	4
70	2	1	2	3	4	4	4	2
71	2	1	1	3	4	3	3	3
72	2	3	3	4	5	4	4	4
73	2	2	2	4	5	4	4	5
74	2	2	2	3	3	3	3	3
75	2	1	2	3	4	4	4	4
76	2	2	2	3	3	2	2	2
77	2	1	1	4	4	3	3	4
78	2	2	2	3	4	4	3	4
79	1	3	3	3	4	4	3	4
80	2	1	2	3	4	3	3	4
81	2	1	1	5	4	5	5	5
82	1	2	1	4	3	2	3	4
83	2	3	4	4	4	4	4	4
84	2	1	1	4	4	4	4	3
85	2	1	2	5	5	5	5	4
86	2	1	1	4	4	4	4	4
87	2	1	1	4	4	4	4	4
88	2	2	3	4	4	4	3	3
89	1	1	1	4	4	4	4	5
90	2	2	2	3	3	3	3	3
91	1	3	3	5	4	4	5	5
92	1	3	3	4	4	3	4	4
93	2	2	3	3	5	4	3	3
94	2	1	2	3	5	4	3	3
95	2	1	2	3	4	3	3	4
96	2	1	2	3	4	3	3	4
97	2	1	2	5	5	4	4	3
98	2	2	3	4	4	4	3	3
99	2	2	2	3	3	4	4	4
100	2	2	2	4	4	3	3	4
101	2	1	2	4	4	4	4	3
102	2	2	2	4	4	4	4	4
103	2	2	2	4	4	4	4	4
104	2	2	3	5	4	4	4	4
105	2	3	4	3	4	4	3	3

106	2	2	2	3	3	3	3	3
107	2	1	2	4	3	3	3	4
108	2	1	2	4	5	4	4	4
109	2	2	2	3	3	4	4	3
110	2	2	2	2	3	3	3	4
111	1	1	1	3	3	3	3	3
112	1	1	2	2	3	3	4	4
113	2	2	2	4	5	4	2	3
114	2	2	3	4	5	4	2	3
115	2	2	2	5	5	5	5	5
116	2	3	3	3	2	4	3	2
117	2	2	2	4	5	4	2	3
118	2	2	2	3	3	3	3	3
119	2	2	3	4	4	4	2	2
120	2	3	3	4	3	2	2	3
121	2	1	2	4	4	3	2	3
122	2	3	3	4	4	5	5	5
123	2	1	1	4	4	3	4	4
124	2	2	3	5	5	5	5	4
125	2	3	4	3	4	5	2	3
126	2	2	3	5	5	4	4	5
127	2	3	3	5	5	5	5	5
128	2	1	2	3	4	2	3	3
129	2	2	3	3	4	3	4	2
130	2	3	3	4	4	4	5	4
131	2	2	3	4	5	4	4	2
132	1	3	4	4	3	4	2	4
133	2	3	4	2	3	2	4	2
134	1	2	3	5	5	5	5	5
135	1	1	2	4	4	4	4	3
136	2	1	1	5	5	5	5	5
137	2	1	1	4	4	4	4	4
138	1	3	3	4	5	4	5	5
139	1	4	4	3	4	2	4	3
140	1	2	2	2	3	4	3	4
141	1	1	1	4	3	3	4	3
142	2	2	3	3	3	3	3	3
143	2	2	3	2	3	3	3	3
144	2	4	4	4	4	3	4	3
145	2	2	2	3	4	3	4	4
146	1	4	4	3	3	2	3	2
147	2	3	3	4	4	3	4	3
148	2	3	3	4	4	3	4	3
149	1	3	3	4	5	4	3	3
150	2	2	3	5	5	4	4	3
151	2	1	1	2	3	4	4	4
152	1	2	3	3	3	3	3	3
153	1	3	4	3	3	3	2	3
154	2	2	3	5	4	4	4	4
155	2	1	1	3	4	3	3	3
156	1	2	3	4	4	3	2	3
157	2	1	1	4	2	4	3	4
158	2	2	2	4	3	4	4	4
159	2	1	2	3	3	3	3	3

160	2	3	3	4	1	4	3	3
161	2	2	3	3	3	4	4	3
162	1	2	3	3	4	3	2	2
163	2	2	2	3	4	4	4	4
164	2	1	2	4	3	4	4	3
165	2	1	2	3	5	4	3	3
166	2	1	2	4	4	4	4	4
167	1	1	1	3	3	2	3	4
168	2	1	2	4	4	4	4	4
169	2	1	2	3	3	4	3	4
170	2	1	2	3	4	2	4	3
171	2	1	1	3	3	3	2	3
172	2	2	2	4	4	5	4	5
173	1	1	1	2	2	4	3	4
174	1	2	2	2	2	4	3	4
175	2	2	2	2	3	3	3	3
176	1	2	2	4	4	5	4	4
177	2	2	2	3	3	3	3	2
178	2	2	2	4	4	4	4	4
179	2	2	4	2	3	2	3	3
180	2	1	1	3	3	3	4	4
181	2	1	1	3	3	3	4	4
182	2	1	2	3	4	4	4	4
183	2	1	2	4	4	4	5	4
184	1	1	1	4	4	4	4	4
185	2	1	2	3	4	4	4	3
186	2	1	2	3	4	4	3	4
187	2	1	3	3	3	3	3	3
188	2	1	2	3	3	3	3	3
189	2	2	2	3	3	2	3	4
190	2	1	2	3	4	4	3	4
191	2	1	2	3	4	4	3	4
192	2	1	2	3	4	5	3	3
193	2	2	2	2	3	4	4	4
194	2	1	2	3	3	3	3	3
195	2	1	2	3	3	4	3	3
196	1	4	4	5	5	5	5	5
197	2	2	2	3	3	4	2	2
198	2	1	2	4	3	3	3	2
199	1	1	2	3	4	5	3	4
200	2	1	1	4	3	4	4	4
201	2	1	2	4	4	3	3	2
202	2	1	1	3	3	3	4	3
203	2	1	1	3	3	4	4	4
204	2	2	2	3	4	5	4	4
205	2	1	1	5	5	5	5	5
206	2	2	3	2	3	3	2	3
207	2	2	2	4	3	2	3	4
208	2	1	2	5	5	3	5	5
209	2	1	1	3	3	3	4	4
210	2	1	1	3	3	3	2	3
211	1	1	1	3	3	3	4	4
212	2	1	1	4	5	5	4	5
213	2	1	1	2	2	3	4	5

214	2	1	1	4	4	4	4	4
215	2	1	1	5	5	5	4	5
216	2	1	1	4	4	4	4	4
217	2	2	3	3	3	3	3	3
218	2	1	2	4	3	4	3	4
219	2	1	2	4	4	4	4	4
220	2	2	2	5	5	5	4	5
221	2	1	2	4	5	3	4	4
222	2	2	2	4	4	4	4	4
223	2	1	2	4	4	3	4	3
224	2	1	1	3	3	3	4	4
225	2	1	1	3	3	3	4	4
226	2	2	3	4	3	4	4	3
227	1	1	1	4	4	5	4	5
228	2	2	2	5	5	5	5	5
229	2	1	2	2	2	3	4	4
230	2	1	3	3	3	2	4	3
231	2	1	1	4	4	4	4	3
232	2	2	2	4	4	4	4	4
233	2	1	1	3	2	3	4	3
234	2	1	1	3	4	3	2	3
235	2	1	1	3	4	4	2	3
236	2	1	2	2	3	4	2	3
237	2	1	1	3	3	3	3	3
238	2	1	2	4	3	3	3	4
239	2	1	2	3	4	3	4	4
240	2	3	3	4	4	4	4	4
241	2	2	2	4	4	4	4	4
242	1	1	1	4	4	4	4	4
243	2	1	2	3	4	4	4	4
244	2	1	1	3	4	3	3	3
245	2	1	2	3	3	3	3	3
246	1	1	1	5	5	4	5	4
247	2	2	2	4	4	4	2	2
248	2	1	1	1	2	3	1	3
249	2	1	1	4	4	3	5	3
250	2	1	1	3	4	4	4	3
251	2	2	3	3	3	3	4	3
252	2	1	2	4	5	3	4	3
253	2	1	1	3	3	3	3	3
254	2	1	2	2	3	4	5	1
255	2	1	1	4	4	4	4	3
256	2	2	2	3	3	4	4	4
257	2	1	2	3	4	4	3	3
258	2	1	2	4	5	5	4	4
259	2	1	1	4	4	4	2	2
260	2	1	1	4	4	4	2	5
261	2	1	1	4	4	4	5	5
262	2	1	2	5	5	5	5	5
263	2	2	2	4	4	4	4	3
264	2	2	2	4	5	5	5	5
265	2	1	1	2	3	3	3	2
266	1	1	2	3	4	4	3	3
267	1	2	2	4	5	5	5	5

268	2	1	2	5	5	5	4	5
269	2	1	1	4	5	4	5	4
270	2	4	4	3	3	2	3	3
271	1	1	2	4	3	2	3	4
272	2	3	4	4	4	4	3	5
273	2	2	3	3	3	3	3	3
274	2	2	2	3	4	3	3	4
275	2	2	3	4	4	4	4	4
276	2	1	2	4	4	4	3	3
277	2	2	3	4	4	4	4	4
278	2	1	1	3	3	3	3	3
279	2	4	4	5	5	4	5	5
280	1	4	4	2	3	1	3	3
281	1	4	4	4	4	4	4	4
282	2	4	4	4	4	4	4	4
283	2	2	3	4	4	4	4	4
284	2	4	4	4	4	4	4	4
285	2	1	2	3	4	3	4	4
286	2	4	4	4	4	4	4	4
287	2	2	3	4	4	4	4	4
288	2	4	4	4	4	4	4	4
289	1	3	4	2	3	2	3	2
290	2	3	3	2	1	2	2	3
291	1	3	3	2	2	3	2	3
292	1	4	4	4	3	4	4	2
293	2	1	2	3	3	3	4	3
294	2	1	1	4	4	4	4	5
295	2	2	2	4	4	4	4	4
296	2	1	2	3	4	3	4	4
297	2	3	4	4	4	5	3	2
298	2	1	1	3	3	3	4	4
299	1	3	4	3	3	3	3	3
300	1	2	3	3	3	3	3	3
301	2	1	1	4	4	4	4	4
302	2	1	1	4	3	4	3	3
303	2	2	3	2	3	2	3	2
304	1	3	4	4	4	4	3	3
305	1	2	3	4	3	4	4	3
306	2	1	1	3	4	4	5	5
307	1	2	2	4	2	4	3	3
308	1	2	3	4	4	4	4	4
309	1	1	1	4	4	4	4	4
310	1	4	4	4	4	5	4	4
311	1	2	2	3	3	4	3	3
312	1	3	4	4	4	4	4	4
313	2	2	2	5	5	3	3	2
314	1	2	2	5	4	4	4	4
315	1	2	2	4	4	4	3	4
316	1	2	2	3	3	4	3	3
317	2	2	3	2	3	3	3	3
318	1	2	3	3	3	3	3	3
319	2	2	3	5	5	5	5	5
320	2	2	2	4	4	4	4	4
321	2	2	3	4	4	4	2	5

322	2	2	2	4	4	4	4	4
323	2	2	1	5	5	4	5	3
324	2	2	2	5	5	5	5	5
325	1	2	2	5	5	5	5	5
326	1	2	2	3	3	4	4	4
327	2	3	3	5	3	2	3	4
328	2	3	4	4	4	4	4	4
329	2	2	2	4	4	4	4	4
330	2	1	1	3	3	4	4	4
331	2	1	1	3	3	4	4	3
332	2	3	4	5	5	3	3	3
333	2	1	1	5	5	5	5	4
334	2	2	1	3	4	4	3	5
335	2	3	3	3	2	5	3	3
336	2	2	2	4	4	3	4	4
337	1	3	4	3	3	4	2	4
338	1	2	3	3	3	3	3	3
339	2	2	3	4	4	2	4	5
340	2	3	4	3	4	3	4	3
341	2	2	3	3	3	3	3	3
342	1	2	3	3	3	2	2	3
343	2	2	2	5	4	4	4	5
344	2	2	2	4	4	4	3	3
345	2	3	2	5	2	3	2	3
346	2	1	1	4	4	3	4	4
347	2	2	2	5	5	5	5	5
348	2	4	4	4	4	4	3	3
349	2	2	1	4	4	5	3	3
350	2	2	1	5	4	4	5	5
351	2	4	4	3	3	3	3	3

Aa6	Aa7	Aa8	Aa9	Aa10	Aa11	Aa12	Aa13	Aa14
4	4	2	2	2	4	3	2	3
1	2	1	3	1	5	4	2	3
3	3	4	5	5	4	3	4	5
3	3	4	5	5	4	4	4	5
3	3	4	5	5	4	5	4	5
4	4	5	4	3	4	5	5	4
3	4	5	4	3	4	5	4	3
2	3	3	4	4	4	3	3	3
4	4	4	3	3	4	3	3	4
3	3	4	4	4	4	4	4	4
5	4	2	5	5	5	3	5	5
5	4	5	5	5	4	3	5	3
4	3	3	3	4	4	3	4	3
4	5	4	5	5	4	5	4	5
4	5	4	5	5	4	5	4	5
2	3	4	4	4	3	3	4	4
3	4	2	3	4	3	4	4	4
4	3	4	5	5	4	3	4	5
5	3	4	5	3	4	5	5	4
3	4	3	4	5	4	4	3	4
3	4	2	4	5	4	4	4	4
3	4	4	3	4	4	3	4	4
4	4	3	5	5	3	5	5	5
3	4	4	4	5	4	4	5	2
3	4	4	5	4	4	4	5	3
4	4	4	5	5	4	4	4	5
4	4	4	5	5	4	4	4	5
4	4	4	4	4	4	4	4	4
4	5	3	4	5	5	4	3	4
3	4	5	4	3	2	3	4	5
3	3	3	4	2	4	3	3	3
2	4	3	3	4	4	5	4	3
4	5	5	4	4	4	5	5	5
4	2	4	3	5	5	4	3	4
2	4	4	4	4	3	4	4	4
2	4	3	3	4	2	3	4	5
4	3	4	3	3	3	3	3	3
4	3	4	4	4	4	3	3	4
2	3	3	3	4	5	5	5	2
2	4	4	4	5	3	5	5	5
3	4	2	3	4	3	3	4	3
4	2	1	3	4	1	3	4	1
4	4	5	5	5	4	4	4	4
4	5	3	3	5	5	5	4	3
2	3	4	4	3	3	2	2	3
4	4	4	5	4	4	4	4	5
4	4	3	5	4	4	4	5	4
3	3	4	5	5	3	4	3	5
3	5	4	3	4	4	4	3	4
4	3	4	3	4	4	4	5	3
3	4	4	4	4	3	3	4	3

4	4	4	4	4	4	4	4	4
4	3	4	3	4	4	4	3	4
3	3	3	5	4	4	4	3	4
3	3	3	3	4	5	5	5	4
2	4	3	4	3	4	3	2	3
4	5	5	4	3	3	4	4	5
3	3	4	3	4	3	4	3	4
5	4	5	4	5	4	5	5	4
4	2	4	5	3	3	3	1	4
4	5	2	3	4	3	4	4	4
4	4	4	4	4	4	4	4	4
2	3	2	2	2	2	2	2	3
4	2	2	5	4	3	4	4	4
4	3	3	4	2	2	1	5	4
5	5	4	5	5	5	5	5	5
4	4	4	4	4	4	4	4	4
5	4	5	5	5	5	5	5	5
4	5	4	5	5	5	5	5	5
4	4	2	5	5	5	5	5	5
3	3	3	4	3	3	3	3	3
4	4	4	4	4	3	4	4	5
4	4	4	5	5	4	4	4	5
4	4	4	4	4	4	3	3	3
3	4	2	5	4	2	2	4	5
3	3	3	3	3	4	4	4	3
5	4	5	5	4	4	4	5	5
4	3	3	4	4	3	3	4	4
4	4	3	3	4	4	3	3	4
3	4	2	4	4	4	4	3	5
5	4	2	4	5	3	4	5	4
2	3	3	3	2	3	4	3	4
2	5	4	5	5	5	5	4	5
4	5	4	5	4	4	4	4	4
4	5	4	5	5	5	4	4	4
4	3	4	3	4	3	4	4	4
4	4	4	4	4	4	4	4	4
4	4	4	4	4	4	4	4	4
5	4	4	4	4	4	4	4	4
3	3	3	3	3	3	3	3	3
5	5	5	5	5	5	5	5	4
4	4	3	3	4	4	3	3	3
5	5	4	4	4	4	3	4	5
5	5	4	4	4	4	3	4	4
4	4	4	4	4	4	4	4	4
4	4	4	4	4	4	4	4	4
5	4	4	5	4	4	4	5	4
5	5	3	5	4	4	5	5	2
4	4	3	4	4	4	4	4	3
4	4	2	4	4	3	3	4	3
4	4	4	4	4	4	4	4	4
4	4	5	5	5	4	4	4	4
3	4	3	4	4	4	4	4	4
3	4	3	4	4	4	4	4	4
3	4	3	4	4	4	5	5	4

3	4	3	4	4	4	3	4	3
3	4	3	3	3	4	3	4	3
4	5	5	5	5	4	4	5	4
3	4	4	4	3	4	3	4	3
4	4	2	3	5	3	3	4	4
3	3	3	3	3	2	3	4	2
3	2	3	3	4	3	3	4	3
4	4	2	3	4	4	3	4	4
4	4	2	3	4	4	3	4	4
5	5	5	5	5	5	5	5	5
2	3	3	2	2	3	2	4	4
4	4	2	3	4	4	3	4	4
3	3	3	3	4	3	4	4	3
3	4	4	4	4	4	4	4	4
1	4	4	4	4	4	4	4	4
3	4	3	4	4	3	4	4	3
2	4	5	5	5	4	5	5	5
3	4	4	4	4	4	4	4	4
4	4	4	4	4	4	5	5	4
4	4	5	3	2	4	4	4	4
4	4	3	4	3	5	4	5	3
4	4	4	3	3	3	4	4	4
2	2	2	2	2	3	3	3	2
3	4	4	4	4	3	3	4	5
5	4	4	4	5	5	5	5	5
2	4	2	4	4	3	4	4	4
2	3	3	4	2	4	1	4	3
3	2	3	3	2	1	3	2	3
5	5	4	5	5	5	5	5	3
3	4	4	4	4	4	4	4	3
5	5	5	5	5	5	5	5	5
4	4	3	4	5	3	3	4	5
4	4	5	5	5	5	5	4	4
2	4	5	4	3	3	4	4	3
3	4	5	3	5	4	3	5	4
2	3	4	3	4	3	2	3	3
3	3	2	2	2	3	3	2	2
3	3	3	4	3	3	1	3	3
4	4	4	4	5	5	4	4	5
4	4	4	4	4	4	4	4	5
1	4	3	4	3	2	2	4	4
4	4	4	4	5	5	4	4	5
4	4	4	4	5	5	4	4	5
3	4	4	5	4	5	4	4	4
5	5	3	5	5	5	5	5	5
4	5	3	5	5	5	5	4	4
3	4	4	4	4	3	3	3	4
1	4	3	5	5	3	3	3	3
4	4	3	4	4	4	4	4	4
3	3	2	4	3	1	3	3	5
2	4	4	4	3	4	4	3	3
3	3	2	3	2	4	1	2	4
4	4	4	4	4	4	5	5	5
3	3	3	4	4	4	4	4	5

1	5	3	3	5	5	3	3	5
4	4	2	4	4	4	4	4	3
4	3	2	4	4	4	3	3	4
4	4	2	4	4	4	4	4	4
3	4	2	4	4	3	3	4	2
3	5	3	4	5	5	4	5	4
4	4	3	3	4	4	5	5	4
3	4	4	3	3	3	4	4	3
5	5	3	5	5	4	4	4	5
4	4	3	3	4	4	3	4	3
3	4	4	4	4	3	3	3	4
3	3	3	4	4	4	4	4	4
3	3	3	4	4	4	3	5	3
4	4	5	5	5	5	4	5	3
4	4	5	5	5	5	4	5	3
4	4	2	2	4	4	2	3	3
4	4	4	4	4	4	4	4	4
3	2	2	2	2	2	2	2	2
4	4	4	4	4	4	4	4	4
3	4	4	4	3	4	3	4	4
4	3	3	4	4	4	4	3	3
4	3	3	4	4	4	4	3	3
4	4	3	4	4	4	4	4	4
4	4	5	4	4	4	4	4	4
4	4	3	4	4	5	5	5	4
4	4	4	4	4	4	4	3	4
4	4	3	4	4	4	4	4	4
4	4	3	4	3	4	4	5	5
3	3	4	4	4	4	3	4	3
2	4	2	4	4	3	4	4	3
2	5	3	4	4	3	3	4	4
5	3	4	4	5	3	4	4	4
3	4	5	5	5	4	4	4	4
4	5	3	4	5	4	4	4	3
3	3	4	4	4	3	4	4	3
4	4	4	4	4	4	3	4	4
5	5	5	5	5	5	5	5	5
3	3	4	3	3	4	3	3	4
3	4	4	4	4	4	4	4	4
5	3	4	3	5	2	3	4	2
3	3	4	4	5	3	5	4	3
4	4	5	2	3	1	3	4	4
4	4	2	3	3	4	3	3	3
4	4	4	4	4	4	4	4	4
4	5	4	4	4	4	4	4	4
5	5	5	5	5	5	5	5	5
3	3	3	3	4	3	3	4	2
3	3	4	5	3	4	4	4	3
4	4	5	5	4	4	5	4	4
3	3	3	4	3	3	3	3	4
3	3	3	3	3	2	3	3	2
3	3	3	4	3	3	3	3	2
5	5	5	5	5	4	5	5	5
3	3	4	4	4	4	3	3	3

4	4	4	4	4	4	4	4	4
3	5	4	5	5	4	4	5	5
5	4	5	4	4	4	5	4	5
3	3	3	3	3	3	3	3	3
3	3	2	5	4	4	3	5	3
4	4	4	4	4	4	4	4	4
5	5	5	4	5	5	5	5	4
4	4	5	5	4	3	3	4	4
4	4	4	4	4	4	4	4	4
5	5	3	4	5	2	4	5	5
4	3	4	4	4	4	4	4	4
3	3	3	4	3	3	3	3	4
4	3	2	3	4	4	4	4	4
5	4	5	5	4	4	4	4	5
5	5	5	5	5	4	5	5	5
5	3	3	4	4	3	3	3	3
3	3	2	4	4	2	4	3	4
4	4	3	4	4	4	4	4	4
4	4	3	4	4	4	3	4	5
3	4	3	3	3	3	3	4	4
4	2	3	4	4	4	4	4	4
4	3	4	4	4	4	3	3	4
2	3	4	3	2	4	3	2	4
3	3	3	4	4	2	3	4	3
3	3	3	3	4	3	3	3	3
3	4	3	4	4	3	2	3	2
4	4	3	4	4	4	3	4	4
4	4	4	4	4	4	4	4	4
4	4	4	4	4	4	4	4	4
3	3	3	3	5	5	5	5	5
3	4	4	4	4	4	4	4	4
3	3	3	4	4	4	4	4	4
4	5	4	4	5	5	5	4	5
4	4	4	4	4	4	4	4	4
3	2	3	3	4	3	4	3	3
5	4	4	3	3	1	5	5	5
3	4	3	5	4	3	3	4	4
4	4	4	4	4	3	4	4	3
4	4	5	4	5	3	3	4	3
2	3	2	3	2	3	2	3	2
2	3	4	5	1	2	3	4	5
4	3	4	4	4	4	3	4	4
4	4	2	4	4	3	4	4	4
3	4	4	4	4	4	4	4	3
4	4	4	5	4	5	4	4	4
4	4	2	3	5	4	4	4	3
5	3	5	4	4	4	4	4	3
5	5	4	4	4	5	5	5	4
5	5	5	5	5	5	5	5	5
4	5	3	5	5	4	5	5	5
4	5	3	5	5	5	5	5	5
3	3	2	3	2	3	4	3	4
4	4	2	4	4	3	4	4	4
4	4	4	4	4	4	4	5	4

4	4	3	3	3	3	3	3	2
5	5	5	5	5	5	5	5	5
3	5	3	4	3	3	3	3	3
3	2	4	3	2	4	3	2	3
5	4	5	5	5	4	4	4	4
3	3	3	3	3	3	3	3	3
3	3	4	3	4	3	4	3	3
4	3	2	3	3	3	3	3	3
4	4	4	4	4	4	4	4	4
4	4	3	4	4	4	4	4	4
4	3	4	4	4	4	3	3	2
4	5	3	5	5	5	5	5	4
4	4	3	3	3	3	1	3	3
4	4	4	4	4	4	4	4	4
4	4	4	4	4	4	4	4	4
4	4	4	4	4	4	4	4	4
4	4	4	4	4	4	4	4	4
4	4	3	4	3	4	3	4	3
4	4	4	4	4	4	4	4	4
4	4	4	4	4	4	4	4	4
4	4	4	4	4	4	4	4	4
3	2	2	3	4	2	2	5	4
3	2	2	3	2	2	3	3	2
2	2	4	3	4	3	4	3	3
3	4	2	4	4	3	2	3	3
4	3	2	4	4	4	4	4	4
5	5	5	5	5	5	5	5	5
3	4	4	4	3	5	3	3	4
2	5	2	4	4	4	4	4	5
3	4	2	4	4	2	2	4	3
3	3	3	4	4	3	3	3	3
3	3	3	3	3	3	3	3	3
3	3	3	3	3	3	2	3	4
4	4	4	4	4	4	4	4	4
4	3	4	3	4	3	4	3	4
2	2	3	2	4	3	4	4	3
3	3	3	3	3	3	3	3	3
4	4	4	5	4	5	4	4	5
2	5	4	5	4	5	5	5	5
3	3	3	3	3	3	3	3	3
4	4	4	4	4	4	4	4	3
4	4	4	4	4	4	4	4	4
4	4	5	4	5	4	5	5	4
4	4	3	4	4	4	3	3	4
4	4	4	4	4	4	4	4	4
2	2	2	2	2	2	2	2	3
4	4	4	4	4	4	4	4	4
4	3	4	3	4	3	4	3	3
3	3	3	3	3	3	3	3	3
3	3	3	3	3	3	3	3	3
3	3	3	3	3	3	3	3	3
5	5	5	5	5	5	5	5	5
4	4	4	4	4	4	4	4	5
3	4	2	4	3	4	3	4	3

4	4	4	4	4	4	4	4	4
5	5	5	5	5	5	4	5	5
5	5	4	5	4	5	5	5	4
5	5	5	5	5	5	5	5	5
4	4	3	3	4	4	4	3	3
4	4	3	4	4	4	4	4	4
4	4	4	4	4	4	4	4	4
4	4	4	4	4	4	4	4	4
4	3	3	4	2	4	4	2	4
3	3	3	4	4	4	3	4	3
4	3	4	4	5	5	5	5	4
5	5	5	5	5	5	5	5	5
3	3	3	4	4	3	4	4	3
3	5	5	5	3	5	4	3	5
3	4	4	4	4	4	4	4	3
4	2	3	3	3	4	4	4	2
3	3	3	3	3	3	3	3	3
4	3	4	3	4	4	5	4	4
4	4	3	4	4	4	3	4	4
3	3	3	3	3	3	3	3	3
2	4	4	3	4	4	2	4	4
4	4	4	4	4	4	4	4	4
4	5	3	4	4	4	3	5	4
3	2	1	5	1	5	1	5	1
3	3	5	4	4	4	4	3	4
5	5	4	4	5	5	5	5	5
4	4	3	4	3	3	4	3	3
5	5	5	3	5	5	5	4	3
5	5	5	5	5	5	5	5	5
3	3	3	3	5	4	4	4	5

Aa15	Aa16	Ab1	Ab2	Ab3	Ab4	Ab5	Ab6	Ab7
2	3	3	3	4	4	3	2	3
4	2	2	4	3	1	2	4	2
4	3	5	5	5	5	5	5	5
4	3	5	5	5	5	5	5	5
4	3	5	5	5	5	5	5	5
4	3	5	5	5	5	5	5	5
4	3	5	5	5	5	5	5	5
3	4	3	4	4	4	4	4	4
4	4	4	5	4	4	4	5	4
4	4	3	3	3	4	4	4	4
5	5	4	4	4	4	4	4	4
5	5	5	5	5	5	2	5	5
3	3	4	4	4	4	4	4	4
4	5	4	4	4	5	4	4	4
4	5	4	4	4	5	4	4	4
4	4	4	4	5	5	5	5	5
3	4	4	4	3	4	4	4	4
5	3	3	4	5	4	5	4	3
3	4	4	3	5	3	4	4	5
4	5	4	4	5	4	4	4	4
3	4	4	4	5	4	4	4	4
2	5	4	5	4	5	3	4	3
5	5	4	4	5	5	2	4	3
3	3	4	4	5	5	5	4	4
4	4	4	4	5	5	4	4	3
4	4	4	5	4	4	5	3	4
4	4	3	4	4	4	4	4	5
4	4	5	5	5	5	5	5	5
4	4	4	4	3	4	3	4	4
4	3	2	3	4	5	4	3	2
2	3	3	3	3	2	3	2	2
3	4	3	4	5	4	4	4	5
4	4	5	4	4	5	5	5	5
5	5	4	5	4	3	4	3	5
3	4	4	5	5	4	5	5	5
4	4	5	4	4	3	5	4	3
3	3	3	3	4	3	3	4	4
3	4	4	4	4	4	4	4	4
4	4	4	5	4	5	4	5	5
4	5	5	5	5	4	4	5	5
4	4	4	4	4	4	4	4	4
3	4	2	3	4	2	3	2	4
4	5	4	4	5	5	4	4	4
2	5	5	5	4	4	5	5	5
2	3	4	2	3	5	2	4	3
5	5	5	5	4	4	4	4	4
4	4	4	4	4	5	4	4	4
4	5	3	4	4	5	4	4	4
4	5	4	4	3	4	3	3	4
2	3	4	4	4	5	5	5	5
4	4	5	5	5	5	5	5	5

4	4	4	4	4	4	4	4	4
4	4	4	4	4	5	4	4	4
4	3	4	3	4	4	4	4	3
5	4	4	4	3	3	4	5	5
4	3	4	3	3	3	4	3	4
5	4	3	4	5	5	4	4	3
3	3	4	4	4	5	5	4	5
4	5	4	4	5	4	5	5	4
4	4	4	4	5	5	4	4	4
4	4	4	3	5	3	3	4	4
4	4	4	4	4	4	4	4	4
1	1	3	2	3	3	2	1	1
4	4	2	4	4	4	2	4	3
3	3	2	4	3	2	4	4	3
5	5	5	5	5	5	5	5	5
4	4	4	4	4	4	4	4	4
5	5	4	4	5	4	4	5	5
5	5	5	5	5	5	5	5	5
4	5	4	4	4	5	4	5	5
3	3	4	4	4	4	3	3	3
4	4	4	4	4	4	2	4	4
4	4	4	4	4	4	3	5	5
3	3	4	4	4	4	4	4	4
5	4	4	4	4	5	4	5	3
4	4	2	3	4	3	3	3	4
5	5	4	4	5	4	2	4	4
4	4	5	5	5	5	5	5	5
4	4	4	4	4	4	3	4	3
4	4	4	4	4	4	3	4	4
3	5	5	5	5	5	5	5	5
3	4	4	5	5	5	5	5	5
5	4	5	5	5	4	4	4	4
4	4	5	4	5	5	4	5	5
5	5	5	5	4	4	4	5	4
4	4	4	4	4	4	4	4	4
4	4	4	4	4	4	4	4	4
4	4	3	3	3	3	3	3	3
4	4	4	4	4	4	4	4	4
3	3	3	3	3	3	3	3	3
4	4	5	5	5	5	5	5	5
4	4	4	4	4	5	3	4	5
5	5	5	5	5	5	5	5	5
5	5	5	5	5	5	5	5	5
4	4	3	4	4	4	4	4	4
4	4	3	5	4	5	5	4	4
4	4	4	4	5	5	4	4	4
5	4	4	4	4	5	2	4	4
4	4	4	5	5	5	4	4	4
4	4	4	5	4	5	2	4	5
4	4	4	4	4	3	2	4	4
4	4	4	4	4	4	4	4	5
4	4	4	5	5	4	4	4	3
4	4	4	5	5	4	4	4	3
4	5	4	5	4	5	2	5	5

3	4	4	4	4	4	3	4	4
3	3	3	4	4	4	3	4	3
5	5	5	5	5	5	5	5	5
4	3	4	4	4	3	3	3	3
4	4	4	4	4	5	3	4	4
5	4	2	2	2	3	3	3	3
3	3	4	4	4	3	4	4	4
4	4	4	3	4	4	4	4	4
4	4	4	4	4	4	4	4	4
5	5	5	5	5	5	5	5	5
3	3	4	4	4	4	3	3	3
4	4	4	4	4	4	4	4	4
3	3	3	3	3	3	3	3	3
3	3	4	4	4	4	3	4	4
3	4	4	4	4	4	2	4	4
2	3	3	4	4	4	4	4	3
5	5	4	4	5	5	5	5	4
4	4	4	4	4	4	4	4	4
4	4	4	4	4	4	4	4	4
4	4	4	3	2	4	3	4	3
4	5	5	5	4	5	4	3	5
5	5	5	4	4	4	4	4	4
2	2	3	4	5	4	3	2	1
3	4	3	4	5	5	4	4	5
5	5	5	5	5	5	5	4	4
4	4	4	4	4	4	3	3	3
3	1	5	5	5	4	4	5	3
4	5	4	2	3	3	2	2	4
5	5	4	4	4	5	5	5	4
3	3	4	4	3	5	5	5	3
5	5	5	5	5	5	5	5	5
4	3	4	4	4	3	3	4	4
5	5	5	5	5	4	4	5	5
3	5	3	4	5	3	4	3	5
3	4	3	4	5	4	4	3	4
4	3	4	4	3	5	2	3	2
3	3	4	4	4	4	4	4	4
3	3	4	4	4	4	4	4	4
5	5	5	5	5	5	4	5	5
4	4	4	4	4	5	3	4	4
2	5	4	4	4	5	3	4	3
5	5	5	5	5	4	5	5	5
5	5	5	5	5	5	4	5	5
4	4	5	4	5	5	4	4	4
5	5	5	5	5	5	5	5	5
4	5	5	5	4	4	3	5	5
3	4	4	4	4	4	3	3	3
3	4	4	4	4	4	3	4	4
3	4	5	5	4	4	4	4	4
5	5	4	4	4	4	4	4	4
3	4	4	4	5	4	4	5	4
2	4	3	4	4	4	3	4	4
5	5	3	4	3	5	5	5	3
4	4	4	4	4	4	4	4	4

5	5	5	2	5	5	4	4	5
4	4	4	4	5	5	5	5	5
3	4	3	4	4	4	2	4	4
4	3	4	4	4	4	4	4	5
4	4	4	4	4	4	4	4	4
4	5	4	4	4	4	4	4	4
5	4	4	4	4	4	5	4	4
3	3	4	5	5	4	4	5	4
5	4	5	5	5	5	5	5	5
4	4	5	4	4	5	5	4	5
4	4	4	4	4	4	2	4	4
4	4	4	4	4	4	4	4	4
4	3	3	5	5	5	4	5	5
4	5	3	4	4	5	4	5	4
4	5	4	4	5	4	5	4	4
3	4	4	4	4	4	4	4	4
4	4	4	4	4	4	4	4	4
2	2	4	4	5	5	4	5	4
4	4	3	4	4	5	4	4	3
4	4	4	4	5	5	4	5	5
4	4	5	5	5	5	4	4	4
4	4	5	5	5	5	4	4	4
4	4	5	5	5	5	5	5	5
4	4	5	5	5	5	5	5	5
5	4	4	4	4	4	5	4	4
3	4	4	4	4	4	3	4	4
4	4	4	4	4	4	4	4	5
5	5	5	5	5	5	5	5	5
4	4	4	4	4	4	4	4	4
4	4	4	4	4	4	3	4	4
4	5	4	4	4	4	4	4	5
4	5	4	5	5	5	4	5	5
4	4	5	5	5	5	5	5	5
4	5	4	4	4	4	4	4	4
4	4	4	4	4	4	4	4	4
4	4	3	4	4	4	4	5	4
5	5	5	5	5	5	5	5	5
4	4	5	5	5	5	4	4	4
4	4	4	4	4	5	3	5	4
3	4	3	4	3	4	2	3	5
3	5	5	5	5	5	3	5	4
3	3	5	5	5	5	4	4	4
4	4	4	4	4	4	5	5	4
4	4	4	4	4	4	4	4	4
4	4	5	5	5	5	5	5	5
5	5	5	5	5	5	5	5	5
4	4	4	4	4	5	5	5	5
4	5	5	4	4	5	3	4	4
4	4	4	4	4	4	3	4	4
4	3	4	4	4	4	4	4	4
3	4	3	3	3	3	3	3	4
4	3	5	5	5	4	5	4	5
5	5	5	5	5	4	5	4	5
5	4	4	5	4	5	5	4	4

4	4	4	4	4	4	4	4	4
5	5	4	3	4	3	3	2	4
4	5	4	4	4	4	4	4	4
3	3	4	4	4	4	4	4	4
3	4	5	4	4	5	4	4	4
4	4	4	4	4	4	4	4	4
4	5	5	5	5	5	4	5	3
4	4	4	4	4	3	4	3	4
4	4	5	5	4	4	4	4	4
5	4	3	4	4	5	4	4	5
4	4	4	5	5	4	4	4	5
4	3	4	4	4	4	4	4	4
4	4	3	4	3	3	3	2	4
5	4	4	4	3	3	5	4	3
5	5	5	5	5	5	5	5	5
5	4	4	5	4	5	5	4	4
4	4	4	4	4	4	3	5	5
4	4	4	4	4	5	5	5	5
3	5	5	5	4	4	4	4	4
3	3	4	4	4	4	4	5	4
4	4	4	4	4	4	4	4	4
2	4	4	4	4	4	4	4	4
2	3	3	3	4	4	4	3	4
4	4	3	3	3	2	3	3	2
3	3	4	4	4	4	4	4	4
3	3	4	4	3	4	3	4	4
4	4	4	4	4	4	4	5	5
4	4	4	4	4	4	4	4	4
4	4	4	4	4	5	4	5	4
5	5	4	4	4	4	4	4	5
4	4	4	4	4	4	5	5	5
4	4	4	4	4	5	4	4	5
5	5	5	4	5	4	5	5	4
4	4	4	4	4	4	4	4	4
3	3	4	4	4	5	4	5	4
5	5	5	5	5	5	5	5	4
4	3	2	3	4	4	4	4	5
4	4	5	4	4	5	4	4	4
4	3	4	5	4	4	5	4	5
3	2	4	4	4	4	4	4	4
3	2	1	2	3	4	5	5	4
3	4	4	4	4	4	4	4	4
4	4	4	4	4	4	4	4	5
3	4	3	4	4	4	4	3	3
4	4	4	4	4	3	4	4	4
4	4	4	4	5	5	5	5	5
4	4	4	5	5	5	4	5	5
4	5	4	4	5	5	4	4	4
5	5	4	4	4	4	4	4	4
4	5	4	5	5	4	4	5	5
5	5	4	4	5	5	5	5	5
4	5	4	4	4	5	5	5	4
4	4	4	4	4	4	4	4	4
5	4	4	5	5	4	5	5	5

4	4	4	4	4	4	4	4	4
5	5	5	5	5	5	5	5	5
3	3	3	3	3	3	3	3	3
4	2	4	3	2	1	3	4	3
4	4	4	4	4	4	4	4	4
3	3	4	4	4	4	4	4	4
4	3	4	3	4	3	4	3	4
3	4	5	5	3	5	2	4	4
4	4	4	4	4	4	4	4	4
3	4	4	4	4	4	4	4	4
1	2	3	3	3	3	4	4	4
5	4	3	5	5	5	4	5	4
3	3	4	4	4	4	3	3	3
4	4	3	3	3	3	3	3	4
4	4	3	3	3	3	3	4	4
4	4	3	3	3	3	4	4	4
4	4	4	3	3	3	3	3	3
4	3	4	4	4	5	5	5	4
4	4	3	3	3	3	3	4	4
4	4	3	3	3	3	4	4	4
4	4	4	3	3	3	3	3	3
2	3	1	3	4	3	2	3	2
2	3	1	4	4	2	3	3	2
3	3	4	3	4	4	4	4	4
3	4	4	4	4	4	4	4	4
3	4	4	4	4	4	4	3	3
5	5	4	4	4	4	5	5	5
4	4	5	5	5	5	4	4	4
3	5	5	5	5	5	4	4	4
4	4	4	4	5	5	4	4	4
3	3	4	4	4	4	4	4	4
3	3	3	3	3	3	3	3	3
3	3	4	4	4	4	4	4	4
4	4	4	5	4	5	4	5	4
3	4	4	4	4	4	4	3	4
4	3	3	4	3	3	4	3	2
3	3	3	3	3	3	3	3	3
5	5	5	5	5	5	5	5	5
4	5	5	4	5	5	5	5	5
3	3	3	3	3	3	4	3	4
4	3	3	3	4	4	4	3	4
4	4	4	4	4	4	4	4	4
4	5	5	5	5	5	4	4	5
4	4	4	4	4	4	4	4	4
4	4	4	4	4	4	4	4	4
2	4	4	4	4	3	4	3	3
4	4	4	4	4	4	4	4	4
4	3	4	4	4	4	4	5	4
3	3	4	3	4	3	3	3	3
3	3	3	3	3	3	3	4	4
3	3	3	3	3	3	3	3	3
5	5	5	3	5	5	5	5	5
5	5	4	4	5	5	5	5	5
3	3	4	3	3	4	3	4	3

4	4	4	4	4	4	4	4	4
5	5	5	5	5	5	5	5	5
4	5	5	5	5	5	5	5	5
5	5	5	5	5	5	5	5	5
3	3	3	3	3	3	4	4	3
3	3	4	4	4	5	4	5	5
4	4	4	4	4	4	4	4	4
4	4	4	4	4	4	4	4	4
2	4	4	2	4	4	3	3	4
5	3	3	3	3	3	3	3	3
4	5	5	4	4	4	4	4	4
5	5	4	4	4	4	3	4	4
3	3	5	5	5	5	5	5	5
3	3	3	4	4	3	4	3	3
4	4	5	5	5	5	5	5	5
4	2	4	4	2	3	4	4	4
3	3	3	3	3	3	3	3	3
5	4	2	4	5	5	4	4	4
4	4	3	4	4	5	4	4	4
3	3	4	4	4	4	4	4	4
4	4	4	4	4	4	4	5	5
4	4	4	4	4	5	5	5	5
4	4	4	5	5	5	3	4	4
3	3	3	2	2	3	5	1	5
3	4	4	3	4	3	3	4	3
5	5	4	4	4	5	5	5	5
3	3	3	3	3	3	3	3	3
3	5	5	3	3	3	4	4	5
5	5	5	5	4	4	5	4	4
5	4	5	4	4	4	4	4	4

Ab8	Ab9	Ab10	Ab11	Ab12	Ab13	Ab14	Ab15	Ab16
4	2	2	4	4	4	4	3	3
4	1	5	2	4	1	3	2	5
5	5	5	4	4	4	4	4	4
5	5	5	4	4	4	4	4	4
5	5	5	4	4	4	4	4	4
5	5	4	4	4	4	4	4	4
5	5	5	4	4	4	4	4	4
4	4	4	4	3	4	4	3	3
4	5	4	4	4	5	4	4	4
4	4	4	4	4	4	4	4	4
4	4	5	5	4	5	5	5	5
5	2	5	5	5	5	5	2	5
4	4	4	4	4	4	4	4	4
4	2	4	4	5	5	5	5	4
4	2	4	4	5	5	5	5	4
5	5	4	5	4	5	5	4	5
4	4	4	4	3	4	3	3	4
4	5	4	3	4	5	5	5	4
4	4	5	3	4	5	3	5	3
3	4	4	5	4	3	3	3	4
5	4	4	4	4	4	5	4	4
4	5	5	5	3	3	4	5	3
5	4	4	4	4	4	4	4	4
3	4	4	5	5	5	5	5	5
4	4	5	5	5	5	5	5	5
4	4	5	4	3	4	5	4	5
5	5	5	5	5	5	4	4	4
5	4	5	5	4	5	4	4	5
3	3	4	4	4	4	4	3	3
3	4	4	4	3	3	3	4	5
3	3	4	3	3	4	2	5	4
5	5	4	5	4	4	3	4	5
5	5	5	4	4	3	3	4	5
5	3	4	4	3	4	5	4	3
4	5	4	5	4	5	5	4	5
4	3	4	4	5	4	4	5	4
4	3	4	4	4	3	4	4	4
4	2	4	4	4	4	4	3	4
4	5	4	5	5	5	5	4	4
5	5	5	5	5	5	5	5	4
4	4	3	4	4	5	5	5	4
2	4	2	4	2	4	2	2	2
5	4	4	4	5	4	4	4	4
4	5	4	4	4	4	4	3	3
3	2	2	3	2	3	1	2	3
4	4	3	3	4	5	5	5	5
4	4	5	5	4	4	3	2	4
3	4	3	4	5	5	5	4	4
4	4	3	3	3	4	3	3	4
4	4	4	4	4	5	5	5	4
5	5	5	4	5	4	5	5	4

3	3	3	3	3	3	5	5	5
4	5	4	4	5	5	4	4	4
3	3	3	4	4	3	3	3	4
5	4	4	4	5	4	5	4	5
3	3	3	3	3	3	3	3	3
4	5	5	4	4	3	3	4	5
4	5	4	4	5	5	5	4	4
5	5	4	5	4	5	4	5	4
3	2	3	4	4	2	3	3	3
5	5	4	5	5	5	5	5	5
4	4	4	4	4	4	4	4	4
3	3	3	3	3	3	3	3	3
4	4	4	4	4	4	4	2	4
2	5	4	3	3	3	2	4	3
5	5	5	5	5	5	5	4	5
4	4	4	4	4	4	4	4	4
5	5	5	5	5	5	5	5	5
5	5	5	5	5	5	5	5	5
5	5	4	4	4	5	4	5	4
3	3	3	3	3	3	3	3	3
4	4	4	4	4	4	4	4	5
4	4	5	5	5	4	4	5	5
4	3	3	3	3	3	3	3	3
4	4	4	5	5	5	4	4	4
3	2	3	3	2	3	3	3	4
4	4	3	4	4	5	5	4	3
5	5	5	5	5	5	5	5	5
4	4	4	4	4	4	4	4	4
4	3	4	4	5	5	5	3	5
5	4	5	5	5	5	5	3	5
5	5	5	5	5	5	5	5	5
4	4	5	4	4	4	4	4	3
5	5	5	5	5	5	5	5	5
4	3	4	5	3	4	3	5	3
4	4	4	4	4	4	4	4	4
4	3	5	5	3	5	3	4	4
3	3	3	3	3	3	3	3	3
4	5	4	4	4	4	4	4	4
3	3	3	3	3	3	3	3	3
4	5	4	4	4	4	5	5	5
5	2	5	4	4	4	5	4	4
5	5	5	5	5	5	5	5	5
5	5	5	5	5	5	5	5	5
4	4	4	4	4	4	4	4	4
4	4	5	4	5	4	4	4	4
4	4	4	5	4	5	5	4	5
5	5	4	4	4	5	3	3	4
4	4	4	4	4	4	5	5	5
4	4	4	4	4	5	5	4	4
4	3	4	4	4	4	4	4	4
5	4	4	4	5	4	4	4	4
4	3	4	4	3	3	4	3	3
4	3	4	4	3	3	4	3	3
5	4	4	5	5	5	4	3	4

4	4	4	4	4	4	4	4	4
4	3	4	4	4	3	3	3	4
5	4	4	4	5	5	4	5	4
3	3	3	3	3	3	3	3	3
4	3	4	4	4	4	4	3	4
3	3	3	3	3	4	4	3	4
3	4	4	4	4	3	3	4	4
4	4	4	4	4	4	4	4	4
4	4	4	4	4	4	4	4	4
5	5	5	5	5	5	5	5	5
3	3	4	4	3	4	3	4	4
4	4	4	4	4	4	4	4	4
4	4	3	3	4	3	3	4	3
4	4	4	4	4	4	4	4	4
4	2	4	4	4	4	4	4	4
4	4	4	3	3	2	3	3	4
4	4	4	4	4	4	5	5	5
4	4	4	4	5	5	4	5	4
4	4	4	5	5	5	5	5	5
4	3	4	2	4	4	2	4	3
4	3	4	4	4	5	5	5	4
4	5	5	5	5	5	4	4	4
3	5	3	4	5	4	3	4	5
5	3	4	5	4	5	4	3	4
5	5	4	4	5	5	4	5	5
3	3	3	4	4	3	3	3	3
5	4	3	5	3	4	3	4	5
4	2	2	3	3	3	5	5	2
3	5	5	5	5	5	5	5	5
4	3	4	3	3	4	4	4	4
5	5	5	5	5	5	5	5	5
4	4	3	5	5	5	5	3	4
4	5	4	4	4	5	5	4	5
3	4	2	3	3	4	4	3	3
5	4	5	4	5	3	4	5	4
3	3	2	3	3	4	2	3	4
4	4	4	3	3	3	3	3	3
4	4	4	4	4	4	4	4	4
5	4	5	5	5	5	5	2	4
4	3	4	5	4	5	4	4	4
4	3	3	4	4	4	3	4	4
4	5	5	5	5	5	5	2	4
5	4	5	5	5	5	5	2	4
5	4	4	4	5	5	5	4	5
5	4	5	5	5	5	5	4	5
5	3	5	5	4	4	4	4	4
3	4	3	4	3	4	3	3	4
5	2	3	4	4	4	4	4	4
4	4	4	4	4	5	5	5	4
4	4	4	4	3	4	3	3	2
4	4	4	4	3	4	4	4	4
3	4	4	3	4	3	4	2	4
4	4	4	4	3	4	4	5	4
4	3	4	4	4	4	4	4	4

4	4	4	4	4	4	4	2	5
5	4	4	5	4	5	5	5	5
4	3	4	4	4	5	4	3	4
4	5	4	4	5	4	4	5	4
4	4	4	5	5	5	5	4	5
4	3	4	4	4	4	4	4	4
4	4	4	5	4	5	5	5	5
4	5	4	4	4	5	4	5	5
5	5	5	5	5	5	5	4	5
5	5	4	3	4	4	5	5	5
4	4	4	4	4	4	4	3	5
4	4	4	4	4	4	4	4	4
5	4	4	5	5	5	5	2	5
4	4	5	5	4	4	4	4	3
4	5	5	4	4	4	4	4	3
4	2	4	4	4	4	4	2	4
4	4	4	4	4	4	4	4	4
5	4	3	5	5	4	3	4	5
5	5	4	5	5	4	5	3	5
4	5	4	4	5	5	4	5	4
5	5	5	5	5	5	5	4	4
5	5	5	5	5	5	5	4	4
5	5	5	5	5	5	5	5	5
5	5	5	5	5	5	5	5	5
4	4	4	5	4	5	5	5	5
4	3	4	4	4	4	3	4	4
4	5	4	5	4	4	4	5	4
5	5	5	5	5	5	5	3	3
4	4	4	4	4	4	4	4	4
4	4	5	5	5	5	4	4	5
4	4	5	4	4	5	4	4	4
4	5	5	5	5	5	5	4	5
5	5	5	5	5	5	5	5	5
5	5	5	5	5	5	5	5	4
4	4	4	4	4	4	4	4	4
4	4	4	4	4	5	4	3	5
5	5	5	5	5	5	5	5	5
5	5	5	5	5	5	5	5	5
5	5	4	5	5	5	4	4	4
3	4	2	3	4	3	4	3	2
5	5	5	5	4	5	5	5	5
3	4	3	4	4	5	3	4	3
4	4	5	5	4	5	5	5	4
4	4	4	4	4	4	4	4	4
5	5	5	5	5	5	5	5	5
5	5	5	5	5	5	5	5	5
5	3	4	3	4	3	4	3	4
5	3	4	4	4	4	5	3	4
4	4	3	3	3	4	4	3	3
4	4	4	4	4	4	4	4	4
4	4	3	4	3	4	3	4	4
5	5	4	4	5	5	5	5	5
5	5	4	4	5	5	5	5	5
4	3	4	4	4	4	3	4	4

4	4	4	4	4	4	4	4	4
4	4	4	5	5	5	4	3	3
4	4	5	5	4	5	4	4	4
4	4	4	4	4	4	4	4	4
4	4	3	4	4	4	4	4	4
4	4	4	4	4	4	4	4	4
5	5	5	5	5	5	5	2	5
5	4	3	3	4	4	3	4	4
4	4	5	5	5	4	4	4	4
5	4	4	4	5	5	5	3	4
5	4	4	5	4	4	4	4	4
4	4	4	4	4	4	4	4	4
3	3	4	4	3	3	2	5	3
5	5	3	4	4	3	4	3	5
5	3	5	5	5	5	5	5	5
4	3	4	4	4	3	4	4	4
4	4	5	4	5	5	5	4	3
5	5	5	5	5	5	5	5	5
5	5	5	5	4	4	4	4	4
4	4	4	5	4	4	3	3	4
4	4	4	4	4	4	4	4	4
3	4	3	3	4	3	3	2	4
4	3	3	4	3	4	3	4	3
4	3	3	4	3	3	2	3	2
4	4	4	4	4	4	4	4	4
4	5	3	4	3	5	5	3	4
4	4	4	4	4	4	5	4	4
4	4	4	4	4	4	4	4	4
5	4	5	4	5	4	4	5	4
5	5	5	5	5	5	5	5	5
5	5	4	5	4	5	4	4	4
5	5	5	5	5	5	5	5	5
4	5	5	4	5	5	5	4	5
4	4	4	4	4	4	4	2	4
5	4	4	5	4	5	4	5	4
5	5	4	5	5	5	4	4	1
5	4	5	4	4	4	5	4	4
5	4	4	4	4	4	4	4	4
4	5	4	4	5	4	4	4	4
4	4	4	4	4	4	4	4	4
3	2	1	2	3	4	5	5	4
5	5	5	5	5	5	5	5	5
5	5	4	4	4	4	4	4	4
3	4	4	4	3	4	3	4	4
4	4	4	4	4	4	4	4	4
5	5	3	4	4	4	5	5	5
5	4	5	5	5	4	4	5	5
5	4	5	5	4	4	4	5	5
4	5	4	4	4	5	4	4	4
5	5	5	5	5	5	5	4	5
5	4	5	4	5	5	5	5	4
5	4	5	4	4	4	3	4	4
4	4	4	4	4	4	4	4	4
5	5	5	4	4	4	4	5	4

4	4	4	4	4	4	4	5	4
5	5	5	5	5	5	5	5	5
3	3	3	3	3	3	3	3	3
3	3	4	4	4	2	2	2	4
4	4	4	4	4	4	4	4	4
4	4	4	4	4	4	4	4	4
4	4	4	4	4	3	4	4	3
5	2	4	5	5	5	5	5	5
4	4	4	4	4	4	4	4	4
4	3	4	4	4	4	4	2	4
3	4	3	4	5	5	4	4	4
5	4	4	4	5	5	5	4	5
4	3	4	3	4	3	4	3	4
4	4	4	4	4	3	3	3	4
4	4	3	3	4	4	3	3	4
4	4	4	3	3	3	4	4	4
3	4	4	4	4	4	4	4	4
4	4	5	5	4	4	5	4	4
4	4	3	3	4	4	3	3	4
4	4	4	3	3	3	4	4	4
3	4	4	4	4	4	4	4	4
3	2	3	2	3	3	4	4	4
5	2	4	3	2	2	3	4	3
4	3	4	5	4	5	4	5	4
4	4	4	4	4	4	4	4	4
3	4	4	4	4	4	4	4	3
5	5	5	5	4	4	4	4	4
4	3	3	3	4	4	5	4	4
5	3	5	5	5	5	5	4	5
4	3	4	5	3	4	3	3	4
4	4	4	4	4	4	4	4	4
3	3	3	3	3	3	3	3	3
4	4	4	5	4	4	5	5	5
4	5	4	4	4	4	4	4	4
4	4	4	4	4	4	4	4	4
4	4	4	3	4	3	4	4	4
3	3	3	3	4	4	4	4	4
4	5	4	4	5	4	4	5	5
5	5	5	5	5	4	4	3	4
2	4	4	4	4	4	4	4	5
4	4	4	4	4	4	4	4	4
4	4	4	4	4	4	4	4	4
5	4	5	5	4	5	5	5	5
4	3	3	4	4	4	4	3	4
4	4	4	4	4	4	4	4	4
4	4	4	4	4	4	4	4	4
4	4	4	4	4	4	4	4	4
4	4	4	5	4	5	4	5	4
3	3	3	3	3	3	3	3	3
4	3	3	4	3	4	3	4	3
3	3	3	3	3	3	3	3	3
5	3	5	5	5	5	5	5	5
5	5	5	5	5	5	5	5	5
3	3	4	4	4	4	4	3	3

4	4	4	4	4	4	4	4	4
5	4	4	5	5	5	5	5	5
5	5	5	5	4	4	4	5	4
5	5	5	5	5	5	5	5	5
4	3	4	4	4	4	4	4	4
5	4	5	5	5	5	5	4	5
4	4	4	4	4	4	4	4	4
4	4	4	4	4	4	4	4	4
4	4	4	3	4	3	4	4	4
3	3	3	3	3	3	3	4	4
4	4	4	4	4	4	4	4	4
4	4	4	4	4	4	4	4	4
5	5	5	5	5	5	5	5	5
4	4	3	3	4	3	4	3	3
5	5	5	5	5	5	5	5	5
3	4	4	4	4	4	4	4	3
3	3	4	4	4	4	4	4	4
4	4	4	4	4	4	4	2	5
4	3	4	4	4	5	5	5	5
4	4	4	4	4	4	4	4	4
3	4	3	5	5	4	4	4	4
5	5	5	5	5	5	5	5	5
4	4	5	5	3	4	4	4	4
1	5	1	5	1	5	1	5	1
4	3	4	4	5	5	4	3	4
5	5	4	4	4	4	4	4	4
3	3	3	3	4	3	3	3	3
3	4	4	4	5	5	4	4	4
4	5	4	4	4	4	3	4	3
4	4	5	5	5	4	5	3	4

Ab17	assessment literacy(A)	B1	B2	B3	B4	B5	B6	B7
3	3. 12	3	4	4	4	4	4	4
2	2. 64	2	3	3	2	3	2	4
4	4. 15	3	4	5	4	3	2	3
4	4. 18	3	4	5	4	3	2	3
4	4. 21	3	4	5	4	3	2	3
4	4. 09	3	4	5	4	5	2	5
4	4. 09	3	4	5	4	3	2	4
4	3. 48	4	4	3	3	3	4	4
4	4. 00	4	3	3	4	4	3	4
4	3. 64	3	4	4	4	3	4	3
4	4. 33	4	4	5	5	4	4	5
5	4. 33	5	5	5	5	5	5	4
4	3. 70	4	4	4	4	4	4	4
5	4. 30	4	5	4	4	3	4	4
5	4. 30	4	5	4	4	3	4	4
4	4. 12	4	4	4	4	4	5	5
4	3. 61	4	4	4	4	4	4	4
4	4. 15	3	4	5	5	5	4	4
4	4. 00	3	5	4	5	3	5	5
3	3. 85	4	5	5	4	4	5	5
4	3. 97	4	4	4	4	4	4	4
5	3. 94	4	4	4	5	4	3	5
5	4. 09	4	4	4	5	5	5	5
5	4. 12	4	4	4	4	4	4	5
5	4. 21	4	4	4	4	4	4	4
4	4. 24	4	4	3	4	4	4	4
4	4. 33	4	4	4	4	5	4	4
5	4. 36	5	4	5	5	5	5	5
4	3. 82	4	4	4	4	4	4	4
4	3. 52	3	4	5	4	3	2	2
5	3. 03	4	5	4	5	4	4	4
4	3. 85	3	4	5	4	4	4	5
4	4. 45	4	4	5	5	5	4	4
5	3. 94	3	4	5	4	4	4	5
4	4. 12	5	5	5	5	5	5	5
3	3. 70	4	4	4	4	4	5	4
3	3. 33	4	4	4	3	4	4	4
4	3. 70	4	4	4	4	4	4	4
4	4. 06	4	4	5	5	5	4	3
5	4. 45	5	5	5	5	5	5	4
4	3. 79	4	5	4	4	4	4	4
4	2. 76	3	2	4	2	4	3	3
4	4. 12	5	5	5	5	5	5	5
3	4. 03	5	4	4	4	4	4	4
3	2. 70	4	3	3	4	3	3	3
5	4. 36	5	5	5	4	4	4	4
4	4. 00	4	4	4	4	4	4	4
5	4. 00	5	5	4	4	4	5	4
4	3. 70	4	4	4	5	5	5	4
4	4. 09	4	4	4	4	3	3	5
5	4. 18	5	5	5	5	4	4	4

5	3.94	4	4	3	5	4	4	4
5	4.06	4	5	4	4	4	5	5
4	3.61	4	5	5	5	5	4	4
4	4.18	4	5	4	3	3	4	5
3	3.15	4	4	3	4	3	4	3
5	4.06	4	5	4	3	4	5	4
5	3.97	4	4	5	5	4	4	5
5	4.42	4	4	5	4	5	4	5
3	3.36	4	4	4	4	4	4	4
5	4.00	5	5	5	5	5	5	4
4	3.94	4	4	4	4	4	4	4
3	2.36	3	3	3	3	3	2	2
4	3.42	4	4	4	4	4	3	4
2	3.09	4	3	4	3	2	4	3
5	4.85	5	5	5	5	5	5	5
4	4.00	4	4	4	4	4	4	4
5	4.82	5	5	5	5	5	5	5
4	4.79	5	5	5	5	5	5	5
4	4.27	4	4	4	4	4	4	4
3	3.18	3	4	4	4	4	4	4
4	4.00	5	5	5	5	5	5	5
5	4.36	5	5	4	4	5	5	4
3	3.42	3	3	3	4	4	4	4
3	3.94	3	4	4	4	4	4	4
3	3.06	3	3	3	3	3	3	3
4	4.12	4	4	4	4	4	4	4
5	4.33	5	5	5	5	5	5	4
4	3.76	4	4	4	4	4	3	3
4	3.85	4	4	4	4	4	4	3
5	4.55	4	5	4	4	4	4	3
5	4.06	4	4	4	4	4	4	4
4	4.24	4	4	4	3	3	4	4
5	4.48	4	4	4	4	4	4	4
5	4.36	4	4	3	4	3	4	3
4	3.91	4	4	4	4	4	4	4
3	3.97	4	4	4	4	4	4	4
3	3.42	4	4	4	4	4	4	4
4	4.09	4	4	4	4	4	4	3
3	3.00	4	4	4	4	4	4	4
5	4.70	5	4	4	5	4	4	4
5	3.91	4	5	5	5	5	4	4
5	4.58	4	4	4	3	4	4	4
5	4.55	4	4	4	3	4	4	4
4	3.88	4	4	5	4	4	3	4
4	4.03	5	4	4	4	4	4	4
5	4.33	4	5	4	4	4	4	5
5	4.06	5	5	4	4	3	4	4
5	4.09	4	4	4	4	4	4	4
4	3.88	4	4	4	3	4	4	4
4	3.85	4	4	4	4	4	4	4
4	4.18	4	4	4	4	4	5	5
4	3.82	4	5	4	5	4	5	4
4	3.85	4	5	4	5	4	5	4
5	4.12	4	4	4	3	3	4	4

3	3.64	4	4	4	4	4	4	4
3	3.42	4	4	3	4	3	4	4
5	4.61	5	5	5	4	4	5	5
3	3.33	3	3	3	4	3	4	3
3	3.64	4	4	4	4	4	4	5
4	3.09	3	3	4	3	4	4	4
4	3.45	4	4	4	4	4	4	4
4	3.79	3	4	4	4	4	4	4
4	3.82	4	4	4	4	4	4	4
5	5.00	4	4	5	5	4	4	5
4	3.21	5	5	5	5	5	5	5
4	3.82	4	4	4	4	4	4	4
3	3.21	3	3	3	4	4	4	3
4	3.76	4	4	3	4	4	4	3
3	3.55	4	4	4	4	4	4	4
4	3.42	3	4	4	3	3	2	3
5	4.52	5	5	5	5	5	5	5
5	4.06	4	4	4	5	4	5	5
5	4.39	5	5	4	5	4	4	5
4	3.52	4	5	3	4	3	4	3
4	4.24	5	4	5	5	4	4	4
4	4.30	4	4	4	4	4	4	4
4	3.09	5	4	3	2	1	2	3
5	3.91	4	3	4	4	5	3	3
5	4.64	5	5	5	5	4	4	4
3	3.48	4	5	4	4	4	5	5
5	3.61	5	5	5	5	4	4	4
1	2.85	2	3	4	3	3	2	1
5	4.73	4	4	4	5	5	5	5
4	3.79	4	4	4	3	3	3	4
5	5.00	5	5	5	5	4	4	5
3	3.91	5	5	5	5	5	5	5
5	4.61	4	5	4	5	5	4	5
3	3.48	4	5	4	3	4	5	4
3	3.88	4	5	4	4	5	4	5
2	3.12	3	2	4	2	4	3	2
3	3.15	3	3	4	4	4	4	4
4	3.45	3	3	4	4	3	3	4
4	4.39	4	5	4	4	5	5	4
4	4.00	3	3	3	3	3	3	4
4	3.36	4	4	4	4	4	4	4
4	4.39	4	5	4	4	5	5	4
4	4.39	4	5	4	4	5	5	4
4	4.24	4	4	4	4	4	4	4
5	4.76	5	5	5	5	5	5	4
4	4.21	4	4	4	4	4	4	4
5	3.48	4	4	4	4	5	5	4
4	3.52	5	5	5	5	5	4	4
5	4.15	5	4	4	5	5	4	5
3	3.48	4	4	4	4	4	4	4
3	3.70	4	4	3	4	3	4	3
2	3.21	3	4	4	4	4	3	4
3	4.09	5	5	4	4	5	5	5
4	3.76	4	4	4	4	4	4	4

5	3.88	3	2	4	4	4	5	5
5	4.18	4	4	4	4	4	4	4
5	3.55	4	4	4	4	4	4	4
4	4.00	4	4	5	4	4	4	5
3	3.85	4	4	4	4	4	4	4
4	4.00	4	4	4	4	4	4	4
5	4.24	5	5	5	5	4	5	5
4	3.85	5	4	5	4	4	5	4
5	4.64	4	4	4	4	4	4	4
5	4.06	4	4	4	3	4	4	3
3	3.67	4	4	4	4	4	4	4
4	3.73	3	3	4	4	4	3	4
3	4.12	4	4	4	4	3	3	4
4	4.06	4	5	5	5	5	4	4
4	4.09	3	3	4	4	2	4	4
4	3.42	4	4	4	4	4	4	3
4	4.03	4	4	4	4	4	4	4
4	3.33	4	4	4	4	4	5	5
4	4.12	4	4	4	4	3	4	4
4	3.94	5	5	5	5	5	5	5
4	4.12	4	4	5	4	4	4	4
4	4.12	4	4	4	4	4	4	5
5	4.45	5	5	5	5	5	5	5
5	4.58	4	4	4	4	4	4	4
5	4.30	5	4	4	4	5	4	4
3	3.76	4	4	4	4	4	4	4
4	4.03	4	4	4	5	4	4	4
4	4.27	5	5	5	5	5	5	4
4	3.73	5	5	4	4	4	4	4
4	3.79	4	4	4	3	3	4	2
5	3.97	5	4	4	4	4	4	5
5	4.36	5	4	5	4	4	5	5
5	4.52	4	4	4	4	4	4	4
4	4.18	4	4	5	5	5	5	4
4	3.73	5	5	4	4	4	4	4
3	3.85	4	4	4	4	4	4	4
5	5.00	5	5	5	5	5	5	5
5	4.06	4	4	4	4	5	4	4
5	4.03	4	4	4	4	4	4	4
3	3.39	3	3	2	4	3	5	3
4	4.27	4	4	4	4	4	4	4
3	3.64	4	5	4	4	4	3	4
5	3.91	4	4	4	4	4	4	4
4	3.94	4	4	4	4	4	4	4
5	4.55	4	4	5	5	5	4	4
5	5.00	5	5	5	5	5	5	5
4	3.58	3	4	4	3	4	4	4
4	3.85	4	4	4	4	4	4	3
4	4.00	5	5	5	5	5	5	5
4	3.67	4	4	4	4	5	4	5
5	3.21	4	5	4	5	4	5	4
5	4.00	5	5	5	5	5	5	5
5	4.79	5	5	5	5	5	5	5
3	3.76	3	3	3	4	3	4	4

4	4.00	4	4	4	4	4	4	4
4	4.18	4	4	5	4	4	5	4
4	4.24	5	5	4	4	4	5	4
4	3.52	5	5	5	5	5	5	5
4	3.82	3	3	4	3	3	3	4
4	4.00	4	4	4	4	4	4	4
5	4.70	5	5	4	5	4	5	4
4	3.88	5	4	5	5	5	5	5
4	4.15	4	4	4	4	5	4	5
4	4.15	4	4	4	4	4	4	5
4	4.03	5	5	5	5	5	5	4
4	3.67	5	4	4	4	5	5	4
4	3.45	4	3	4	5	4	3	4
4	4.15	4	5	4	5	4	5	4
5	4.91	4	5	4	4	5	5	4
3	3.73	4	4	5	4	4	4	4
3	3.73	4	4	4	4	4	4	4
5	4.36	4	4	4	4	4	4	4
5	4.18	4	4	5	4	4	4	4
3	3.58	4	4	4	4	3	4	4
4	3.76	4	4	4	4	4	4	4
3	3.48	3	3	3	3	4	3	4
4	3.21	4	4	4	4	3	4	3
3	3.06	4	5	5	5	5	5	5
4	3.61	4	4	4	3	3	4	4
4	3.58	4	5	4	4	3	4	4
4	4.03	4	4	4	5	5	4	4
4	4.00	4	4	4	4	4	4	4
5	4.21	5	4	4	4	4	5	4
5	4.39	4	5	5	5	5	5	5
4	4.06	4	4	4	5	4	5	4
5	4.12	4	4	4	4	4	4	4
5	4.64	4	4	4	5	5	4	4
4	3.82	4	4	4	4	4	4	4
5	3.61	3	4	3	4	3	4	3
5	4.27	4	3	5	5	3	3	5
4	3.85	3	3	3	4	4	3	3
4	3.91	4	4	4	4	4	4	4
4	4.06	5	5	5	5	5	5	5
4	3.33	4	4	4	4	4	4	4
2	3.15	1	2	3	4	5	4	3
5	4.18	4	4	4	4	4	4	4
4	3.94	4	4	4	4	4	4	4
4	3.64	4	4	4	4	4	4	4
4	4.09	4	4	4	4	4	4	4
5	4.09	4	4	3	3	3	4	4
5	4.33	4	4	4	4	4	4	4
5	4.48	5	5	5	5	5	5	5
4	4.55	4	4	4	4	4	4	5
5	4.55	4	5	5	4	4	4	5
5	4.73	4	5	5	5	5	5	5
4	3.67	4	5	5	5	4	5	4
4	3.82	5	5	5	5	5	5	3
5	4.48	4	4	4	5	5	5	5

4	3.91	4	5	5	4	5	5	4
5	4.91	5	5	5	5	5	5	5
3	3.06	4	5	5	4	4	4	4
4	3.03	4	5	4	4	4	5	5
4	4.12	4	4	4	4	4	4	4
4	3.52	4	4	4	4	4	4	4
4	3.55	4	4	4	4	4	4	4
5	3.88	4	4	4	4	3	3	3
4	3.94	4	4	4	4	4	4	4
4	3.85	4	4	4	4	4	3	4
3	3.39	3	3	3	3	3	4	4
3	4.52	5	5	5	5	4	5	5
3	3.18	4	4	4	4	4	4	4
4	3.73	4	4	4	4	3	3	3
4	3.73	4	4	4	4	4	4	4
4	3.79	4	4	4	4	4	4	4
4	3.79	4	4	4	4	4	4	4
5	4.00	4	4	4	4	4	4	3
4	3.73	4	4	4	4	4	4	4
4	3.79	4	4	4	4	4	4	4
4	3.79	4	4	4	4	4	4	4
4	2.85	2	3	3	4	4	3	4
3	2.64	4	4	4	4	4	4	4
5	3.52	4	5	5	5	5	4	5
4	3.64	5	5	5	5	5	5	3
3	3.61	4	4	4	4	4	4	3
4	4.58	5	5	5	5	5	4	4
4	3.97	4	4	5	4	4	4	4
4	4.18	4	4	4	3	4	4	4
5	3.70	4	4	4	4	4	4	4
4	3.64	4	4	4	4	3	4	4
3	3.00	4	4	4	4	4	4	4
5	3.67	4	4	4	4	4	4	4
5	4.15	4	4	4	5	5	4	5
4	3.73	3	4	3	4	3	4	3
3	3.18	4	3	3	4	3	4	3
4	3.27	4	4	4	4	4	4	4
5	4.45	4	5	5	5	5	5	4
5	4.52	5	5	5	5	5	5	5
3	3.33	3	3	3	4	3	4	3
3	3.82	3	3	3	3	3	3	3
4	4.00	4	4	4	4	4	4	4
5	4.58	5	5	5	5	5	5	5
3	3.67	4	3	4	4	4	4	4
4	4.00	4	4	4	4	4	4	4
4	3.27	4	4	4	4	4	4	4
4	4.03	3	5	4	4	4	4	4
4	3.91	5	4	5	4	5	4	5
3	3.09	3	3	3	3	3	3	3
4	3.18	3	3	4	4	4	4	4
3	3.00	3	3	3	3	3	3	3
5	4.88	2	5	5	5	5	5	5
5	4.55	5	5	5	5	5	5	5
4	3.48	4	4	4	3	3	3	3

4	4.00	5	5	5	5	5	5	5
4	4.79	5	5	5	5	5	5	5
4	4.73	4	4	4	4	5	4	4
5	5.00	5	5	5	5	5	5	5
4	3.58	4	4	4	5	4	4	4
5	4.15	5	4	4	4	5	4	4
4	4.00	4	4	4	4	4	4	4
4	4.00	4	4	4	4	4	4	4
3	3.48	4	4	4	3	4	4	4
3	3.30	4	3	3	4	3	4	3
4	4.12	2	4	4	4	4	4	4
4	4.42	4	4	4	4	4	4	4
5	4.27	4	4	4	4	4	4	4
3	3.58	5	5	4	5	3	5	5
5	4.42	5	5	5	5	5	5	5
3	3.42	3	4	3	3	3	3	3
4	3.24	4	4	4	4	4	4	4
5	3.97	5	5	4	4	4	4	4
4	3.94	4	4	4	5	4	4	5
4	3.52	4	4	4	4	4	4	4
4	3.70	3	5	3	4	4	4	5
5	4.48	5	5	4	5	5	5	4
4	4.03	3	4	4	4	4	3	4
5	2.91	3	2	5	1	5	1	4
4	3.76	3	4	4	4	4	4	4
4	4.61	4	4	4	4	4	4	4
3	3.24	3	2	4	4	3	4	3
4	4.09	4	4	5	4	5	4	4
4	4.48	5	5	5	5	5	5	5
4	3.94	4	4	4	4	4	4	5

B8	B9	B10	B11	B12	B13	B14	B15	teaching efficacy(B)
4	4	4	4	4	4	4	4	3.93
2	3	3	4	3	2	4	2	2.80
4	5	4	3	1	2	3	4	3.33
4	5	4	3	1	2	3	4	3.33
4	5	4	3	1	2	3	4	3.33
4	5	5	4	2	3	4	3	3.87
5	4	3	1	2	3	4	3	3.33
3	4	4	4	4	4	4	3	3.67
4	3	4	4	4	5	4	5	3.87
3	3	4	4	3	3	4	3	3.47
4	4	5	4	5	4	5	5	4.47
4	5	5	5	5	5	5	4	4.80
4	4	4	4	4	4	4	4	4.00
4	4	4	4	4	4	4	4	4.00
4	4	4	4	4	4	4	4	4.00
3	4	5	4	4	3	4	5	4.13
4	4	4	4	5	4	4	4	4.07
4	5	5	4	4	5	4	3	4.27
4	3	5	5	4	3	4	5	4.20
4	4	4	4	4	4	5	4	4.33
4	4	4	4	4	5	4	4	4.07
3	5	3	5	4	4	4	4	4.07
5	5	5	5	5	5	5	5	4.80
3	3	4	4	4	4	4	4	3.93
5	3	3	4	4	4	4	4	3.93
4	4	4	5	4	4	4	4	4.00
5	4	4	4	4	3	4	3	4.00
5	5	5	5	5	5	4	5	4.87
5	5	4	5	5	4	4	4	4.27
4	4	4	5	4	3	3	4	3.60
4	5	4	5	4	5	4	5	4.40
5	5	4	4	4	5	5	4	4.33
4	5	4	4	4	4	5	5	4.40
5	4	4	3	4	4	3	4	4.00
4	4	4	4	4	4	4	4	4.47
4	4	5	5	4	4	5	4	4.27
4	3	4	3	4	4	4	4	3.80
4	4	4	4	3	4	3	4	3.87
4	5	5	4	4	4	5	4	4.33
3	5	5	5	5	5	5	4	4.73
4	4	4	4	4	4	5	5	4.20
2	4	2	4	2	3	4	3	3.00
4	5	5	5	5	5	5	5	4.93
4	5	4	3	5	4	4	4	4.13
3	3	3	3	3	3	3	3	3.13
5	4	4	5	5	4	4	5	4.47
5	5	5	5	4	4	4	4	4.27
4	5	5	4	5	4	5	4	4.47
4	4	5	5	5	5	4	4	4.47
5	4	4	3	4	4	4	4	3.93
4	5	5	4	5	4	5	5	4.60

4	5	4	4	5	5	5	5	4.33
4	4	5	5	5	4	4	4	4.40
4	4	4	5	4	4	5	5	4.47
5	4	4	4	5	4	4	3	4.07
4	3	3	3	4	4	4	3	3.53
4	5	5	4	3	3	4	5	4.13
4	4	5	5	4	4	5	4	4.40
4	4	5	5	4	5	4	5	4.47
4	4	5	4	4	4	4	4	4.07
4	4	4	5	5	4	4	4	4.53
4	4	4	4	4	4	4	4	4.00
3	3	3	2	2	2	3	2	2.60
4	4	4	3	3	3	4	4	3.73
5	4	2	3	4	3	4	2	3.33
5	5	5	5	5	5	5	5	5.00
4	4	4	4	4	4	4	4	4.00
5	5	5	5	5	4	5	4	4.87
5	5	5	5	5	5	5	5	5.00
4	4	4	4	4	4	4	4	4.00
3	4	3	3	3	3	4	4	3.60
5	5	5	4	5	5	5	5	4.93
4	4	5	5	4	4	5	4	4.47
4	3	3	3	3	3	3	3	3.33
4	4	4	4	4	4	3	3	3.80
3	3	3	3	3	3	3	3	3.00
4	4	4	4	4	4	4	4	4.00
4	5	5	4	5	5	5	5	4.80
4	4	4	4	4	4	4	4	3.87
3	3	4	4	4	4	4	3	3.73
4	4	4	5	5	5	3	4	4.13
4	5	5	4	5	5	5	5	4.40
4	4	4	4	4	5	5	4	4.00
4	4	4	4	4	4	4	4	4.00
4	3	5	5	3	5	3	5	3.87
4	4	4	4	4	4	4	4	4.00
4	4	4	4	4	4	4	4	4.00
4	4	4	4	4	4	4	4	4.00
4	4	4	4	4	4	4	4	3.93
4	4	4	4	4	4	4	4	4.00
4	4	4	4	5	5	5	5	4.40
5	4	4	4	5	5	4	5	4.53
3	4	4	3	4	4	4	4	3.80
3	4	4	3	4	4	4	4	3.80
4	5	4	4	3	4	4	4	4.00
4	5	4	5	4	5	4	5	4.33
4	5	4	5	5	5	5	5	4.53
3	4	4	5	5	4	4	4	4.13
4	4	4	4	4	4	4	4	4.00
3	4	4	4	4	4	4	4	3.87
4	4	4	4	4	5	5	5	4.20
5	5	4	4	4	5	5	5	4.47
5	5	4	4	5	4	5	4	4.47
5	5	4	4	5	4	5	4	4.47
4	4	4	4	5	4	5	5	4.07

4	4	4	4	4	4	4	4	4.00
4	4	4	4	4	4	4	4	3.87
5	5	4	4	5	5	5	4	4.67
3	3	3	4	3	3	3	3	3.20
4	4	4	4	4	4	4	4	4.07
4	4	5	4	5	4	4	5	4.00
4	3	4	4	4	4	4	4	3.93
4	4	4	4	4	4	4	4	3.93
4	4	4	4	4	4	4	4	4.00
5	5	4	4	4	4	4	4	4.33
5	5	5	5	5	5	5	5	5.00
4	4	4	4	4	4	4	4	4.00
3	3	4	4	4	3	3	3	3.40
4	4	4	4	4	4	4	4	3.87
4	4	4	4	4	4	4	4	4.00
4	3	3	2	3	3	4	3	3.13
5	5	5	5	4	4	4	4	4.73
5	4	4	5	4	5	4	5	4.47
5	4	5	4	4	4	5	5	4.53
4	4	3	3	4	4	4	4	3.73
5	5	4	4	4	5	4	5	4.47
4	4	4	4	4	4	5	5	4.13
4	5	4	3	2	1	2	3	2.93
4	3	4	4	4	3	4	4	3.73
4	4	4	4	4	4	4	4	4.27
5	5	5	5	4	4	4	4	4.47
4	4	5	5	4	3	5	4	4.40
1	3	3	2	2	3	5	4	2.73
5	4	4	4	4	5	4	5	4.47
3	4	3	4	4	4	4	4	3.67
4	5	5	5	5	5	5	5	4.80
5	4	4	3	5	5	5	5	4.73
5	5	4	4	5	5	5	5	4.67
5	4	3	4	5	4	4	5	4.20
4	4	4	5	4	5	4	5	4.40
3	4	3	3	4	4	2	3	3.07
4	4	3	3	4	4	3	3	3.60
3	3	3	4	4	4	4	3	3.47
4	5	5	5	5	5	4	4	4.53
4	4	4	3	3	4	3	4	3.40
4	3	3	3	2	3	4	4	3.60
4	5	5	5	5	5	5	4	4.60
4	5	5	5	5	5	5	4	4.60
4	4	4	4	4	4	4	4	4.00
3	5	4	5	5	3	3	3	4.33
4	5	4	5	5	5	4	4	4.27
5	4	3	4	5	4	3	4	4.13
4	4	4	4	5	5	5	4	4.53
3	3	4	5	5	4	5	4	4.33
3	4	4	4	4	4	4	4	3.93
4	3	4	3	4	3	4	3	3.53
3	4	4	4	2	3	3	3	3.47
4	5	5	4	4	5	5	5	4.67
4	4	4	4	4	4	4	4	4.00

4	4	2	3	2	3	1	4	3.33
4	4	4	4	4	4	4	4	4.00
3	4	4	4	4	4	3	4	3.87
4	4	5	4	4	4	5	4	4.27
4	4	4	4	4	4	4	4	4.00
4	4	4	4	4	4	4	4	4.00
5	4	5	5	5	5	5	5	4.87
5	4	5	4	5	5	4	5	4.53
4	4	4	4	4	4	4	4	4.00
4	4	5	3	4	4	4	4	3.87
4	4	4	4	4	4	4	4	4.00
4	4	4	4	4	4	4	4	3.80
4	3	3	4	4	4	4	4	3.73
4	5	5	5	5	5	5	5	4.73
3	5	5	5	5	4	5	3	3.93
2	4	2	2	2	2	2	2	3.00
4	4	4	4	4	4	4	4	4.00
4	4	4	4	4	4	4	4	4.13
4	4	4	3	4	3	4	4	3.80
5	5	5	5	5	5	5	5	5.00
4	5	4	4	4	4	4	4	4.13
5	4	4	4	4	4	4	4	4.13
5	5	5	5	5	5	5	5	5.00
4	4	5	5	5	5	5	5	4.40
4	5	5	5	5	5	5	5	4.60
3	4	4	3	4	3	4	4	3.80
4	4	4	4	4	4	4	4	4.07
5	5	5	5	5	5	5	5	4.93
4	4	4	4	4	4	4	4	4.13
3	4	5	5	5	5	5	4	4.00
5	5	4	5	5	5	4	5	4.53
5	5	4	5	5	5	5	5	4.73
4	5	4	4	4	4	4	4	4.07
4	4	4	4	4	4	4	4	4.27
4	4	4	4	4	4	4	4	4.13
4	4	5	4	4	4	5	4	4.13
5	5	5	5	5	5	5	5	5.00
5	5	5	4	4	5	5	5	4.47
3	4	4	4	4	4	4	4	3.93
4	3	5	3	4	4	3	4	3.53
4	4	5	5	4	5	4	5	4.27
4	4	5	4	4	4	4	4	4.07
4	4	5	4	4	4	4	4	4.07
4	4	4	4	4	4	4	4	4.00
4	4	4	5	4	4	4	4	4.27
5	5	5	5	5	5	5	5	5.00
3	4	3	4	3	3	4	3	3.53
4	4	4	3	4	4	4	4	3.87
5	5	5	5	5	5	5	5	5.00
5	5	5	4	5	5	4	5	4.53
5	4	5	4	5	4	5	4	4.47
5	5	5	5	5	5	5	5	5.00
5	5	5	5	5	5	5	5	5.00
4	4	4	3	3	3	3	4	3.47

4	4	4	4	4	4	4	4	4.00
4	5	4	4	4	4	4	4	4.20
4	4	4	4	4	4	5	5	4.33
5	5	5	5	5	5	5	5	5.00
3	3	3	4	3	3	3	3	3.20
4	4	4	4	4	4	4	4	4.00
5	5	5	5	5	5	5	5	4.80
5	4	5	5	5	4	5	5	4.80
4	4	4	4	4	4	4	4	4.13
5	5	5	4	4	4	5	5	4.40
4	4	4	5	4	5	5	5	4.67
4	4	4	4	4	4	4	4	4.20
4	3	2	3	4	3	4	4	3.60
5	5	4	4	5	5	4	5	4.53
4	4	4	5	5	5	5	4	4.47
4	5	5	4	4	4	5	4	4.27
4	4	4	4	3	4	4	4	3.93
4	4	4	4	4	4	4	4	4.00
4	4	4	4	4	4	4	4	4.07
4	4	4	4	4	4	4	4	3.93
4	4	4	4	4	4	4	4	4.00
3	4	3	4	4	4	4	3	3.47
4	4	5	4	5	4	3	4	3.93
5	5	5	5	5	4	4	4	4.73
5	4	4	4	4	4	4	4	3.93
3	4	4	4	5	4	4	4	4.00
4	4	5	5	4	4	4	4	4.27
4	4	4	4	4	4	4	4	4.00
4	5	4	4	5	4	4	5	4.33
5	5	5	5	5	5	5	5	4.93
5	4	5	4	5	4	5	5	4.47
4	4	4	4	4	4	4	4	4.00
4	5	5	5	4	5	5	5	4.53
4	4	4	4	4	4	4	4	4.00
4	3	4	3	4	3	4	3	3.47
5	5	5	2	2	5	5	5	4.13
3	3	5	4	4	3	3	3	3.40
4	4	4	4	4	4	4	4	4.00
5	5	5	5	5	4	4	4	4.80
4	4	4	4	4	4	4	4	4.00
2	2	3	4	5	4	3	2	3.13
4	4	4	4	4	4	4	4	4.00
4	4	4	4	4	4	4	4	4.00
4	4	3	4	4	4	4	4	3.93
4	5	4	5	4	4	4	5	4.20
3	4	3	4	4	4	3	4	3.60
4	4	3	4	4	3	3	4	3.80
4	5	5	5	5	5	5	4	4.87
5	4	5	4	5	4	5	4	4.33
4	4	4	4	4	5	5	4	4.33
4	4	5	5	5	5	5	2	4.60
5	4	5	5	5	5	5	5	4.73
4	5	5	5	5	5	5	5	4.80
5	5	5	5	5	5	4	4	4.67

5	4	5	4	4	4	4	4	4.40
5	5	5	5	5	5	5	5	5.00
5	5	4	4	4	5	4	5	4.40
5	4	3	3	4	4	4	5	4.20
4	4	4	4	4	4	4	4	4.00
4	4	4	4	4	4	4	4	4.00
4	4	4	4	4	4	4	4	4.00
3	3	3	4	4	3	3	4	3.47
4	4	4	4	4	4	4	4	4.00
3	4	4	4	4	4	4	4	3.87
4	4	4	4	4	4	4	3	3.60
5	5	5	5	5	5	5	4	4.87
5	4	4	4	4	4	4	4	4.07
3	3	3	4	4	4	4	4	3.60
4	4	4	4	4	4	4	4	4.00
4	4	4	4	4	4	4	4	4.00
4	4	4	4	4	4	4	4	4.00
3	4	4	4	4	3	4	3	3.73
4	4	4	4	4	4	4	4	4.00
4	4	4	4	4	4	4	4	4.00
4	4	4	4	4	4	4	4	4.00
3	4	4	4	3	4	4	3	3.47
4	4	4	4	4	4	4	4	4.00
4	5	4	4	5	4	5	4	4.53
4	5	5	4	4	4	3	3	4.33
3	4	4	4	4	4	4	4	3.87
4	4	4	4	5	5	5	5	4.60
5	4	4	5	4	4	4	5	4.27
4	4	4	4	4	4	4	4	3.93
4	4	4	4	4	4	4	4	4.00
4	4	4	4	4	4	4	4	3.93
4	4	4	4	4	3	3	3	3.80
4	4	2	4	4	5	3	4	3.87
5	5	5	5	5	5	5	5	4.73
4	3	4	4	4	4	4	4	3.67
4	3	4	3	4	4	3	4	3.53
4	4	4	4	4	4	4	4	4.00
5	4	5	4	5	5	4	5	4.67
5	5	5	5	5	5	5	5	5.00
4	3	3	4	3	3	3	3	3.27
3	3	3	3	3	4	4	4	3.20
4	4	4	4	4	4	4	4	4.00
5	4	4	4	4	4	4	4	4.53
4	4	4	5	4	4	4	4	4.00
4	4	4	4	4	4	4	4	4.00
4	4	4	4	4	5	5	5	4.20
4	4	4	4	4	5	5	3	4.07
4	5	4	4	5	4	4	4	4.40
3	3	3	3	3	5	5	5	3.40
4	4	4	3	4	4	4	4	3.80
3	3	3	3	3	3	3	3	3.00
3	2	3	5	5	5	5	5	4.33
5	5	5	5	5	5	3	3	4.73
3	4	4	4	4	4	3	3	3.53

5	5	5	5	5	4	4	4	4.80
4	2	5	5	1	5	4	5	4.40
4	4	5	5	4	4	4	5	4.27
5	5	5	5	5	4	5	5	4.93
4	4	4	4	4	5	5	4	4.20
3	3	3	4	4	5	5	4	4.07
2	2	2	3	4	4	4	4	3.53
4	4	4	4	4	4	4	4	4.00
4	4	3	2	4	4	3	3	3.60
3	3	3	4	4	4	3	3	3.40
2	2	2	3	2	4	4	4	3.27
4	4	4	4	4	5	4	4	4.07
4	4	4	4	4	4	4	4	4.00
4	5	5	4	4	5	5	5	4.60
4	4	3	4	4	5	5	5	4.60
3	4	4	4	4	4	3	4	3.47
4	4	4	4	5	5	5	5	4.27
4	4	4	4	4	4	4	4	4.13
4	3	4	4	2	5	5	5	4.13
4	4	4	4	4	4	4	4	4.00
5	4	4	5	5	3	3	4	4.07
4	5	4	5	5	5	4	5	4.67
3	2	2	2	4	5	3	2	3.27
1	3	3	5	1	2	2	3	2.73
4	4	3	4	3	4	4	4	3.80
4	4	4	4	4	4	4	4	4.00
4	3	4	3	3	5	5	5	3.67
4	4	4	4	4	4	4	5	4.20
5	5	5	5	5	4	5	5	4.93
3	3	2	3	4	5	4	4	3.80

C1	C2	C3	C4	C5	C6	C7	C8	C9
5	5	5	5	5	5	5	5	5
1	3	2	4	3	2	4	3	2
5	3	4	5	5	5	4	3	4
5	4	4	5	5	5	4	3	4
5	4	4	5	5	5	4	3	4
5	3	4	5	5	5	4	4	3
5	3	4	5	5	5	5	4	3
4	4	3	4	4	3	3	4	4
4	4	2	4	4	3	4	4	4
5	4	3	4	4	4	4	4	4
4	5	5	4	5	5	4	5	4
5	1	4	5	5	5	5	5	5
4	3	3	4	4	3	3	4	4
4	4	4	4	4	4	5	5	5
4	4	4	4	4	4	5	5	5
4	4	3	4	5	4	4	5	4
5	4	4	5	4	4	4	4	4
5	5	5	4	4	4	2	2	4
5	4	3	4	5	4	5	4	4
4	4	3	5	5	4	4	4	5
5	5	3	4	4	4	4	5	5
5	1	3	4	4	5	5	5	2
5	5	4	5	5	5	5	5	5
4	4	5	5	5	5	5	5	5
4	4	4	4	5	5	5	5	5
4	3	4	4	4	3	4	4	3
5	5	1	5	4	3	3	5	5
5	2	4	5	5	5	5	5	5
5	4	3	5	5	5	5	3	5
3	4	5	2	3	4	3	4	4
4	5	4	4	4	5	5	5	5
3	4	5	4	4	4	4	5	5
4	5	4	4	5	5	5	4	4
3	4	5	4	4	4	3	3	4
5	5	1	5	5	5	2	1	5
5	4	5	4	4	4	5	4	4
4	4	4	4	4	4	4	4	4
5	4	3	4	3	4	4	4	4
5	4	2	4	2	3	3	4	5
5	4	5	5	5	5	5	5	5
5	5	5	5	5	5	5	4	4
3	2	4	3	2	4	2	3	4
5	4	4	5	5	5	5	5	5
5	4	5	4	4	5	5	4	5
4	4	4	3	3	3	3	3	3
5	5	5	5	5	5	4	4	4
5	5	4	4	5	4	5	5	5
4	5	5	5	5	4	4	4	4
4	4	4	5	5	4	4	4	5
4	4	4	4	5	5	5	5	3
5	5	5	4	4	4	4	4	5

4	4	4	4	4	4	4	4	4
4	4	4	5	4	5	5	5	4
4	5	4	5	4	5	5	5	5
4	4	5	4	4	3	4	4	5
3	3	3	4	4	4	3	3	3
4	5	4	4	5	4	3	4	5
4	4	5	5	4	4	4	5	5
4	4	4	5	5	4	4	4	5
5	5	3	5	3	4	4	4	4
5	5	2	5	5	5	5	5	3
4	4	4	4	4	4	4	4	4
3	3	2	2	2	3	3	3	3
4	2	2	4	3	2	3	2	4
4	3	4	2	4	3	4	2	5
5	4	3	5	5	5	5	5	5
5	5	5	5	5	5	5	5	5
5	5	5	5	5	4	4	5	5
5	4	4	5	5	5	5	5	5
5	3	3	5	3	3	3	3	5
4	4	4	4	4	3	4	4	4
4	4	5	5	5	4	5	5	5
5	4	5	5	5	5	5	5	5
4	4	4	4	4	3	4	4	4
5	3	3	5	5	4	5	4	5
3	3	3	3	4	4	4	3	4
5	2	4	4	4	4	4	3	4
5	5	5	5	5	5	5	5	5
4	4	4	4	3	4	3	4	4
4	4	3	4	3	3	3	4	4
5	5	5	5	4	4	4	5	5
5	4	4	4	4	4	4	4	4
5	4	3	5	4	4	4	4	5
5	4	4	5	4	5	5	5	5
5	4	3	5	4	4	4	5	5
4	4	4	4	4	4	4	4	4
4	4	4	4	4	4	5	5	5
3	3	3	5	5	5	5	5	5
4	4	4	4	4	4	5	4	4
4	4	4	4	4	4	4	4	4
5	5	5	5	5	5	5	5	5
4	4	4	4	4	4	4	5	4
5	5	4	5	5	5	5	5	5
5	5	4	5	5	5	5	5	5
4	4	5	4	4	3	4	4	5
5	4	5	5	4	5	4	5	4
4	4	2	5	4	4	4	4	5
5	3	3	4	4	4	4	5	5
5	4	3	5	4	4	4	4	4
5	4	3	4	4	4	3	4	4
5	4	4	5	4	4	4	4	4
4	4	5	4	5	4	4	4	5
5	5	4	4	5	5	4	4	5
5	5	4	4	5	5	4	4	5
5	4	3	5	4	4	4	4	5

4	3	4	4	4	4	4	4	4
4	3	3	5	3	3	4	4	4
5	5	5	5	5	5	5	5	5
3	3	3	4	4	4	3	3	3
5	4	2	5	5	5	4	4	4
3	3	3	4	4	4	4	5	4
4	4	4	4	3	3	4	4	4
4	3	3	5	4	4	4	4	5
4	3	3	5	4	4	4	4	5
5	4	5	5	5	5	5	5	5
5	5	5	5	5	5	5	4	4
4	3	3	5	4	4	4	4	5
3	3	3	4	4	4	3	3	3
4	4	2	4	4	4	3	4	4
4	4	1	4	4	3	4	4	4
3	2	2	3	2	3	3	3	4
5	5	5	5	5	5	5	5	5
4	4	4	5	4	5	4	4	5
4	4	3	4	4	4	5	5	5
4	4	4	5	5	4	4	4	5
5	4	5	4	5	4	4	4	5
4	4	4	4	4	4	5	5	5
5	4	3	1	2	3	4	5	4
4	4	4	4	4	4	4	4	4
5	5	5	5	5	4	4	4	4
4	3	4	5	5	5	5	5	5
5	3	4	4	3	4	4	5	5
2	3	4	4	3	3	4	4	3
5	5	4	4	4	4	4	4	4
4	3	4	4	4	4	4	4	4
5	4	4	5	5	5	5	5	5
5	5	5	4	4	5	5	5	4
5	5	4	4	5	4	4	5	4
4	5	5	5	4	4	5	5	5
4	5	4	5	5	4	4	4	5
4	4	3	4	2	3	3	4	4
4	4	4	4	4	4	4	4	4
3	3	3	4	4	4	3	4	4
4	4	5	4	4	4	5	5	4
4	4	4	4	4	4	4	4	4
5	4	3	5	5	4	4	5	4
5	4	4	5	4	4	4	5	5
5	4	4	5	4	4	4	5	5
4	4	3	4	3	3	3	3	4
5	5	5	5	5	5	5	5	5
5	3	4	5	4	5	4	5	4
4	4	4	4	3	4	5	4	3
4	2	4	5	5	5	5	5	5
4	4	4	5	5	5	5	5	5
4	4	4	4	4	4	4	4	4
4	4	4	4	4	4	4	4	4
4	1	3	5	5	5	5	5	5
4	3	4	4	4	4	4	4	4
4	4	4	4	4	4	4	4	4

3	4	4	5	5	4	2	3	2
3	3	3	4	4	4	4	4	4
4	4	4	4	4	3	4	4	4
4	2	4	4	5	4	4	4	5
5	4	4	5	3	3	3	5	4
5	4	5	5	5	5	3	5	5
5	5	3	5	3	1	3	4	4
4	4	3	4	4	4	5	5	4
5	4	4	5	4	4	4	4	4
5	5	3	5	1	4	3	2	1
4	4	4	4	4	4	4	4	4
4	4	4	4	4	4	4	4	4
4	4	4	4	3	3	4	4	5
5	5	5	5	5	5	5	5	5
5	3	3	5	3	4	4	4	5
4	4	4	4	4	4	4	4	4
5	5	5	5	5	5	5	5	5
4	4	5	5	5	5	5	5	5
4	4	3	5	4	4	4	4	5
5	5	4	5	5	5	4	4	4
5	4	5	4	4	4	5	5	5
4	5	4	4	4	4	4	4	4
5	5	5	5	5	5	5	5	5
4	4	4	4	4	4	4	4	4
5	5	5	5	5	5	5	5	5
4	3	3	4	3	3	4	4	4
5	5	4	4	4	4	4	4	5
5	3	3	5	5	5	5	5	5
5	5	5	5	4	4	4	4	4
5	4	3	5	4	4	4	5	5
5	4	3	5	4	4	4	5	5
5	5	3	5	4	4	4	5	5
5	5	5	5	5	5	5	5	5
4	4	4	5	4	4	5	4	5
5	5	5	5	4	4	4	4	4
5	4	3	5	3	3	3	3	5
5	5	5	5	5	5	5	5	5
5	4	4	5	4	5	4	3	4
4	4	4	4	4	4	4	4	4
3	4	3	4	3	4	5	4	5
5	5	5	4	4	4	5	5	4
4	5	4	4	4	5	3	4	3
4	3	3	4	4	4	4	4	5
5	4	4	5	4	4	4	4	4
5	4	4	4	4	4	4	4	4
5	5	5	5	5	5	5	5	5
4	4	3	5	3	4	4	5	4
4	3	4	4	4	4	4	4	4
4	4	4	5	5	5	4	4	4
4	4	4	4	5	4	4	4	4
4	4	4	5	4	5	4	5	4
5	5	5	5	5	4	5	5	5
5	5	5	5	5	4	5	5	5
5	5	3	4	4	4	5	5	4

4	4	4	4	4	4	4	4	4
4	4	3	4	4	5	4	5	4
4	4	4	5	4	5	5	5	5
3	1	5	5	5	5	5	5	5
4	3	2	4	3	2	3	2	5
4	4	4	4	4	4	4	4	4
5	5	5	5	5	5	5	5	5
5	5	5	5	5	5	4	5	5
4	4	4	4	4	4	4	4	4
5	5	5	5	4	5	3	3	4
5	4	4	5	5	5	5	5	5
4	4	4	4	4	4	5	4	4
4	3	4	4	4	4	3	4	4
5	5	5	4	4	5	5	5	4
5	5	5	5	5	5	5	5	5
5	4	3	5	5	4	4	5	5
5	4	4	5	4	4	4	4	5
4	4	4	4	4	4	4	4	4
4	3	3	5	4	3	4	4	5
4	4	4	4	3	3	4	4	3
4	4	4	4	4	4	4	4	4
3	4	4	4	4	3	4	4	4
4	3	4	4	5	3	5	4	4
5	4	4	4	4	4	4	4	4
4	4	3	4	3	3	3	3	4
4	4	5	5	4	3	4	5	4
4	4	4	5	5	4	4	4	4
4	4	4	4	4	4	4	4	4
5	5	5	5	5	5	5	5	5
4	4	5	4	5	5	5	4	4
4	4	4	5	4	5	4	5	4
5	5	2	4	4	3	3	3	4
4	4	5	4	4	4	4	4	4
4	4	3	4	4	4	3	3	4
3	4	3	4	3	4	3	4	3
5	3	5	5	5	5	5	5	5
4	4	4	4	5	4	4	4	4
5	4	4	4	4	4	4	4	4
5	5	5	5	5	5	5	5	5
4	4	4	4	4	4	4	4	4
1	2	3	4	5	4	4	3	2
4	4	4	4	4	4	4	4	4
5	3	3	4	4	4	4	4	4
4	4	3	5	4	4	4	4	4
5	5	4	5	5	5	4	5	5
5	4	4	4	5	5	4	4	5
4	4	4	4	4	3	3	4	4
5	4	4	5	5	5	5	5	5
5	5	5	5	5	5	5	5	5
5	5	5	5	5	5	5	5	5
5	3	3	5	5	5	3	2	5
4	4	4	5	4	4	4	5	4
5	3	5	5	5	5	5	5	5
4	4	4	4	4	4	4	4	5

4	5	5	5	4	4	4	4	4
5	4	5	5	5	5	5	5	5
4	5	5	5	5	5	4	5	5
5	4	5	4	5	5	4	3	4
5	5	4	5	4	4	4	4	4
4	4	4	4	4	4	4	4	4
3	3	3	4	4	4	4	4	4
5	5	5	4	4	4	5	4	4
5	4	5	5	5	5	5	5	5
4	4	4	4	4	4	4	4	4
5	5	5	5	4	4	5	5	5
5	5	5	5	5	5	5	5	5
4	4	4	4	4	4	5	5	5
4	4	4	4	4	5	5	5	4
4	4	4	4	4	4	4	4	4
4	4	5	4	4	3	4	4	5
4	4	4	4	4	4	4	4	4
5	5	5	5	4	5	5	4	4
4	4	4	4	4	4	4	4	4
4	4	5	4	4	3	4	4	5
4	4	4	4	4	4	4	4	4
2	3	3	4	4	3	4	3	4
4	4	4	4	4	4	4	4	4
5	4	5	4	4	4	4	4	5
4	3	4	4	4	3	3	4	4
5	3	4	4	4	4	4	3	4
5	5	5	4	4	5	5	5	5
5	5	4	5	4	5	5	5	4
5	3	4	5	4	5	5	5	5
4	4	4	4	4	4	4	4	4
4	3	4	4	4	4	4	4	4
3	3	3	3	3	3	3	3	3
5	5	5	5	5	5	5	5	4
5	5	5	4	5	5	3	5	5
3	3	3	3	4	4	4	4	4
4	4	3	4	4	3	4	3	4
4	4	4	4	4	4	4	4	4
4	4	4	4	5	5	5	5	5
5	5	5	5	5	5	5	5	5
3	3	3	3	3	3	3	3	3
4	4	4	4	5	4	4	5	5
4	4	4	4	4	4	4	4	4
4	4	4	4	4	4	4	4	4
4	4	4	4	4	4	4	4	4
4	4	4	4	4	4	4	4	4
5	5	5	5	5	5	5	5	5
5	5	5	4	4	5	3	3	3
4	4	4	5	4	4	4	3	4
5	5	5	5	3	5	5	5	5
4	4	4	4	4	4	4	4	4
3	3	3	3	3	3	3	3	3
5	5	5	5	5	5	5	5	5
5	4	3	4	5	5	5	5	5
4	4	3	3	3	3	3	3	3

4	4	4	4	4	4	5	5	4
5	5	5	5	5	5	5	5	4
5	4	5	4	4	5	4	5	4
5	5	5	5	5	5	5	5	5
5	3	3	3	4	5	4	5	5
5	4	5	5	5	5	4	5	4
4	4	4	4	4	4	4	4	4
4	4	4	4	4	4	4	4	4
4	4	3	4	4	3	3	4	3
4	3	3	4	3	4	3	4	4
4	4	4	5	5	5	5	5	5
4	4	4	4	4	4	4	4	4
4	4	4	4	4	4	4	4	4
5	5	5	5	5	5	5	5	3
5	5	5	5	5	4	5	5	5
4	4	4	4	4	4	4	3	4
5	5	5	5	5	5	5	5	5
4	4	4	3	4	4	4	4	4
5	5	5	5	5	5	5	5	5
4	4	4	4	4	4	4	4	4
4	3	2	4	4	4	4	2	4
4	5	4	4	4	4	5	5	5
4	4	4	4	3	4	2	5	3
1	5	1	5	1	4	1	4	5
4	4	4	4	4	4	4	4	5
4	4	4	4	4	4	4	5	5
5	5	5	5	5	5	5	5	5
5	4	4	4	4	5	4	4	4
5	5	4	4	4	4	4	4	4
5	4	4	4	4	5	4	4	3

C10	C11	C12	C13	C14	C15	C16	C17	C18
4	4	4	4	4	5	5	5	5
4	3	2	4	3	2	3	3	4
3	4	5	4	5	3	4	5	4
3	4	5	4	5	3	4	5	4
3	4	5	4	5	3	4	5	4
4	3	4	4	5	4	4	5	4
4	3	4	4	5	4	4	5	4
4	3	4	4	3	4	3	4	4
3	4	4	4	4	5	4	5	4
4	5	5	5	5	5	4	5	5
5	5	5	5	5	5	4	4	4
5	2	4	5	5	5	2	4	5
3	3	4	4	4	4	4	4	4
5	5	5	5	5	5	4	4	4
5	5	5	5	5	5	4	4	4
3	3	4	5	4	4	5	4	4
4	4	4	4	4	4	4	4	4
3	4	4	5	5	4	5	4	4
5	4	3	4	5	4	5	4	5
4	4	4	5	4	4	4	4	5
3	5	5	5	5	5	5	5	5
4	4	5	4	5	3	4	4	5
5	5	5	5	5	5	5	5	5
5	5	4	4	4	4	4	4	4
4	4	4	4	5	5	5	5	5
4	3	4	4	5	4	4	4	5
3	5	5	5	5	5	5	5	5
5	5	4	5	5	5	5	4	5
3	5	3	5	5	5	5	5	5
3	5	4	3	4	5	4	3	2
4	5	4	5	4	4	4	4	4
5	4	3	3	4	4	5	4	5
4	5	5	5	4	4	5	5	4
4	4	3	4	5	4	3	4	5
1	5	3	5	5	5	5	1	5
5	5	4	4	5	5	4	5	5
4	4	4	4	4	4	3	4	4
3	4	4	4	4	4	4	4	4
4	5	4	4	5	5	5	5	5
3	5	4	5	5	5	4	4	5
5	4	4	4	5	5	5	4	4
2	3	4	2	3	4	2	4	3
5	5	4	5	5	5	5	5	5
4	5	4	5	4	4	4	4	4
3	3	3	3	3	3	3	3	3
4	5	5	4	4	4	5	5	4
4	4	4	5	5	4	4	4	4
5	4	4	5	4	5	4	5	4
5	5	4	4	4	4	5	5	5
3	4	4	4	3	3	4	4	3
5	5	5	5	4	4	4	5	5

4	4	4	4	4	4	4	4	4
4	4	5	5	4	5	4	4	5
4	4	4	5	5	5	4	4	4
4	4	3	5	5	4	3	4	5
3	4	4	4	3	3	3	4	4
5	4	3	3	4	5	4	4	5
5	4	4	4	5	5	5	4	4
4	5	4	5	4	5	4	5	5
4	4	5	5	4	4	4	5	5
5	5	5	5	5	5	5	3	5
4	4	4	4	4	4	4	4	4
2	3	3	2	2	3	2	3	3
3	2	2	3	4	4	2	3	4
4	3	5	4	3	4	5	4	2
5	5	4	5	5	5	5	4	5
5	5	5	5	5	5	5	5	5
5	5	5	5	5	5	5	5	5
5	5	5	5	4	5	5	5	5
5	4	5	5	5	5	5	5	5
4	4	4	3	4	4	4	4	4
5	4	5	5	5	5	5	5	5
4	4	4	5	4	4	5	4	5
4	4	4	3	4	4	4	4	4
5	5	5	5	5	5	5	3	5
4	4	3	4	4	3	3	4	3
4	4	3	4	4	4	2	4	4
5	5	4	5	5	5	4	5	5
4	4	4	4	3	4	4	4	4
3	4	4	4	4	4	4	3	4
5	5	5	5	5	5	5	5	5
3	5	3	4	5	5	5	4	5
4	5	5	5	5	5	5	5	5
5	5	4	5	5	5	5	4	5
5	3	5	5	5	4	5	5	5
4	4	4	4	4	4	4	4	4
4	5	4	5	4	5	5	5	5
5	5	4	5	5	5	4	3	4
4	4	4	4	4	4	4	4	4
4	4	4	4	4	4	4	4	4
5	5	5	5	5	5	5	5	5
4	5	4	5	5	5	4	4	4
5	5	5	5	5	5	4	5	5
5	5	5	5	5	5	4	5	5
4	4	3	4	4	5	4	4	3
4	4	4	4	4	4	5	5	5
4	4	4	4	4	5	4	4	4
3	4	2	4	4	5	3	5	5
4	4	4	4	4	4	4	4	4
4	3	4	4	3	4	4	3	4
4	4	4	4	4	4	4	4	5
4	3	4	4	4	4	3	4	4
4	4	5	5	5	4	4	5	4
4	4	5	5	5	4	4	5	4
5	4	4	5	4	5	5	4	5

4	4	3	4	4	4	4	4	4
3	3	3	3	4	4	4	4	4
5	5	5	5	5	5	5	5	5
4	3	3	3	4	3	3	3	3
4	4	3	4	4	5	5	4	5
5	4	4	4	5	4	4	5	3
4	4	4	4	4	4	4	4	4
4	4	3	4	4	4	4	4	4
4	4	3	4	4	4	4	4	4
5	5	5	5	5	5	5	4	5
5	5	5	5	4	4	4	4	4
4	4	3	4	4	4	4	4	4
4	4	4	3	3	3	4	4	4
4	4	4	4	4	4	4	4	4
3	4	4	4	4	4	4	4	4
3	3	3	3	3	3	3	3	3
5	5	5	5	5	5	5	5	5
5	5	4	5	5	4	5	5	5
4	5	3	4	5	5	5	5	5
5	5	4	4	5	5	5	4	4
5	4	4	5	5	4	5	5	5
5	5	5	5	5	5	4	4	4
3	2	1	2	3	4	5	4	3
3	4	4	4	4	4	4	4	4
4	4	5	5	5	5	5	5	5
4	4	4	4	5	5	5	5	5
4	4	3	4	5	4	4	4	5
2	2	2	1	2	5	3	2	5
5	5	5	5	5	5	5	5	4
5	5	5	4	5	5	4	4	4
5	5	4	5	5	5	5	5	5
4	4	4	4	5	5	5	5	5
4	5	5	5	5	4	5	5	5
5	4	5	5	4	5	4	3	4
4	5	4	5	4	4	5	5	4
3	3	4	4	4	4	4	2	3
3	3	3	3	3	3	4	4	4
3	3	3	3	3	4	4	3	4
5	4	5	5	5	5	5	5	5
4	3	4	4	4	4	4	3	4
3	2	5	5	4	4	5	5	4
4	5	4	5	5	5	5	5	5
4	5	4	5	5	5	5	5	5
3	4	4	4	4	4	4	4	4
5	5	5	5	5	5	5	5	5
5	5	3	5	5	5	4	5	5
4	4	4	3	4	3	4	4	4
4	4	4	4	4	5	4	3	4
4	4	4	4	4	4	4	4	4
4	4	4	4	4	4	4	4	4
4	4	4	4	4	4	4	4	4
5	5	2	3	5	5	4	3	4
3	3	4	4	4	4	4	4	5
4	4	4	4	4	4	4	4	4

5	2	4	4	4	5	4	4	2
4	4	4	4	4	4	4	4	4
4	4	4	4	4	4	4	4	4
4	4	4	4	4	5	4	5	4
3	5	4	4	5	4	4	5	5
5	5	4	5	5	5	5	4	5
4	4	4	4	4	4	4	4	4
4	4	5	5	4	5	4	4	4
4	5	4	4	5	5	4	4	5
1	5	5	5	4	5	5	5	4
4	4	4	4	4	4	4	4	4
4	4	4	4	4	4	4	4	4
3	4	2	4	4	4	4	4	4
5	5	5	5	5	5	5	5	5
3	4	3	4	4	5	2	4	4
4	4	3	2	4	4	4	4	4
5	5	5	5	5	5	5	5	5
5	5	3	4	4	4	4	4	4
4	5	4	4	4	5	4	5	5
5	4	4	5	4	4	4	4	4
5	5	5	5	5	5	5	4	5
4	4	4	4	4	4	4	4	5
5	5	5	5	5	5	5	5	5
4	4	4	4	4	4	4	4	4
5	5	5	5	4	4	4	5	4
3	3	3	4	4	4	4	4	4
5	5	4	4	4	4	4	5	4
5	5	5	5	5	5	5	5	5
4	4	4	4	4	4	4	4	4
5	5	5	5	5	5	4	5	5
5	4	4	4	5	5	5	5	5
4	4	5	5	5	5	5	5	5
5	5	5	5	5	5	4	5	5
5	5	4	5	5	5	5	4	5
4	4	4	4	4	4	4	4	4
3	5	3	4	5	5	4	4	5
5	5	5	5	5	5	5	5	5
5	4	4	4	4	4	3	3	3
4	4	4	4	4	4	4	4	4
3	4	5	4	4	5	4	4	5
4	5	5	5	5	5	4	4	5
4	4	5	5	5	4	4	4	5
4	4	5	4	4	4	4	4	4
4	4	4	4	5	5	4	4	5
4	4	3	4	5	5	5	5	5
5	5	5	5	5	5	5	5	5
4	4	4	4	4	5	5	4	5
4	4	4	5	4	4	4	4	4
3	4	2	5	5	5	5	5	5
4	4	4	4	4	4	4	5	5
5	4	5	4	5	4	5	4	5
5	5	5	5	5	5	5	5	5
5	5	5	5	5	5	5	5	5
4	4	5	5	5	5	3	5	5

4	4	4	4	4	4	4	4	4
4	5	4	5	4	5	4	5	5
4	4	4	5	5	5	3	4	5
5	5	5	5	5	5	5	5	5
3	4	2	3	4	4	3	4	4
4	4	4	4	4	4	4	4	4
5	5	3	5	5	5	5	4	5
5	5	4	5	5	5	5	5	5
4	4	5	4	4	4	4	4	4
3	4	4	4	4	4	5	5	5
5	5	5	5	5	5	5	4	5
4	5	4	4	4	4	4	4	4
4	3	4	4	3	3	3	4	3
5	5	4	5	5	5	4	5	5
5	5	5	5	5	5	5	5	5
5	4	5	4	5	4	4	3	5
4	5	4	5	5	5	4	5	5
4	4	4	4	4	4	4	4	4
4	4	4	5	5	4	4	3	5
4	4	4	4	4	4	4	4	4
4	4	4	4	4	4	4	4	4
4	4	4	4	4	4	4	4	4
3	4	5	4	3	4	4	4	5
4	4	4	4	5	5	5	5	5
3	3	4	4	4	4	4	4	4
3	4	4	5	4	4	5	4	3
4	4	4	4	5	4	4	4	4
4	4	4	4	4	4	4	4	4
5	5	5	5	4	5	4	5	4
4	4	5	5	5	5	5	5	5
5	4	5	4	4	5	5	4	4
3	5	4	4	4	4	4	4	4
4	4	4	5	3	4	4	4	4
3	4	4	4	4	4	4	5	4
4	3	4	4	3	3	4	3	4
5	5	1	5	5	5	5	5	5
5	5	4	4	4	4	5	4	4
4	4	4	4	4	4	4	4	4
5	5	5	5	5	5	5	5	5
4	4	4	4	4	4	4	4	4
1	2	3	4	5	4	3	2	1
4	4	4	4	4	4	4	4	4
4	4	4	4	4	4	4	4	4
3	4	4	5	4	4	4	4	5
5	5	5	5	5	5	5	5	5
5	5	4	5	5	5	5	5	5
4	4	4	4	4	4	4	4	4
5	5	4	5	5	5	5	5	5
5	5	5	5	5	5	5	5	5
5	5	5	5	5	5	5	5	5
2	4	3	2	5	5	5	3	5
4	5	4	5	4	5	4	4	4
5	5	5	5	5	5	5	5	5
5	5	4	4	5	5	5	5	5

4	4	4	4	4	4	4	4	4
5	5	5	5	5	5	5	5	5
4	4	5	5	5	4	4	5	4
5	3	4	5	4	5	4	5	4
4	4	4	4	4	4	4	4	4
4	4	4	4	4	4	4	4	4
4	4	4	4	4	4	4	4	4
5	4	4	5	5	5	5	5	5
5	5	3	5	5	5	4	4	5
4	4	5	4	4	4	4	5	4
5	5	5	5	5	5	5	5	5
5	5	5	5	5	5	5	5	5
4	5	4	5	4	5	4	5	4
4	4	5	5	4	4	4	4	5
4	4	4	4	4	4	4	4	4
4	4	4	3	3	4	3	4	4
4	4	4	4	4	4	4	4	4
5	5	4	4	5	5	4	4	4
4	4	4	4	4	4	4	4	4
4	4	4	3	3	4	3	4	4
4	4	4	4	4	4	4	4	4
3	4	3	4	3	4	3	4	4
4	4	4	4	4	4	4	4	4
5	5	5	5	5	5	4	5	4
4	4	4	5	4	5	4	5	4
3	5	1	2	4	4	3	1	5
5	5	4	4	5	5	5	5	4
5	4	5	4	5	4	5	5	5
4	3	3	4	5	5	4	5	5
4	4	4	4	4	4	4	4	4
4	4	3	4	4	4	4	3	4
3	3	3	3	3	3	3	3	3
5	5	5	4	5	5	4	4	4
5	5	4	4	4	4	4	5	5
4	4	4	3	3	4	4	4	4
3	4	3	4	5	4	4	3	3
4	4	4	4	4	4	4	4	4
4	4	5	5	4	5	5	5	5
5	5	5	5	5	5	5	5	5
3	3	3	3	3	3	3	3	4
4	5	4	3	5	5	4	4	4
4	4	4	4	4	4	4	4	4
4	4	4	4	4	4	4	4	4
4	4	4	4	4	3	4	4	4
4	4	4	4	4	4	4	4	4
5	5	5	5	5	4	4	4	5
5	5	5	5	5	5	5	5	5
4	4	4	4	4	4	4	4	4
5	5	5	3	3	5	5	5	5
4	4	4	4	4	4	4	4	4
3	3	3	3	3	3	3	3	3
5	5	5	5	5	5	4	5	5
5	5	5	5	5	5	4	4	4
3	3	3	3	3	3	3	3	3

5	5	5	4	4	5	5	5	5
5	5	5	5	5	5	5	5	5
5	4	5	4	4	5	4	5	4
5	5	5	5	5	5	4	5	5
5	5	5	3	3	5	5	5	5
4	5	5	5	5	5	4	5	5
4	4	4	4	4	4	4	4	4
4	4	4	4	4	4	4	4	4
4	3	4	3	4	4	4	3	3
4	3	3	4	4	4	4	3	3
5	5	5	5	5	5	4	4	5
4	4	4	4	4	4	5	4	4
4	4	4	4	4	4	4	4	4
5	5	5	5	3	5	5	5	5
5	5	5	5	5	5	4	3	4
3	4	3	4	4	4	4	3	2
5	5	5	5	5	5	5	5	5
4	4	4	4	4	4	4	4	3
5	5	5	5	4	4	4	4	4
4	4	4	4	4	4	4	4	4
4	4	5	5	5	5	4	3	3
5	4	4	4	5	4	5	4	5
4	5	4	4	4	5	4	4	4
3	2	4	1	3	3	1	5	1
4	4	4	4	4	4	3	4	4
5	5	5	4	4	4	4	4	4
5	5	5	5	5	5	5	5	5
4	4	4	5	5	4	4	4	4
4	4	4	4	4	4	4	4	4
4	4	5	5	4	5	4	4	5

professional identity(C)	D1	D2	D3	D4	D5	D6	D7	D8
4.72	5	5	5	5	5	5	5	5
2.89	3	2	4	3	2	4	2	4
4.17	5	4	3	5	4	5	4	4
4.22	5	4	3	5	4	5	4	4
4.22	5	4	4	5	4	5	4	4
4.17	4	4	4	5	5	4	4	4
4.22	4	4	4	4	5	4	5	4
3.67	3	3	2	3	3	4	4	4
3.89	4	3	2	4	4	4	4	5
4.39	3	3	3	3	3	4	4	4
4.61	5	4	5	4	5	5	5	5
4.28	5	4	2	5	5	5	5	5
3.67	4	4	3	4	4	4	4	4
4.50	4	4	4	2	3	4	4	4
4.50	4	4	4	2	3	4	4	4
4.06	4	3	3	4	5	5	5	5
4.11	4	5	4	4	4	4	4	4
4.06	3	4	5	5	5	4	3	4
4.28	4	3	4	5	4	3	4	5
4.22	4	4	3	4	4	4	4	4
4.56	4	4	4	4	4	4	4	4
4.00	5	4	4	4	5	5	5	4
4.94	5	5	3	4	4	4	4	5
4.50	4	4	4	4	5	4	4	5
4.56	4	4	4	4	4	4	5	5
3.89	4	4	3	4	4	4	4	4
4.39	5	4	3	5	4	4	4	4
4.67	5	5	4	4	4	4	5	5
4.50	3	3	3	4	3	5	5	5
3.61	2	3	4	5	4	5	4	3
4.39	4	4	4	4	5	4	5	4
4.17	3	4	5	4	3	4	4	4
4.50	3	4	5	4	4	4	5	4
3.89	2	3	4	5	4	3	4	5
3.83	4	4	3	4	4	5	5	5
4.50	5	5	5	4	4	5	5	5
3.94	4	4	4	3	3	4	3	4
3.89	4	4	3	4	4	4	4	4
4.11	4	4	4	5	5	4	3	3
4.67	5	5	4	5	5	5	5	5
4.61	5	4	4	4	4	5	4	5
3.00	3	4	3	4	3	3	3	4
4.83	5	5	4	5	5	5	5	5
4.39	4	5	5	4	4	5	5	4
3.17	3	4	4	4	3	4	4	3
4.56	4	5	5	5	5	4	4	4
4.44	4	4	4	5	5	4	4	4
4.44	3	4	4	5	4	3	4	4
4.44	4	4	4	4	4	5	5	4
3.94	4	4	4	5	5	4	3	4
4.56	4	4	4	4	4	4	4	3

4.00	4	4	4	4	4	4	4	5
4.44	4	4	5	5	5	4	4	4
4.50	4	4	5	5	5	5	5	5
4.11	3	4	5	5	4	4	4	4
3.44	3	3	3	3	3	3	3	3
4.17	3	4	5	4	3	3	4	5
4.44	5	5	5	4	4	4	5	4
4.44	4	4	4	4	5	5	5	4
4.28	4	3	2	3	3	4	4	4
4.61	5	5	4	5	5	5	5	5
4.00	4	4	2	4	4	4	4	4
2.61	3	3	3	2	2	3	2	3
2.94	3	2	3	4	4	4	4	4
3.61	4	4	2	4	3	4	3	2
4.72	5	5	3	5	3	5	5	5
5.00	4	4	4	4	4	4	4	4
4.89	5	5	5	5	5	5	5	5
4.83	4	4	3	4	4	4	4	4
4.28	4	4	4	4	4	4	4	4
3.89	3	3	2	3	3	3	3	3
4.78	4	4	3	4	4	5	5	5
4.61	4	4	4	5	5	5	5	5
3.89	3	3	2	3	3	3	3	3
4.56	4	4	3	4	4	4	4	4
3.50	4	5	2	3	3	3	3	3
3.72	4	4	4	4	4	4	4	4
4.89	4	4	4	4	4	4	4	5
3.83	4	4	3	3	3	3	4	4
3.67	4	4	2	4	3	4	4	3
4.83	5	5	4	4	4	5	5	5
4.22	4	3	4	4	4	4	4	4
4.56	4	4	3	4	4	3	3	4
4.72	4	5	4	4	4	5	5	5
4.50	5	5	5	5	5	4	4	4
4.00	4	4	3	3	3	4	4	4
4.50	4	4	3	4	4	4	5	5
4.39	3	3	3	3	3	3	3	3
4.06	4	4	4	4	4	4	4	4
4.00	3	3	3	3	3	3	3	3
5.00	4	4	3	4	4	4	4	4
4.28	4	4	4	4	5	5	5	4
4.89	4	4	2	4	4	4	4	4
4.89	4	4	1	4	4	5	5	5
4.00	4	5	4	5	4	4	3	4
4.44	4	4	4	5	4	5	4	5
4.06	4	4	3	4	4	4	4	5
4.00	4	4	4	4	4	5	5	5
4.06	4	4	3	4	4	4	4	4
3.78	4	4	3	4	4	4	4	4
4.17	4	4	4	4	4	4	4	4
4.06	4	4	4	5	5	5	5	4
4.50	5	4	4	5	4	4	4	5
4.50	5	4	4	5	4	4	4	5
4.39	4	3	2	5	4	4	4	4

3.89	4	4	3	4	4	4	4	4
3.61	3	3	2	2	3	4	4	4
5.00	5	5	5	5	5	5	4	5
3.28	4	4	4	4	3	3	3	3
4.22	4	4	3	4	4	4	5	5
4.00	3	3	3	4	3	4	3	4
3.89	4	4	3	3	4	4	4	4
3.94	4	3	3	3	3	4	4	4
3.94	4	3	3	3	3	4	4	4
4.89	5	5	4	4	4	5	5	5
4.61	5	5	2	2	3	3	4	4
3.94	4	3	3	3	3	4	4	4
3.50	3	3	3	4	4	4	3	3
3.83	4	4	2	4	4	4	4	4
3.72	4	4	2	4	4	4	4	4
2.89	3	3	2	3	2	2	2	2
5.00	5	5	5	5	4	5	5	4
4.56	4	4	4	4	4	4	4	5
4.39	4	4	4	4	4	5	5	4
4.44	3	4	3	5	3	5	3	5
4.56	5	5	5	4	5	5	4	5
4.50	5	5	5	5	5	5	5	5
3.22	5	5	5	5	5	4	4	4
3.94	3	4	2	4	4	4	4	3
4.67	4	4	4	4	4	4	5	5
4.56	4	4	3	4	4	4	4	4
4.11	4	4	4	4	5	5	5	5
3.00	4	4	5	4	4	5	4	4
4.56	5	5	4	4	4	4	3	4
4.22	3	3	3	4	4	4	4	4
4.83	4	4	4	4	4	5	5	5
4.61	5	5	5	5	5	5	5	5
4.61	4	5	4	4	5	5	5	4
4.50	4	5	4	4	4	5	4	4
4.44	4	4	5	4	5	4	5	4
3.44	4	3	2	2	2	2	3	3
3.67	4	4	4	4	4	3	3	3
3.44	4	4	2	2	2	4	4	4
4.61	4	4	3	5	4	5	5	5
3.89	4	4	3	4	4	4	4	4
4.22	3	3	3	4	3	4	3	4
4.61	4	4	3	5	4	5	5	5
4.61	4	4	3	5	4	5	5	5
3.67	4	4	2	4	4	4	4	4
5.00	5	5	4	4	4	4	5	5
4.50	4	4	3	4	4	4	4	4
3.83	4	4	4	3	3	3	3	4
4.22	4	4	4	4	4	4	4	5
4.33	4	4	3	4	4	4	4	4
4.00	5	5	5	5	5	5	5	5
4.00	4	4	3	3	4	4	4	4
4.11	4	4	3	4	4	4	4	5
3.89	4	4	3	5	5	5	5	5
4.00	3	3	3	3	3	3	3	4

3.67	4	2	2	3	3	2	2	5
3.83	4	4	4	4	4	4	4	4
3.94	3	4	3	3	3	4	4	4
4.11	3	4	5	3	4	4	5	4
4.17	3	3	3	4	4	4	4	4
4.72	4	4	3	4	4	4	4	4
3.83	4	4	3	4	4	4	4	4
4.22	4	4	4	5	5	5	5	4
4.33	5	5	4	5	5	4	4	4
3.78	4	4	2	5	3	4	5	5
4.00	4	4	3	5	5	4	4	4
4.00	4	4	3	4	4	4	4	4
3.78	4	4	2	4	4	4	4	4
5.00	4	4	3	4	4	4	5	5
3.83	4	4	3	4	4	5	5	5
3.83	2	4	3	5	3	4	4	4
5.00	4	4	4	4	4	4	4	4
4.44	4	4	4	5	5	5	4	5
4.28	1	3	2	5	4	5	5	4
4.39	5	5	5	5	5	5	5	5
4.72	4	4	4	5	4	5	4	4
4.11	4	4	4	5	4	5	4	4
5.00	4	4	4	4	4	4	4	4
4.00	5	4	4	4	4	4	4	4
4.78	4	4	3	4	5	4	4	5
3.61	4	4	3	4	4	4	4	4
4.33	4	4	3	3	4	4	4	4
4.78	3	4	3	5	3	4	4	5
4.22	4	4	2	4	4	4	4	4
4.61	4	4	2	4	2	4	4	4
4.50	4	4	3	4	4	3	4	4
4.61	4	4	3	4	5	4	4	4
4.94	4	4	2	3	3	4	4	4
4.56	4	4	4	4	4	4	4	4
4.22	4	4	2	4	4	4	4	4
4.00	3	4	5	4	4	4	4	4
5.00	5	5	5	5	5	5	5	5
4.00	5	5	4	5	5	5	5	5
4.00	4	4	2	3	3	3	3	4
4.06	3	2	3	4	3	4	3	2
4.61	4	4	3	4	4	4	4	4
4.22	4	4	5	5	5	4	4	5
4.00	4	3	3	4	4	4	4	4
4.28	4	4	4	4	4	4	4	4
4.28	3	4	3	5	5	5	4	4
5.00	5	5	4	5	5	5	5	5
4.17	2	2	2	4	2	3	2	4
4.00	4	3	2	3	4	3	4	4
4.33	4	4	3	3	3	4	4	3
4.17	3	4	4	4	4	4	4	4
4.44	4	4	4	4	4	4	4	4
4.94	5	5	4	4	4	5	5	5
4.94	5	5	4	4	4	5	5	5
4.44	4	4	3	3	3	4	4	4

4.00	4	4	4	4	4	4	4	4
4.33	3	3	4	5	5	5	5	4
4.44	4	4	3	4	5	4	5	5
4.67	5	5	5	5	5	5	5	5
3.28	4	4	3	3	4	3	3	4
4.00	3	3	3	4	4	4	4	4
4.83	5	5	4	5	5	5	5	5
4.89	4	4	3	3	4	4	3	5
4.06	4	4	4	4	4	5	5	4
4.28	5	5	2	4	2	4	4	4
4.83	4	4	4	4	5	5	5	5
4.11	4	4	4	4	4	4	4	4
3.61	4	3	4	3	3	4	4	4
4.72	4	5	3	5	5	5	5	5
5.00	5	5	2	5	5	5	5	5
4.39	4	4	3	3	3	4	4	3
4.50	3	4	3	4	2	3	4	4
4.00	4	4	4	4	4	4	4	4
4.06	4	4	3	4	4	4	4	5
3.83	3	2	2	3	4	3	3	3
4.00	4	4	4	4	4	4	4	4
3.89	4	4	2	3	4	3	4	3
4.00	4	4	3	3	4	3	3	4
4.33	4	4	2	4	4	4	4	4
3.61	4	4	3	4	4	4	4	4
4.11	4	3	4	5	5	4	3	4
4.17	4	4	4	4	4	4	4	4
4.00	4	4	4	4	4	4	4	4
4.83	4	4	4	4	4	4	4	4
4.61	4	4	5	5	5	5	4	4
4.39	4	5	5	5	5	5	5	5
3.83	4	4	4	4	4	4	4	4
4.06	4	4	4	5	4	4	5	5
3.83	4	4	2	4	4	4	4	4
3.50	3	4	4	3	5	3	5	3
4.67	4	4	1	5	5	4	3	4
4.22	3	3	3	4	4	4	3	5
4.06	4	4	3	4	3	4	4	4
5.00	5	3	2	3	4	5	5	5
4.00	4	4	4	3	3	3	3	3
2.94	1	2	3	4	5	4	3	2
4.00	4	4	4	2	4	4	4	4
3.94	4	4	3	4	4	4	3	3
4.06	4	3	3	4	4	4	4	4
4.89	4	4	4	4	4	4	4	4
4.67	4	4	3	3	4	4	5	5
3.89	4	4	3	3	4	5	5	4
4.83	4	5	2	4	4	4	4	4
5.00	4	4	4	4	4	5	5	5
5.00	4	5	3	5	4	4	4	4
3.89	3	4	3	2	3	4	4	5
4.28	4	5	4	4	4	4	5	5
4.89	5	5	5	5	5	5	5	5
4.44	4	4	4	4	4	4	4	5

4.17	4	4	4	4	4	4	4	4
4.94	5	5	5	5	5	5	5	5
4.61	4	3	4	4	3	4	4	3
4.33	4	4	4	4	4	3	3	5
4.17	4	4	4	4	4	4	4	4
4.00	4	4	4	4	4	4	4	4
3.83	3	4	4	3	4	4	4	4
4.61	4	4	4	4	4	4	4	4
4.72	4	4	4	4	4	4	4	4
4.11	4	5	3	4	4	4	4	4
4.89	4	4	4	4	4	4	4	4
5.00	5	5	4	5	5	5	5	5
4.39	4	4	4	3	3	4	4	3
4.33	4	4	4	4	4	4	5	5
4.00	4	5	5	5	5	5	4	4
3.89	4	4	4	4	3	3	3	3
4.00	4	4	4	4	4	4	4	4
4.56	3	3	4	3	4	4	4	3
4.00	4	5	5	5	5	5	4	4
3.89	4	4	4	4	3	3	3	3
4.00	4	4	4	4	4	4	4	4
3.44	3	2	4	2	3	2	4	2
4.00	3	4	4	4	3	3	3	4
4.56	4	4	4	5	4	5	4	5
4.00	3	3	3	4	3	4	3	4
3.50	4	4	4	4	4	4	4	4
4.72	4	4	4	4	5	5	5	5
4.67	4	5	5	5	4	4	5	4
4.39	4	4	3	4	4	4	4	5
4.00	4	4	2	3	3	4	4	4
3.83	4	4	3	4	4	4	4	4
3.00	3	3	3	3	3	3	3	3
4.72	4	4	3	4	4	4	4	5
4.56	5	4	5	5	5	5	5	5
3.67	4	4	4	4	3	4	4	4
3.67	3	4	4	3	4	4	4	3
4.00	4	4	4	4	4	4	4	4
4.61	5	5	5	5	5	5	5	5
5.00	3	3	3	5	5	5	5	5
3.06	3	3	4	3	4	3	3	4
4.28	3	3	3	3	3	3	3	3
4.00	4	4	4	4	4	4	4	4
4.00	4	4	4	4	4	4	3	4
3.94	4	4	4	4	4	3	4	4
4.00	4	4	4	4	4	4	4	4
4.83	4	4	4	4	4	4	4	4
4.56	4	4	2	4	4	4	4	4
4.00	4	4	4	5	4	4	5	4
4.67	3	3	3	3	3	5	5	5
4.00	4	4	4	4	4	4	4	4
3.00	3	3	3	3	3	3	3	3
4.94	3	5	3	5	5	5	3	4
4.61	5	5	5	5	5	5	5	3
3.11	3	3	3	3	3	3	3	4

4.50	5	5	5	5	5	5	5	5
4.94	4	4	4	3	5	5	5	5
4.44	4	4	4	5	5	5	5	4
4.94	5	5	5	5	5	5	5	5
4.33	4	4	4	4	4	4	5	5
4.72	5	5	5	5	5	5	5	4
4.00	4	4	2	2	4	4	3	4
4.00	4	4	4	4	4	4	4	4
3.56	2	4	4	3	3	4	3	4
3.56	3	3	4	4	4	4	3	3
4.72	4	2	3	2	4	4	3	4
4.06	4	4	4	4	4	4	4	4
4.00	4	4	4	4	4	4	4	4
4.78	5	5	5	4	4	5	5	5
4.72	4	4	4	2	2	2	2	3
3.67	4	3	4	4	4	4	3	4
5.00	5	5	5	5	5	5	5	5
3.89	4	4	4	3	5	4	3	5
4.72	4	4	4	4	3	4	2	4
4.00	4	4	4	4	4	4	4	3
3.83	4	5	3	5	5	3	4	3
4.44	4	4	5	5	5	4	5	5
3.94	4	4	2	3	5	5	4	4
2.78	5	1	5	1	2	1	5	3
4.00	4	4	3	4	4	4	4	4
4.28	5	5	5	5	5	5	5	5
5.00	4	5	2	4	4	2	3	4
4.22	4	4	4	5	4	4	4	4
4.11	5	5	5	5	5	5	5	4
4.28	3	5	3	4	4	4	4	4

D9	D10	D11	D12	D13	D14	D15	D16	D17
5	5	5	5	5	5	5	5	5
3	2	4	2	3	3	3	2	5
5	5	5	5	4	4	4	4	4
5	5	5	5	4	4	4	4	4
5	5	5	5	4	4	4	4	4
4	5	4	5	5	4	5	5	4
5	4	5	4	5	4	4	5	5
4	4	3	3	4	4	4	3	4
4	4	4	3	4	4	3	3	3
4	4	4	4	4	4	4	2	3
5	5	4	4	4	4	4	5	5
2	4	5	5	5	5	5	5	5
4	4	4	4	4	4	4	4	4
4	4	4	4	4	4	4	4	5
4	4	4	4	4	4	4	4	5
5	5	4	4	3	4	4	4	5
4	4	4	4	4	4	4	3	4
5	5	5	4	4	4	5	5	4
4	3	4	5	4	3	4	5	4
4	4	4	4	5	4	4	4	4
5	5	5	5	5	5	5	5	5
5	5	5	5	4	4	3	5	5
5	5	5	5	5	5	5	3	5
4	5	4	5	5	5	5	5	5
5	4	4	4	4	5	5	5	5
4	4	4	4	4	4	4	4	4
4	4	4	4	4	4	4	4	4
5	4	5	5	5	5	5	4	5
5	5	5	5	5	5	5	4	5
2	3	4	5	4	3	3	4	4
3	4	4	5	5	5	4	5	5
5	4	4	4	4	4	4	5	5
4	5	5	5	5	5	5	4	4
4	4	3	4	5	4	3	5	4
5	5	5	5	5	5	5	5	5
5	4	4	5	4	5	4	5	4
4	4	3	4	3	3	4	3	3
4	3	4	4	4	4	4	4	4
3	3	3	3	4	4	4	5	4
5	4	5	5	5	5	5	5	5
4	4	5	4	5	4	4	5	4
4	4	3	3	3	4	4	2	3
5	5	5	5	5	5	5	5	5
4	5	4	4	5	4	4	4	4
3	4	4	4	4	4	4	4	4
4	5	4	5	4	5	5	4	4
4	4	5	5	5	4	4	4	4
4	5	5	5	4	4	4	4	5
4	4	5	5	4	4	4	4	5
4	4	4	4	3	3	5	5	5
4	4	4	4	4	4	4	4	3

3	4	4	4	4	3	3	5	5
4	4	5	4	4	4	5	4	4
5	4	5	5	4	4	4	4	5
4	4	3	4	5	4	3	4	5
3	3	4	4	4	4	4	4	4
4	4	4	5	4	4	4	3	4
4	4	5	5	4	4	4	5	5
5	5	5	4	5	5	5	4	4
5	4	4	3	4	4	4	5	5
2	5	3	3	3	4	4	4	4
4	4	4	4	4	4	4	4	4
2	3	3	3	2	3	2	2	2
3	2	3	3	3	3	3	3	4
4	3	2	4	4	3	2	4	3
5	5	5	5	5	5	5	5	5
4	4	4	4	4	4	4	4	4
5	5	5	5	5	5	5	5	5
4	4	4	4	4	4	4	4	4
4	4	4	4	4	4	4	4	4
3	2	2	2	2	3	3	3	3
4	4	5	5	5	4	4	4	5
4	5	3	3	4	4	4	4	4
3	2	2	2	2	3	3	3	3
3	5	3	5	5	5	5	3	5
3	3	3	3	3	3	3	3	3
4	4	4	4	4	4	4	4	4
5	4	5	5	5	5	5	5	5
4	4	4	4	4	4	4	4	4
3	3	3	3	2	2	4	3	4
4	4	4	3	4	4	4	4	5
5	3	3	3	4	4	4	4	3
4	4	4	4	4	4	4	4	4
5	5	5	5	5	5	5	5	5
5	4	3	4	4	4	5	3	5
4	4	3	3	4	4	4	3	4
4	4	4	4	3	5	5	4	4
3	3	3	3	3	3	3	3	3
5	5	5	4	4	4	4	4	4
3	3	3	3	3	3	3	3	4
4	4	4	4	4	4	4	4	4
5	5	4	4	5	4	5	4	5
3	4	4	4	3	3	4	3	4
2	2	4	5	4	4	4	4	4
4	4	5	4	4	3	4	4	4
4	5	4	4	5	5	5	4	4
4	4	4	4	4	4	4	4	4
4	3	3	3	4	4	4	4	4
4	4	4	4	4	4	4	4	4
4	4	4	4	4	4	4	4	4
3	4	4	4	4	4	4	4	4
4	4	5	4	4	4	5	5	5
4	5	4	4	4	5	4	4	4
4	5	4	4	4	5	4	4	4
3	4	4	4	4	4	4	4	4

4	4	4	4	4	4	4	4	4
2	3	3	3	3	3	4	3	4
5	5	5	4	5	5	5	5	4
3	3	3	3	3	3	4	3	3
4	4	4	4	4	4	4	4	4
4	4	3	4	3	3	3	3	4
3	4	5	4	4	5	3	4	4
4	3	3	3	3	3	3	2	4
4	3	3	3	4	4	4	3	4
5	5	5	5	5	5	5	5	5
4	4	4	4	3	3	4	4	4
4	3	3	3	4	4	4	3	4
3	4	4	4	3	3	3	4	4
4	4	4	4	4	3	4	4	4
4	4	4	4	4	4	4	4	4
3	2	2	2	2	2	2	2	2
4	5	4	5	5	5	5	5	5
4	5	5	4	5	4	5	4	4
4	5	5	4	5	5	4	5	5
4	4	4	5	4	4	3	4	4
5	4	5	5	5	4	5	5	5
5	5	5	5	5	5	5	5	5
4	4	3	4	5	5	5	5	5
4	4	3	4	3	2	3	3	4
5	5	5	5	5	5	5	5	5
4	4	4	4	4	4	4	4	4
4	4	5	5	5	5	5	5	5
3	4	5	4	4	4	4	3	3
4	4	4	4	4	4	5	5	5
4	4	4	4	4	4	4	4	4
4	5	5	5	5	5	5	5	5
5	5	5	4	4	5	5	5	5
5	5	5	5	4	4	5	4	4
4	5	3	4	5	4	4	3	4
5	4	5	4	5	4	5	4	5
4	2	2	2	2	2	3	2	5
3	4	4	4	4	4	4	4	4
4	4	4	4	4	4	4	4	4
4	3	4	4	4	4	4	5	4
4	4	4	4	4	4	4	4	4
4	5	5	5	4	4	4	3	4
4	3	4	4	4	4	4	5	4
4	3	4	4	4	4	4	5	4
4	4	4	4	4	3	4	4	4
5	5	4	5	5	5	5	5	5
4	4	4	4	4	4	4	4	4
4	4	3	4	3	4	3	4	4
3	4	4	4	4	4	4	4	4
3	4	4	4	4	4	3	4	4
5	4	4	4	4	4	4	4	4
4	4	4	4	4	4	4	4	3
4	4	4	3	3	3	4	4	4
5	4	4	4	5	3	3	3	4
4	4	4	4	3	3	3	3	4

2	2	2	2	3	4	4	4	4
4	4	4	4	4	4	4	4	4
4	3	4	4	4	4	4	4	4
4	5	4	4	4	4	5	4	4
2	3	3	4	3	4	4	3	4
3	4	4	4	4	4	4	4	4
4	4	4	2	4	4	4	4	4
4	5	5	4	4	5	5	4	4
5	5	5	5	4	4	4	4	4
5	4	5	5	3	3	5	3	4
4	4	4	4	4	4	4	4	5
4	4	4	4	4	4	4	4	4
4	4	4	4	4	4	4	4	5
5	4	4	4	5	5	5	5	5
5	4	4	4	5	5	4	5	5
2	2	2	2	2	2	2	4	5
4	4	4	4	4	4	4	4	4
4	5	5	5	4	4	4	4	5
3	3	3	4	4	4	3	2	5
5	5	5	5	5	5	5	5	5
4	4	4	4	4	4	4	4	5
4	4	4	4	4	4	4	4	5
3	3	4	4	4	4	4	4	4
3	3	3	3	3	3	3	3	3
5	5	5	5	5	5	5	4	5
4	4	4	4	3	3	3	4	4
3	4	4	4	4	4	4	4	4
5	5	5	5	5	5	5	5	5
3	4	4	4	4	4	4	4	4
2	2	4	3	4	2	4	2	4
4	3	4	3	3	3	4	3	4
4	4	4	4	4	4	5	5	5
5	4	4	4	4	4	4	4	4
4	4	4	5	5	5	5	5	5
3	4	4	4	4	4	4	4	4
4	4	4	4	4	4	4	4	4
5	5	5	5	5	5	5	5	5
5	5	5	5	5	3	3	3	4
4	4	4	4	3	3	4	3	4
3	4	3	2	3	4	3	2	3
5	4	3	3	4	4	4	3	4
3	3	4	5	3	3	5	5	5
3	3	3	4	4	4	4	4	4
4	4	4	4	4	4	4	4	4
3	3	3	3	3	3	3	4	5
5	5	5	5	5	5	5	5	5
4	4	4	4	2	3	4	3	4
2	4	4	3	4	4	4	4	4
3	4	4	4	3	3	4	3	4
4	4	4	4	4	4	3	4	4
4	4	4	4	4	4	4	4	4
5	5	5	5	5	5	5	5	5
5	5	5	5	5	5	5	5	5
4	3	3	4	3	3	3	3	4

4	4	4	4	4	4	4	4	4
4	4	4	4	4	5	5	4	5
5	4	4	4	4	4	4	4	4
5	5	5	5	5	5	5	5	5
3	3	3	4	4	4	4	4	4
4	4	4	4	4	4	4	4	4
5	5	5	5	4	5	5	5	5
3	5	4	3	4	2	2	5	3
4	4	4	4	4	4	4	4	4
2	3	4	5	5	5	4	5	5
5	5	5	5	5	5	5	5	4
4	4	4	4	4	4	4	4	4
3	2	3	3	3	4	4	4	3
5	5	5	5	4	5	5	4	5
5	5	5	5	5	5	5	5	5
4	4	4	3	4	4	4	4	4
4	3	3	2	3	3	3	3	4
4	4	4	4	4	4	4	4	4
4	4	5	5	4	4	5	4	4
4	3	3	3	3	3	4	3	4
4	4	4	4	4	4	4	4	4
4	3	4	3	3	4	4	4	3
3	4	3	4	3	4	4	4	3
3	4	4	4	3	3	3	3	4
3	3	3	3	3	3	3	3	4
3	4	4	5	5	5	4	3	3
5	4	4	5	4	4	4	4	4
4	4	4	4	4	4	4	4	4
4	4	4	4	4	4	4	4	4
4	4	5	5	5	5	5	5	5
5	5	5	4	5	5	5	4	5
4	4	4	4	4	4	4	4	4
5	4	4	5	4	5	5	4	5
4	4	4	4	4	4	4	4	4
5	3	5	3	5	3	5	3	5
5	5	5	5	4	4	5	5	5
5	5	4	5	5	4	4	4	4
4	4	4	4	4	3	4	3	4
3	4	5	3	4	5	5	5	5
3	3	3	3	3	3	3	3	3
1	2	3	4	5	4	3	2	1
4	4	4	4	4	4	4	4	4
4	4	4	4	4	4	4	4	4
3	3	4	3	4	4	3	3	4
4	4	4	4	4	4	4	4	4
4	4	4	4	4	3	4	5	5
4	5	4	4	3	4	5	5	5
4	4	4	4	4	4	4	4	4
4	5	4	5	4	5	4	4	5
4	5	5	5	5	5	5	5	5
2	2	2	4	3	3	4	4	5
5	4	4	4	4	5	4	5	4
5	5	5	5	5	5	5	5	5
5	5	4	4	5	4	4	5	5

4	4	4	4	4	4	4	4	4
5	5	5	5	5	5	5	5	5
3	3	4	4	3	3	3	4	4
5	5	5	3	3	3	3	4	4
4	4	4	4	4	4	4	4	4
4	4	4	4	4	4	4	4	4
3	4	4	3	4	3	4	4	3
4	5	5	5	5	5	5	5	3
4	4	4	4	4	4	4	4	4
4	4	4	4	4	4	4	4	4
4	4	4	4	4	4	4	4	4
5	5	5	5	5	5	5	5	5
4	3	4	4	3	3	3	4	3
5	5	5	4	5	4	5	5	5
5	5	5	4	5	4	5	4	5
3	3	3	3	3	3	4	4	4
4	4	4	4	4	4	4	4	4
4	3	4	3	3	3	4	3	3
5	5	5	4	5	4	5	4	5
3	3	3	3	3	3	4	4	4
4	4	4	4	4	4	4	4	4
4	3	4	3	4	3	4	3	4
4	4	4	4	3	3	4	3	4
4	4	4	5	5	4	5	5	5
4	4	4	4	4	4	4	4	4
4	3	3	3	4	4	4	4	4
5	5	5	5	5	5	5	5	5
4	5	4	4	5	5	4	5	4
5	5	3	3	4	4	4	3	4
2	4	4	4	3	3	3	3	4
4	4	4	4	4	4	4	4	4
3	3	3	3	3	3	3	3	3
4	4	4	4	4	4	3	3	4
5	5	5	4	5	5	5	5	4
4	4	4	4	4	4	4	4	3
4	4	3	4	4	3	4	3	4
4	4	4	4	4	4	4	4	4
5	5	4	4	4	5	5	5	4
5	5	4	4	5	5	5	5	5
4	3	4	3	4	3	4	3	4
3	4	4	4	3	4	4	4	4
4	4	4	4	4	4	4	4	4
4	4	4	4	4	4	4	3	4
4	5	4	4	4	5	4	4	4
5	4	3	4	5	4	3	4	5
4	4	4	4	4	4	4	4	5
5	5	5	4	5	5	4	2	4
4	5	4	5	4	5	4	5	4
4	5	5	5	5	4	4	5	4
4	4	4	4	4	4	4	4	4
3	3	3	3	3	3	3	3	3
5	5	5	5	5	5	5	5	5
5	5	5	5	5	5	5	5	5
4	4	4	4	4	4	4	4	4

5	5	5	5	5	5	5	5	5
5	5	5	5	5	5	5	4	5
5	4	5	5	4	5	4	5	5
5	5	5	5	5	5	5	5	4
4	4	4	4	4	4	4	3	4
5	4	5	4	5	5	4	5	5
4	4	4	4	4	4	4	4	4
4	4	4	4	4	4	4	4	4
3	2	3	4	4	3	2	3	3
4	3	4	3	4	3	4	3	4
4	4	4	4	4	4	4	4	4
4	4	4	4	4	4	4	4	4
4	4	4	4	4	4	4	4	4
5	5	5	5	5	5	5	5	5
5	5	5	5	4	4	4	3	4
4	4	4	4	4	4	3	3	4
5	5	5	5	5	5	5	5	5
5	3	3	4	2	4	4	4	4
4	4	4	4	4	4	4	3	4
4	4	4	4	4	4	4	4	4
5	5	3	4	4	3	4	3	5
4	5	5	4	5	5	5	5	5
4	4	4	4	4	4	5	4	4
2	2	3	5	1	1	3	3	3
4	4	3	4	4	3	4	4	4
4	4	4	4	5	5	5	5	5
4	4	4	5	5	5	5	5	5
4	5	5	5	4	4	5	4	4
5	4	5	5	5	5	5	5	5
5	5	5	5	5	5	4	4	5

D18	D19	D20	D21	D22	D23	D24	D25	D26
4	4	4	4	4	4	4	4	4
5	5	5	5	5	5	4	4	5
4	4	5	5	4	4	4	4	4
4	4	5	5	4	4	4	4	4
4	4	5	5	4	4	4	4	4
4	4	5	5	4	4	4	4	4
4	4	4	5	5	4	4	4	4
4	4	4	4	4	4	4	4	3
4	4	5	4	5	4	2	3	4
4	4	4	4	4	4	4	4	4
5	5	5	5	5	5	5	5	5
5	5	5	5	5	5	5	5	5
4	4	4	4	4	4	4	4	4
5	5	5	5	5	5	5	5	2
5	5	5	5	5	5	5	5	2
4	4	5	5	5	5	4	5	4
4	4	4	4	4	4	4	4	4
5	5	5	4	4	4	5	5	4
4	5	4	5	5	5	4	5	5
5	5	4	4	5	5	4	4	4
4	5	5	5	5	4	4	5	5
4	5	5	5	4	2	4	4	5
5	5	5	5	5	5	5	4	5
5	5	5	5	5	5	5	5	5
5	5	5	5	5	5	5	5	5
4	4	4	4	4	4	4	5	5
5	5	5	5	5	5	5	5	5
5	5	5	5	5	5	5	5	5
4	5	5	5	5	5	4	5	4
2	3	4	5	4	5	5	5	5
4	4	4	5	5	5	5	5	5
3	4	5	4	3	4	5	4	4
4	5	5	5	4	4	4	5	5
3	4	4	5	4	4	5	3	4
5	5	5	5	5	5	5	5	5
5	4	5	5	4	4	4	5	5
4	4	4	3	4	4	4	3	4
4	4	4	4	4	4	4	4	4
4	4	4	4	5	4	4	5	5
5	5	5	5	5	5	4	5	5
4	5	5	5	4	5	5	4	4
2	3	4	2	3	3	3	3	3
5	5	5	5	5	5	5	5	5
4	4	4	4	5	5	5	4	5
4	4	4	4	4	4	4	4	4
5	5	5	5	4	5	4	5	4
5	4	5	5	5	5	4	4	4
4	4	4	4	4	5	4	3	3
4	4	4	4	4	5	5	5	5
5	5	4	3	5	5	4	4	4
4	4	5	5	5	5	4	4	4

5	5	5	4	5	3	5	5	5
4	5	5	5	4	4	4	4	5
4	5	4	5	4	5	4	4	4
4	4	4	5	4	3	4	5	4
4	3	3	3	3	3	3	3	4
5	4	3	4	5	4	3	4	4
5	4	4	5	5	4	4	5	4
4	4	4	4	5	5	5	4	4
5	5	5	5	5	4	3	5	5
5	5	5	5	5	5	3	5	5
4	4	4	4	4	4	4	4	4
3	2	2	2	3	2	3	2	2
3	4	4	4	4	4	4	4	4
4	3	4	3	3	4	5	1	2
5	5	5	5	5	5	5	5	5
5	5	5	5	5	5	5	5	5
5	5	5	5	5	5	5	5	5
4	4	4	4	4	4	4	4	4
4	4	4	4	4	4	4	4	4
4	4	5	5	5	3	3	4	4
4	5	5	5	5	4	3	5	5
4	4	5	5	5	4	3	3	4
4	4	5	5	5	5	3	3	4
4	4	5	5	5	4	3	5	5
3	3	3	3	3	3	3	3	3
4	4	4	3	3	3	3	4	4
4	5	4	4	5	5	4	4	5
4	3	4	3	3	3	3	4	3
4	4	4	4	4	4	4	4	4
5	5	5	5	5	5	5	5	5
4	3	3	4	4	4	4	4	4
4	4	4	4	4	4	4	4	4
5	5	5	5	5	5	5	5	5
4	4	4	4	5	4	4	5	4
4	4	4	4	4	4	4	4	4
4	4	5	3	4	4	4	5	4
4	4	4	4	4	4	4	4	4
4	4	4	4	4	4	4	4	2
4	4	4	4	4	4	4	4	4
5	5	5	5	5	5	5	5	5
4	5	4	5	4	5	5	4	5
5	5	5	5	5	5	5	5	5
5	5	5	5	5	5	5	5	5
4	4	5	4	4	4	3	4	4
4	5	5	5	4	5	5	5	4
4	5	5	4	5	4	4	5	4
4	4	4	4	4	4	3	4	4
4	4	4	4	4	4	4	5	5
4	4	4	4	4	4	4	5	4
4	4	4	4	4	4	4	4	4
4	4	4	4	5	4	4	4	5
5	5	5	5	4	5	5	4	5
5	5	5	5	4	5	5	4	5
4	5	5	4	4	5	4	4	4

4	4	4	4	4	4	4	4	4
3	4	4	3	3	3	3	4	3
5	5	5	4	5	5	4	5	5
4	4	4	3	3	3	3	3	3
4	5	4	4	4	4	4	4	4
4	4	4	4	5	4	5	4	5
4	4	3	3	4	3	4	5	5
4	4	4	4	4	3	3	4	4
4	4	4	4	4	3	3	4	4
5	5	5	5	5	5	5	5	5
5	5	5	5	5	5	5	5	5
4	4	4	4	4	3	3	4	4
3	3	3	4	4	4	3	3	3
4	4	4	3	3	4	3	4	4
4	4	4	4	4	4	4	4	4
3	3	3	3	3	3	3	3	2
5	5	5	5	4	5	5	5	4
4	4	5	4	5	5	4	4	4
5	5	5	5	5	4	4	4	4
4	4	5	5	4	5	4	4	5
5	5	4	5	5	5	5	5	4
5	5	5	5	5	5	5	5	4
4	5	4	4	4	4	3	3	3
4	4	4	4	4	4	3	4	4
5	5	5	5	5	5	5	5	5
4	4	4	4	4	4	4	4	4
5	5	5	5	5	5	5	5	5
5	4	4	4	5	4	4	4	4
5	5	5	5	5	5	5	5	5
4	4	4	4	4	4	4	4	4
5	5	5	5	5	5	4	5	4
5	5	5	5	4	4	4	5	5
5	5	5	5	5	4	5	4	5
5	5	5	4	4	4	4	4	4
5	5	5	5	5	5	5	5	5
4	4	4	4	3	4	2	5	5
4	4	4	4	4	4	4	4	4
4	4	4	4	4	4	4	4	4
4	4	4	4	4	4	4	4	4
4	4	4	4	4	4	4	4	4
4	5	5	4	4	3	3	4	4
4	4	4	4	4	4	4	4	4
4	4	4	4	4	4	4	4	4
4	4	4	4	4	4	3	4	4
5	5	5	5	5	5	5	5	5
4	4	4	4	4	3	3	5	4
4	4	4	4	3	4	4	3	4
4	4	4	4	4	3	3	4	4
4	4	4	4	4	4	3	4	4
4	4	4	4	4	4	4	4	4
4	4	4	4	4	4	4	4	4
5	5	4	4	4	4	4	4	4
5	5	5	5	5	5	4	5	5
4	4	4	4	4	4	3	4	4

5	5	4	3	3	4	3	5	5
4	4	4	4	4	4	4	4	4
4	4	4	3	4	4	4	4	4
5	5	5	5	5	5	4	5	4
4	5	5	5	3	2	2	5	2
4	4	4	4	4	4	4	4	4
4	4	4	3	4	4	4	4	4
5	5	4	4	4	4	5	5	5
5	5	5	5	5	4	4	5	4
4	4	5	5	5	5	4	4	3
5	5	5	4	4	4	4	4	4
4	4	4	4	4	4	4	4	4
5	5	5	4	5	5	4	5	5
5	5	5	5	5	5	4	5	5
5	5	5	5	5	5	5	5	5
4	4	4	4	5	3	4	4	3
4	4	4	4	4	4	4	4	4
5	5	5	5	5	5	5	5	5
5	5	5	5	5	5	4	5	3
5	5	5	5	5	5	5	5	5
5	5	5	5	5	4	4	5	5
5	5	5	5	5	4	4	5	5
4	4	4	4	4	4	4	4	4
4	4	4	4	5	5	5	5	5
5	5	5	5	5	5	5	5	5
4	4	4	3	4	4	3	4	3
4	4	5	4	5	4	4	4	3
5	5	5	5	5	5	5	5	5
4	4	4	4	4	4	4	4	4
4	4	5	2	4	4	2	4	4
4	4	4	4	4	4	3	4	4
4	4	5	5	5	5	4	5	5
5	5	5	4	4	4	4	4	5
5	5	5	5	5	5	5	5	5
4	4	4	4	4	4	4	4	4
4	5	5	3	4	4	5	4	3
5	5	5	5	5	5	5	5	5
5	5	5	5	5	5	5	5	5
4	5	5	4	4	4	3	4	3
3	2	4	3	3	4	4	3	3
5	5	5	4	5	4	4	5	4
4	5	5	5	4	4	4	5	5
4	4	5	5	5	4	4	5	4
4	4	4	4	4	4	4	4	4
5	5	5	5	5	5	5	5	5
5	5	5	5	5	5	5	5	5
4	4	4	4	4	4	4	4	4
4	4	4	4	4	4	4	4	4
4	4	4	4	4	4	4	3	3
5	5	5	4	5	3	3	5	5
4	4	4	4	4	4	4	4	5
4	4	4	4	4	3	4	4	4
5	5	5	5	5	5	5	5	5
4	4	5	4	4	3	3	5	2

4	4	4	4	4	4	3	4	4
4	5	5	5	5	4	5	4	5
4	5	4	4	5	5	5	5	4
4	4	4	4	4	4	4	4	4
4	4	3	5	4	3	3	5	3
4	4	4	4	4	4	4	4	4
5	5	5	5	2	5	5	5	5
4	4	5	4	4	4	3	3	3
4	4	4	4	4	4	4	4	4
5	5	5	4	4	5	4	5	5
4	4	5	4	4	4	4	5	4
4	4	4	4	4	4	3	4	4
4	3	3	4	5	3	4	5	4
5	4	5	5	5	5	5	4	5
5	5	5	5	5	5	5	5	5
4	4	4	4	4	4	3	4	3
4	4	5	4	3	3	3	4	4
4	4	4	4	4	4	4	4	4
4	5	5	5	5	5	4	5	5
4	4	4	4	4	4	3	5	4
4	4	4	4	4	4	4	4	4
3	3	4	4	4	4	4	4	4
4	3	4	3	3	3	4	4	3
4	4	4	4	4	4	4	4	4
4	4	4	4	4	4	4	4	4
4	4	5	5	5	5	3	4	4
4	4	4	4	4	4	5	5	4
4	4	4	4	4	4	4	4	4
5	5	5	5	5	5	5	5	5
4	4	4	5	5	5	5	5	4
4	4	4	4	4	4	4	4	4
4	5	5	4	4	4	4	5	4
5	5	5	5	5	5	5	4	4
4	4	4	4	4	4	4	4	4
3	3	3	3	3	3	3	3	3
5	5	5	5	5	5	5	5	5
4	4	4	5	5	4	4	5	5
4	4	4	4	4	4	4	5	4
4	4	4	5	4	5	4	4	4
4	4	4	4	4	4	4	4	4
2	3	4	5	5	4	3	2	1
4	4	4	4	4	4	4	4	4
4	4	4	4	4	4	4	4	4
4	4	4	4	4	4	4	5	4
4	4	4	4	4	4	4	4	3
5	5	5	5	5	3	3	4	5
5	5	5	5	5	3	4	4	5
4	4	4	4	4	4	4	4	4
4	4	4	4	4	4	5	5	5
5	5	5	5	5	5	5	5	5
4	5	5	5	5	4	4	5	3
5	5	4	5	4	5	5	4	5
5	5	5	5	5	5	5	5	5
4	4	4	4	4	4	5	5	4

5	5	5	4	4	4	5	4	4
5	5	5	5	5	5	5	5	5
3	3	4	4	3	4	4	4	4
4	4	5	5	5	5	4	4	4
4	4	5	5	5	5	5	5	5
4	4	4	4	4	4	4	4	4
4	4	4	4	3	4	3	4	3
4	4	5	5	4	3	2	5	5
5	5	4	5	5	4	5	5	5
4	4	4	4	4	4	4	4	4
5	5	5	5	5	5	5	5	5
4	5	5	5	5	5	5	5	5
3	3	3	3	3	3	3	3	4
4	4	4	4	4	4	4	4	4
4	4	4	4	4	4	4	4	4
4	4	4	4	4	4	4	4	4
4	3	3	3	3	3	3	4	4
4	4	4	4	4	5	5	5	5
4	4	4	4	4	4	4	4	4
4	4	4	4	4	4	4	4	4
4	3	3	3	3	3	3	4	4
1	3	4	3	2	3	4	3	4
4	4	4	4	4	4	4	4	4
4	4	5	5	4	5	4	5	4
4	4	4	4	4	4	4	4	4
4	4	4	4	4	3	3	4	4
5	5	5	5	5	5	5	5	5
4	5	4	5	5	5	5	5	4
5	5	5	5	4	4	4	5	4
4	4	5	5	5	4	4	5	5
4	4	4	4	4	4	4	4	4
3	3	3	3	3	3	3	3	3
4	3	3	4	4	4	4	4	2
5	5	5	4	5	5	5	5	4
3	4	4	4	4	4	4	4	3
3	4	3	4	4	3	4	3	4
4	4	5	4	5	5	5	5	4
5	4	5	5	5	5	5	5	4
5	5	5	5	5	5	5	3	4
3	3	4	3	4	3	2	3	2
4	4	4	4	4	4	4	4	4
4	4	4	4	4	4	4	4	4
4	4	4	4	4	4	4	5	4
4	4	4	4	5	4	5	4	5
5	4	4	4	4	4	4	4	4
5	4	4	5	5	5	5	5	5
4	5	4	4	4	5	5	5	4
5	4	5	4	5	4	5	4	5
5	4	5	5	5	5	5	5	5
4	4	4	4	4	4	4	4	4
3	3	3	3	3	3	3	3	3
5	5	4	5	4	3	4	3	2
5	5	5	5	5	5	5	5	5
4	4	4	4	4	4	4	4	4

5	5	5	5	5	5	5	5	5
5	5	5	5	5	5	5	5	5
5	4	4	4	5	4	4	5	4
5	4	5	4	5	4	5	4	5
4	4	4	4	3	3	4	3	4
5	4	4	4	4	4	4	4	4
4	4	4	4	4	4	4	4	4
4	4	4	4	4	4	4	4	4
2	4	4	4	4	4	4	4	4
4	4	3	3	3	3	3	3	3
4	5	4	4	4	4	4	3	4
4	4	4	3	4	4	4	4	4
4	4	4	4	4	4	4	4	4
5	5	5	5	5	5	5	5	5
4	4	4	4	4	4	4	4	4
4	4	3	4	3	4	3	4	3
5	5	5	5	5	5	5	5	5
4	4	5	5	4	5	4	5	5
4	4	4	4	4	3	4	4	3
4	4	4	4	4	4	4	4	3
4	4	3	4	4	5	4	3	5
4	4	5	5	4	4	5	5	4
4	4	4	4	4	4	4	2	3
3	3	3	3	3	1	3	3	3
3	4	4	4	4	4	4	4	4
5	5	5	5	5	5	5	5	3
4	4	4	3	4	3	3	3	4
4	4	5	5	5	5	5	5	5
5	5	5	5	5	5	5	5	5
4	4	4	4	4	5	5	5	5

D27	D28	D29	D30	D31	D32	psychological capital(D)
4	4	4	4	4	4	4.53
4	3	3	4	4	3	3.59
4	4	4	4	4	4	4.25
4	4	4	4	4	4	4.25
4	4	4	4	4	4	4.28
4	4	4	4	4	4	4.28
4	4	4	4	4	4	4.28
3	3	4	3	4	3	3.56
4	4	4	3	4	4	3.75
4	3	3	3	3	3	3.59
4	5	4	5	4	5	4.69
4	5	5	5	2	5	4.63
4	4	4	4	4	4	3.97
5	5	4	3	4	4	4.16
5	5	4	3	4	4	4.16
4	5	4	3	4	5	4.31
3	4	4	4	4	4	3.97
3	4	4	3	4	5	4.31
4	5	4	5	4	5	4.28
4	4	5	4	4	4	4.16
5	5	5	4	4	4	4.56
5	4	2	2	3	3	4.19
5	5	5	5	5	5	4.72
5	5	5	5	5	5	4.75
5	5	5	5	5	5	4.69
4	5	4	4	4	4	4.06
2	5	5	5	5	5	4.41
5	5	4	4	4	5	4.72
4	4	4	3	3	3	4.31
4	4	4	3	4	5	3.88
4	5	4	5	4	5	4.47
4	5	5	4	4	3	4.09
5	4	4	5	5	4	4.47
5	4	3	4	5	4	3.97
5	5	5	2	5	5	4.72
4	4	5	4	5	5	4.56
4	4	4	4	4	4	3.69
4	4	4	4	4	4	3.94
5	3	5	4	5	3	4.03
4	4	5	5	5	5	4.84
4	5	5	4	4	5	4.44
3	3	3	3	3	3	3.16
5	5	5	5	5	5	4.97
5	4	5	4	4	4	4.38
4	4	4	4	4	4	3.88
4	4	4	4	5	4	4.47
4	5	5	4	5	5	4.44
4	4	3	3	4	5	4.03
5	5	4	4	5	5	4.41
4	4	4	5	5	5	4.25
5	5	5	5	5	5	4.25

4	4	4	5	5	5	4.28
5	4	5	4	4	4	4.34
4	5	5	5	4	4	4.50
4	5	4	4	4	5	4.13
3	3	3	3	3	3	3.28
4	4	3	4	5	4	3.97
5	5	5	5	5	5	4.56
4	4	4	4	5	5	4.44
5	5	5	5	5	4	4.25
5	5	3	3	3	5	4.31
4	4	4	4	4	4	3.94
3	2	3	2	3	2	2.47
4	3	2	3	3	2	3.34
3	4	2	4	3	4	3.25
5	5	5	5	5	5	4.88
5	5	5	5	5	5	4.47
5	5	5	5	5	4	4.97
4	4	4	4	4	4	3.97
5	5	3	3	3	4	3.97
3	3	3	2	3	3	3.13
5	5	5	4	5	4	4.47
4	5	5	4	4	4	4.22
4	3	3	3	3	3	3.22
4	5	5	4	4	3	4.22
3	3	3	3	3	3	3.06
4	4	4	4	4	3	3.84
5	5	4	4	5	4	4.50
4	4	4	4	4	4	3.69
4	4	4	2	2	2	3.41
5	5	5	3	3	2	4.41
4	4	4	4	4	2	3.75
4	4	4	4	4	4	3.91
5	5	5	5	5	5	4.88
5	4	5	4	5	4	4.34
4	4	4	3	3	3	3.72
4	4	4	4	4	4	4.09
4	4	4	4	4	4	3.47
4	4	4	4	4	4	4.03
4	4	4	4	4	4	3.50
5	5	5	5	5	5	4.44
4	5	4	5	5	4	4.50
5	5	4	3	4	2	4.06
5	5	4	4	4	4	4.25
4	5	4	4	3	4	4.03
5	5	5	4	4	5	4.53
4	4	4	4	5	4	4.16
4	4	3	4	4	5	3.97
5	5	4	3	4	4	4.06
4	4	4	4	4	4	4.00
4	4	4	4	4	4	3.97
4	5	4	5	4	5	4.41
5	5	5	4	4	5	4.50
5	5	5	4	4	5	4.50
4	4	4	4	4	3	3.97

4	4	4	3	4	3	3.91
3	4	4	3	3	4	3.25
4	5	5	5	4	5	4.78
3	4	3	3	3	3	3.28
4	4	4	4	4	5	4.09
4	5	4	5	5	5	3.91
4	4	3	4	4	4	3.88
4	3	4	3	4	3	3.47
4	3	4	3	4	3	3.59
5	5	5	5	5	5	4.91
5	5	4	4	4	4	4.16
4	3	4	3	4	3	3.59
4	4	4	3	3	3	3.44
4	4	4	4	4	4	3.81
4	4	4	4	4	4	3.94
2	2	2	2	2	2	2.38
5	5	4	5	4	5	4.75
5	4	4	5	4	4	4.31
5	5	4	5	4	4	4.50
4	5	5	5	4	4	4.19
5	5	5	4	5	5	4.78
4	4	4	5	5	5	4.88
3	5	5	5	5	5	4.34
4	4	4	2	2	2	3.47
5	5	5	5	5	5	4.81
4	4	4	4	4	4	3.97
5	5	4	4	5	5	4.75
5	5	4	4	4	4	4.13
5	5	5	5	5	5	4.59
4	4	4	4	4	4	3.91
5	5	5	5	5	5	4.75
4	5	4	4	4	4	4.69
5	4	5	5	5	5	4.66
5	4	4	5	4	4	4.22
5	5	5	5	5	5	4.72
5	5	3	1	2	1	3.03
4	4	4	4	4	4	3.88
2	4	4	4	4	4	3.75
4	4	4	3	4	3	4.03
4	4	4	4	4	4	3.97
4	4	4	3	4	3	3.84
4	4	4	4	4	4	4.09
4	4	4	4	4	4	4.09
4	4	4	3	3	4	3.81
5	5	5	4	5	5	4.81
5	4	4	3	3	3	3.88
3	4	4	4	3	4	3.66
4	4	4	4	1	4	3.84
4	4	4	3	4	4	3.84
4	4	4	4	4	4	4.28
4	4	4	4	4	4	3.91
4	4	4	4	4	4	3.97
3	4	4	4	4	4	4.31
4	4	4	3	4	3	3.56

5	5	5	5	5	5	3.66
4	4	4	4	4	4	4.00
3	4	4	3	4	4	3.75
5	4	4	3	3	4	4.25
3	4	3	3	4	3	3.50
4	4	4	4	3	4	3.91
4	4	4	4	4	4	3.88
4	5	5	4	4	4	4.47
5	5	3	3	4	3	4.41
4	5	4	3	1	2	3.97
4	5	4	4	4	4	4.19
4	4	4	4	4	4	3.97
4	5	5	4	4	4	4.25
5	5	5	5	5	5	4.66
5	5	5	5	5	5	4.69
4	4	4	3	3	3	3.38
4	4	4	4	4	4	4.00
5	5	5	5	5	4	4.69
4	4	3	2	3	4	3.81
5	5	5	5	5	5	5.00
5	5	4	4	4	4	4.38
5	5	4	4	4	4	4.38
4	4	4	4	4	4	3.94
5	5	5	5	5	5	4.09
4	5	5	5	5	5	4.72
4	4	3	3	3	2	3.63
4	4	4	4	4	4	3.94
5	5	5	5	5	5	4.72
4	4	4	3	4	2	3.81
4	4	4	2	2	2	3.31
4	4	4	3	4	3	3.69
5	5	5	5	5	3	4.41
5	5	5	4	5	5	4.19
5	5	5	5	5	5	4.66
4	4	4	3	4	3	3.84
3	5	4	4	4	4	4.03
5	5	5	5	5	5	5.00
5	5	5	4	4	4	4.66
3	3	3	2	2	2	3.44
4	3	3	3	4	5	3.19
4	5	5	3	4	4	4.09
4	4	4	5	4	5	4.34
4	4	4	4	4	4	3.97
4	4	4	4	4	4	4.00
5	5	5	5	5	5	4.31
5	5	5	5	5	5	4.97
4	4	3	2	4	3	3.41
4	4	4	4	4	4	3.75
3	3	3	3	4	4	3.56
5	5	4	3	4	4	4.09
5	4	5	4	5	5	4.16
4	4	4	4	4	4	4.41
5	5	5	5	5	5	4.91
3	3	3	3	3	3	3.47

4	4	4	4	4	4	3.97
4	5	4	5	4	5	4.44
4	4	5	4	4	4	4.28
4	4	4	4	4	4	4.53
3	4	5	3	4	4	3.69
4	4	4	4	4	4	3.91
5	5	4	5	5	5	4.81
3	4	4	3	4	4	3.66
4	4	4	4	4	4	4.06
5	5	5	5	5	5	4.38
4	4	4	4	4	4	4.44
4	4	3	3	3	3	3.84
3	3	4	4	4	4	3.59
4	4	4	4	4	5	4.63
3	4	5	5	5	5	4.81
4	3	4	3	4	3	3.69
4	3	4	3	3	3	3.41
4	4	4	4	4	4	4.00
4	5	4	3	4	4	4.31
4	4	3	2	2	2	3.31
4	4	4	4	4	4	4.00
4	4	4	4	4	4	3.66
4	4	3	4	3	4	3.53
4	4	4	3	4	3	3.72
4	4	4	3	4	4	3.69
4	3	5	4	4	3	4.06
4	4	4	5	4	4	4.16
4	4	4	4	4	4	4.00
5	5	5	5	5	5	4.47
4	4	4	4	4	5	4.53
5	4	4	5	5	4	4.53
4	4	4	4	4	4	4.09
4	5	4	4	5	5	4.56
4	4	4	4	4	4	3.94
3	3	3	3	3	3	3.50
5	5	5	5	5	5	4.63
5	5	5	4	4	4	4.25
4	4	4	3	3	3	3.81
5	5	5	4	4	4	4.25
4	4	4	4	4	4	3.56
2	3	4	5	1	1	2.94
4	4	4	4	4	4	3.94
4	4	4	4	4	4	3.91
4	5	3	4	4	4	3.81
4	4	4	4	4	4	3.97
5	5	4	4	3	4	4.19
5	5	4	4	3	4	4.28
4	4	4	4	4	4	3.97
4	5	4	5	4	5	4.41
5	5	5	5	5	5	4.75
4	5	4	2	4	4	3.75
4	5	5	4	5	5	4.50
5	5	5	5	5	5	5.00
4	4	5	4	4	4	4.28

4	4	4	5	5	5	4.22
5	5	5	5	5	5	5.00
4	3	3	4	4	4	3.59
5	5	5	4	4	4	4.16
5	5	5	5	5	5	4.41
4	4	4	4	4	4	4.00
4	3	4	3	4	4	3.66
5	5	5	5	5	5	4.41
5	5	4	4	4	4	4.28
4	4	4	4	4	4	4.00
5	5	5	5	5	5	4.47
5	5	5	4	5	5	4.91
4	4	4	3	3	4	3.44
3	3	3	3	4	4	4.16
4	4	4	4	4	4	4.34
4	4	4	4	4	4	3.69
4	3	3	4	4	4	3.75
5	5	5	5	5	5	4.00
4	4	4	4	4	4	4.34
4	4	4	4	4	4	3.69
4	3	3	4	4	4	3.75
3	4	4	3	3	3	3.16
4	4	4	4	4	4	3.78
4	4	5	4	5	4	4.44
4	4	4	4	4	4	3.84
3	4	3	4	3	3	3.72
5	5	5	5	5	5	4.88
4	4	4	4	4	4	4.44
4	5	4	2	3	3	4.03
5	5	4	2	5	3	3.84
4	4	4	4	4	4	3.97
3	3	3	3	3	3	3.00
3	4	4	4	2	4	3.72
4	5	4	5	4	5	4.75
4	3	4	3	4	3	3.78
3	4	4	4	3	4	3.63
4	5	4	5	4	4	4.22
4	4	4	4	4	5	4.66
5	5	5	5	3	5	4.59
4	3	2	3	2	4	3.25
4	4	4	4	4	4	3.69
4	4	4	4	4	4	4.00
4	4	4	4	4	5	4.00
4	5	4	4	4	4	4.16
4	4	4	4	4	4	4.06
5	5	5	5	5	5	4.44
3	4	4	4	3	5	4.13
4	5	4	5	4	5	4.44
5	4	5	5	5	5	4.50
3	4	4	4	4	4	3.97
3	3	3	3	4	4	3.06
4	4	5	5	1	4	4.25
5	5	5	5	5	5	4.94
4	4	4	4	4	4	3.78

5	5	5	5	5	5	5.00
5	5	5	5	1	5	4.69
5	5	4	5	4	4	4.50
4	5	5	5	5	5	4.81
3	4	2	4	4	4	3.84
4	4	4	4	3	4	4.41
4	4	3	4	2	4	3.75
4	4	4	4	4	4	4.00
4	3	3	4	4	4	3.44
4	4	3	3	4	4	3.47
4	4	4	4	2	5	3.78
4	4	4	4	4	4	3.97
4	4	4	4	4	4	4.00
5	5	5	5	5	5	4.94
4	4	4	4	2	5	3.78
4	3	4	3	4	4	3.69
5	5	5	5	5	5	5.00
4	4	4	4	4	5	4.09
3	4	4	4	2	4	3.72
3	4	4	4	4	4	3.91
5	3	5	3	5	4	4.03
4	5	5	5	4	4	4.59
4	3	3	3	3	4	3.78
3	5	4	4	1	4	2.88
4	4	3	4	4	4	3.84
4	4	4	4	4	4	4.63
4	3	3	4	3	3	3.81
4	4	5	5	5	5	4.50
5	5	5	5	5	5	4.94
4	4	4	4	2	4	4.25
